# Climate change reshapes the global distribution of wild barley relatives: integrating life-history traits and ecological niche modeling

**DOI:** 10.3389/fpls.2026.1935270

**Published:** 2026-08-25

**Authors:** Xue Yang, Jian Zhang, Xiao-ping Tan, Yan-li Xiong, Xiao-ya Chen, Yu-tong He, Yun-fan Zhang, Hao-jun Wang, Xiao Ma, Yi Xiong, Qing-qing Yu

**Affiliations:** 1 Sichuan Academy of Grassland Sciences, Chengdu, Sichuan, China; 2 College of Grassland Science and Technology, Sichuan Agricultural University, Chengdu, Sichuan, China; 3 Sichuan Provincial Research Center for Forestry and Grassland Development, Chengdu, Sichuan, China

**Keywords:** climate change, CMIP6, crop wild relatives, Hordeum, Maxent modeling, migration pathways, niche breadth, suitable habitat

## Abstract

**Introduction:**

Wild *Hordeum* species are crucial genetic resources for climate‑resilient crop breeding. Clarifying their responses to climate change is essential for germplasm conservation and sustainable crop improvement. This study aims to elucidate the divergent climatic responses of wild *Hordeum* species and provide scientific evidence for germplasm protection and climate‑adaptive crop breeding.

**Methods:**

This study was conducted at a global scale, covering the major distribution areas and diversity hotspots of wild barley. We combined life‑history traits, niche breadth, and interspecific habitat overlap, and adopted MaxEnt species distribution models with CMIP6 climate datasets to project the spatiotemporal dynamics of suitable habitats for 13 wild Hordeum species. The ENMeval package was used for systematic parameter calibration and sensitivity analysis to optimize model configurations and ensure reliable predictive performance.

**Results:**

The model showed strong performance (mean AUC = 0.907 ± 0.029, TSS = 0.763 ± 0.064). Four global diversity hotspots were identified: southwest and central Asia, the Mediterranean region, western North America, and the Andes Mountains. Niche breadth and climate vulnerability are phylogenetically structured, with closely related species sharing similar ecological constraints and extinction risks. By 2100, wild *Hordeum*’s global suitable habitat is projected to contract by ~62%, with annual species suffering greater losses than perennials. *Hordeum spontaneum* will suffer the most severe range contraction (97.83% habitat loss, threatening wild population persistence), while *Hordeum muticum* shows moderate resilience (36.96% reduction) and *Hordeum agriocrithon* will expand by 34.84%. Habitat migration is primarily driven by precipitation gradient variation, and stable niche overlap among closely related species verifies the edge adaptation hypothesis.

**Discussion:**

Wild *Hordeum* species exhibit divergent climate change responses. These contrasting habitat change trajectories signal divergent conservation urgency: extreme contractions threaten genetic erosion in crop wild relatives, whereas expanding ranges highlight potential donor species for climate‑adaptive breeding. High‑risk taxa such as *Hordeum stenostachys* require urgent coordinated *in‑situ* and *ex‑situ* conservation to prevent population decline and genetic erosion. In contrast, range‑expanding species (*Hordeum agriocrithon*) offer insights into adaptive mechanisms, with practical implications for climate‑resilient crop breeding. Notably, these projections reflect climatic suitability under a single climate model and should be interpreted cautiously given unmodeled biotic interactions and dispersal constraints.

## 1 Introduction

In recent decades, climate change has increased the frequency and intensity of extreme weather events (Eichelmann et al., 2025), such as heatwaves, droughts, and heavy rainfall. These changes have in turn accelerated shifts in species distribution patterns, contributed to biodiversity loss, habitat fragmentation, and posed a serious threat to global agriculture (Olabode et al., 2025). Crop wild relatives (CWRs) represent natural gene pools of crop genetic diversity, harboring key adaptive traits such as drought tolerance, salt tolerance, and disease resistance (Langridge et al., 2021). However, climate-induced changes in niche suitability are threatening CWRs, leading to shrinking distribution ranges, habitat fragmentation, and migration to higher latitudes or altitudes (Sharma et al., 2021). In this context, analyzing the ecological niche of representative CWRs is essential for unlocking their adaptive potential and guiding climate-resilient breeding efforts. Hordeum L., comprising more than 30 predominantly wild species, represents an important genus of CWRs in Triticeae, Poaceae. The genus encompasses extraordinary cytogenetic diversity, including diploid, tetraploid, and hexaploid species with annual and perennial life histories, and all known genome types (H, I, Xa, Xu) (Du and Li, 2025). This diversity provides a unique opportunity to examine how ploidy level and life history strategies mediate climate vulnerability—insights fundamental to predictive conservation biogeography. Most importantly, these species possess excellent adaptive traits and exhibit a wide distribution across temperate and subtropical regions worldwide (Ro et al., 2024). Thus, leveraging the adaptive potential of wild Hordeum species has become a critical strategy for enhancing climate resilience in barley breeding.

Research on the niche and climate change response of CWRs has become a major focus in biological resource conservation and crop breeding. Meanwhile, rapid advances in species distribution models and molecular biology have provided technical support for studying these resources. The Maximum Entropy Model (MaxEnt) is a classic method in ecological niche modeling (ENM). It can predict habitat suitability using only species presence data. It also analyzes complex interactions between environmental factors effectively. MaxEnt has been successfully applied to many CWRs, forages or invasive species such as Prunus pseudocerasus (Lv et al., 2025), Lilium lancifolium (Herrando-Moraira et al., 2019), Astragalus membranaceus (Yang et al., 2020) and Trifolium repens (Wang et al., 2025), revealing their future potential distribution and core niche-driving factors. Nevertheless, most existing studies focus on single-species habitat suitability (Jin et al., 2024; Lv et al., 2025), lacking comparative analyses of multi-species distribution dynamics across the genus’s full cytogenetic diversity. In particular, how factors such as ploidy, genome type, and life history strategies mediate climate response remains poorly understood.

In this study, we selected 13 representative wild Hordeum species that encompass the full cytogenetic diversity of the genus (Khokhar et al., 2020; Del Pozo et al., 2024) to systematically investigate their ecological niche and responses to climate change. All of these species are undomesticated wild types, which are widely distributed across Eurasia, North America, South America, and other regions. They are primarily diploid, with some polyploid variations. These species fully reflect the intraspecific variation and adaptation within the genus, providing a comprehensive basis for subsequent research.

We characterized the current potential distribution of each species, identified global hotspots of species richness, and quantified niche width and interspecific niche overlap. Future distribution shifts, migration trajectories of distribution centers, and patterns of niche reorganization were projected for the 2040s, 2060s, and 2080s under four climate scenarios (SSP1-2.6, SSP2-4.5, SSP3-7.0, SSP5-8.5). We further examined how life-history traits, including lifespan, reproductive mode, and ploidy level, shape species-specific vulnerability to climate change. We delineated priority conservation areas and corresponding strategies, offering a theoretical foundation for the systematic conservation and sustainable utilization of these important genetic resources. Collectively, this work holds significant scientific and practical value for enhancing the climate resilience of the barley industry and safeguarding food security.

Climate-driven distribution shifts have been widely documented in plant species, and the application value of ecological niche modeling (ENM) for crop wild relative (CWR) conservation is well established. However, critical knowledge gaps persist: few comparative studies incorporate taxa covering the complete cytogenetic diversity of a single genus to evaluate trait-mediated niche differentiation. To fill these gaps, the present study conducts a genus-wide niche comparison among 13 wild *Hordeum* species. Its novelty resides in linking ploidy variation and life-history strategies to interspecific divergence in climate sensitivity. Beyond predicting habitat dynamics under multiple climate scenarios, this work aims to identify the key life-history correlates underpinning heterogeneous climate responses among congeneric wild barley relatives. The outputs are expected to inform differentiated germplasm conservation planning and support the development of climate-resilient barley varieties, thereby contributing to global food security.

## 2 Materials and methods

### 2.1 Acquisition and spatial thinning of species occurrence records

This study focused on 13 representative species of the *Hordeum* genus (**Table 1**). Species distribution records were compiled from two authoritative databases: the Global Biodiversity Information Facility (GBIF, https://www.gbif.org) and the Chinese Virtual Herbarium (CVH, https://www.cvh.ac.cn) (**Supplementary Table 1**; **Supplementary Figure 1**).

**Table 1:** Biological characteristics and distribution sample statistics of 13 Hordeum species.

Species	Life history	Ploidy	Genotype	Number of sample points
*Hordeum agriocrithon* A. E. Åberg	Annual	Hexaploid	I	37
*Hordeum bogdanii* Wilensky	Perennial	Diploid	H	80
*Hordeum* brevisubulatum (Trin.) Link	Perennial	Polyploid	H	273
*Hordeum bulbosum* L.	Perennial	Diploid/Tetraploid	I	177
*Hordeum californicum* Covas & Stebbins	Annual	Diploid	H	51
*Hordeum chilense* Roem. & Schult.	Annual	Diploid	H	35
*Hordeum marinum* Huds.	Annual	Diploid/Tetraploid	Xa	293
*Hordeum murinum* L.	Annual	Diploid/Tetraploid	Xu	596
*Hordeum muticum* J.Presl	Perennial	Diploid	H	51
*Hordeum pusillum* Nutt.	Annual	Diploid	H	146
*Hordeum roshevitzii* Bowden	Perennial	Diploid	H	66
*Hordeum spontaneum* K. Koch	Annual	Diploid	I	92
*Hordeum stenostachys* Godr.	Perennial	Diploid	H	57

To address potential sampling bias and reduce spatial autocorrelation, occurrence records were filtered based on three criteria: (1) geographic plausibility—records outside the known distribution range were excluded; (2) coordinate validity—records with missing or erroneous coordinates were removed; and (3) duplicate removal. The filtered records were then projected onto a 2.5° × 2.5° geographic grid, retaining one record per cell (Steen et al., 2021). This spatial thinning was performed using the dplyr package in R. The 2.5° resolution was selected to balance sufficient point retention for robust MaxEnt modeling with mitigation of geographic clustering bias from uneven herbarium sampling (Kass et al., 2021).

### 2.2 Acquisition, preprocessing, and collinearity screening of environmental variables

Environmental variables, including 19 bioclimatic variables and elevation, were obtained from the WorldClim database (https://worldclim.org) (**Supplementary Table 2**). All datasets were standardized to a spatial resolution of 2.5 arc-minutes (~4.5 km at the equator). Baseline climate data represented average conditions from 1970 to 2000. These data were sourced from WorldClim v2.1 (Villacañas et al., 2026). Future climate projections came from the BCC-CSM2-MR Earth System Model developed by the National Climate Center of China. The projections followed four Coupled Model Intercomparison Project Phase 6 (CMIP6) Shared Socioeconomic Pathway (SSP) scenarios: SSP1-2.6 (low emissions), SSP2-4.5 (moderate emissions), SSP3-7.0 (high emissions), and SSP5-8.5 (very high emissions) (Gashaw et al., 2024). The study period covered 2021–2100. Each SSP scenario included three standard periods: 2041–2060, 2061–2080, and 2081–2100. This division followed CMIP6 protocols and conventional settings for species distribution studies.

A two-stage screening strategy was implemented to reduce multicollinearity among predictors. First, a Pearson correlation matrix (**Supplementary Figure 2**) was computed for all 20 candidate variables. Pairs with |r| > 0.8 were identified, and one variable from each highly correlated pair was removed, prioritizing variables with stronger ecological relevance to grassland species distribution (Chang et al., 2022). Second, Variance Inflation Factor (VIF) analysis was applied to the remaining variables. Variables with VIF > 10 were iteratively removed (Yan et al., 2020; Wu K. et al., 2024), resulting in a final set of 10 key predictors: bio2, bio3, bio8, bio9, bio13, bio14, bio15, bio18, bio19, and elevation (**Supplementary Table 3**).

For niche analysis, we adopted a stricter threshold (VIF < 5) to further reduce redundancy and enhance interpretability. This step retained six core variables: bio2, bio14, bio15, bio18, bio19, and elevation. These variables represent two key ecological dimensions-temperature variability (bio2, bio15) and water stress (bio14, bio18, bio19, elevation)—which are ecologically meaningful for defining the climatic niche of *Hordeum* species (**Supplementary Figure 3**) (Egg et al., 2025).

### 2.3 MaxEnt model construction, parameter optimization, and accuracy assessment

Species distribution projections were generated using MaxEnt v3.4.4k, a presence-only modeling algorithm that estimates potential suitable habitats based on the principle of maximum entropy (Vasconcelos et al., 2024). Systematic optimization of the regularization multiplier (RM) and feature class (FC) was performed using the ENMeval package in R (Adane, 2024). The optimal parameter combination—balancing maximum model fit and minimum complexity (**Supplementary Figure 4**)—was selected based on the corrected Akaike Information Criterion (AICc) (Kass et al., 2021), thereby minimizing the risk of overfitting.

Models were calibrated using 75% of the occurrence records, with the remaining 25% reserved for independent testing. Ten-fold cross-validation (10 replicates) was employed to ensure robustness. Model uncertainty was quantified as the mean ± standard deviation of AUC and TSS across replicates. Model performance was evaluated using two complementary metrics: the Area Under the Receiver Operating Characteristic Curve (AUC) for assessing discriminatory ability, and the True Skill Statistic (TSS) for evaluating threshold-dependent accuracy (Çorbacıoğlu and Aksel, 2023; Kim and Shin, 2026). Only models meeting rigorous performance thresholds (AUC > 0.8; TSS > 0.6) were retained for subsequent analyses (Lin et al., 2025). Sensitivity of model predictions to individual predictors was assessed using permutation importance and jackknife tests. The 10th percentile training presence threshold was used to convert continuous suitability into binary predictions. This threshold is conservative and robust to potential georeferencing errors in occurrence records. Compared with equal sensitivity-specificity and maximum sensitivity plus specificity thresholds, the 10th percentile provided more stable predictions across replicates and better aligned with known *Hordeum* distributions.

### 2.4 Habitat suitability classification and niche dynamics analysis

The continuous suitability index output by MaxEnt was reclassified into five ordinal categories using the Jenks Natural Breaks Optimization method: unsuitable, lowly suitable, moderately suitable, moderately highly suitable, and optimally suitable zones (Tao et al., 2024). This classification scheme enabled precise quantification of changes in habitat area across different climate scenarios. Niche breadth was quantified by calculating the weighted Shannon-Wiener index from suitability rasters under current and future climate conditions; higher standardized values indicate a broader niche breadth and thus greater adaptive capacity to heterogeneous environments. Temporal niche dynamics were assessed using Kernel Density Estimation (KDE) and Horn’s overlap index (range: 0–1) (Eckrich et al., 2020) to evaluate the similarity of niche distributions between current and future scenarios, thereby quantifying niche conservatism versus niche shift (Zhao et al., 2021). To quantify the spatial migration characteristics of species suitable habitats, this study used climate environmental grids and adopted the weight of habitat suitability grids to calculate the latitude-longitude weighted centroids of suitable areas in different periods. We compared centroid coordinates across multiple periods to obtain migration distance and direction. Euclidean distances and directional angles between the centroids of current and future suitable habitats were further measured. A four-quadrant classification method was applied to characterize migration trajectories, so as to clarify the spatial migration patterns of species ranges under climate change (Cong et al., 2020).

Statistical analyses. Differences in niche breadth between annual and perennial species were assessed using the two-tailed Wilcoxon rank-sum test, with effect size reported as rank-biserial correlation (r). Normality of distribution was evaluated using the Shapiro-Wilk test. Bivariate associations between niche breadth and niche overlap were examined using Spearman and Pearson correlation coefficients; the latter was reported for comparative purposes where bivariate normality assumptions were violated. Statistical significance was set at α = 0.05.

## 3 Results

### 3.1 Assessment of MaxEnt model prediction accuracy

Systematic parameter optimization using the ENMeval package in R enabled the construction of high-precision species distribution models for all 13 *Hordeum* species. Performance evaluation (**Supplementary Table 4**; **Supplementary Figure 4**) confirmed excellent predictive accuracy across all models. Specifically, the mean True Skill Statistic (TSS) across all species was 0.763 (± 0.064), substantially exceeding the 0.7 threshold for “good” predictive performance and demonstrating the models’ reliability in in discriminating suitable from unsuitable habitats. Meanwhile, the mean Area Under the Receiver Operating Characteristic Curve (AUC) reached 0.907 (± 0.029), with all models meeting or exceeding the 0.9 benchmark for “excellent” prediction ability (**Table 2**). These robust validation metrics indicate that the parameter-optimized MaxEnt models can accurately simulate the geographic distribution patterns of *Hordeum* species, providing a solid empirical foundation for subsequent analyses of habitat suitability, niche characteristics, and future distribution dynamics.

**Table 2:** Summary of evaluation metrics and optimal parameters of MaxEnt models for 13 *Hordeum* species.

Species	TSS	AUC	FC	RM
*Hordeum agriocrithon* A. E. Åberg	0.889	0.906	LQH	2.5
*Hordeum bogdanii Wilensky*	0.756	0.927	LQHP	2.5
*Hordeum brevisubulatum* (Trin.) Lin*k*	0.710	0.902	LQHP	0.5
*Hordeum bulbosum* L.	0.770	0.936	LQH	2.0
*Hordeum californicum* Covas & Stebbins	0.789	0.927	H	2.5
*Hordeum chilense* Roem. & Schult.	0.714	0.830	LQHPT	2.5
*Hordeum marinum* Huds.	0.770	0.935	LQHP	1.0
*Hordeum murinum* L.	0.660	0.875	LQH	2.0
*Hordeum muticum* J.Presl	0.693	0.894	LQ	2.0
*Hordeum pusillum* Nutt.	0.821	0.944	LQHP	1.5
*Hordeum roshevitzii* Bowden	0.814	0.940	LQHPT	3.0
*Hordeum spontaneum* K. Koch	0.719	0.901	H	1.0
*Hordeum stenostachys* Godr.	0.821	0.904	LQHPT	2.5

RM, Regularization Multiplier; FC, Feature Combination; L, Linear; H, Hinge; Q, Quadratic; P, Product; T, Threshold.

### 3.2 Distribution pattern of potential habitat suitability for *Hordeum* under contemporary climate

**Figure 1:**
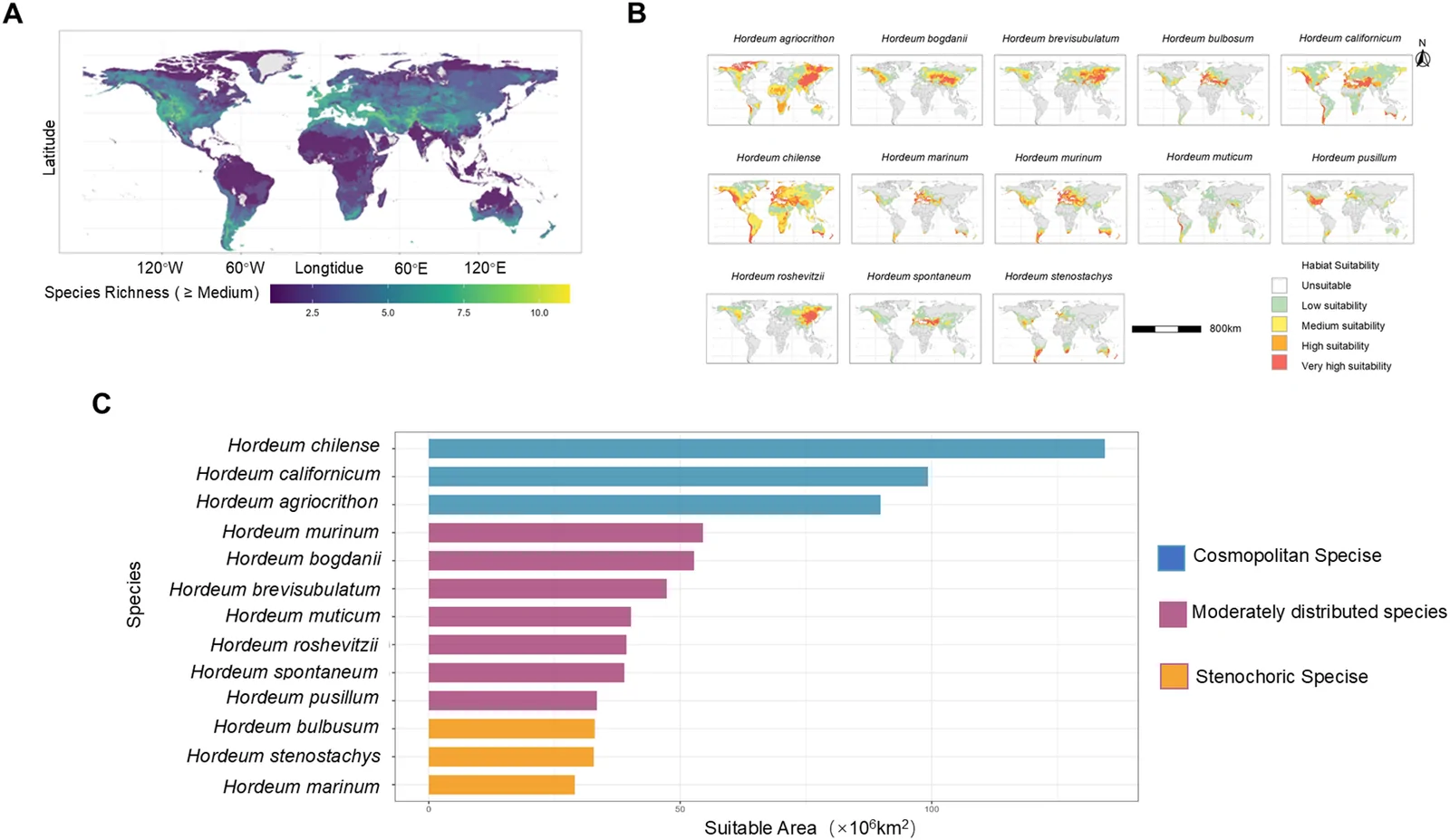
Global distribution of suitable habitats, species richness patterns, and comparison of distribution types of *Hordeum.* **(A)** Global distribution pattern of species richness of *Hordeum*. **(B)** Current predicted global distribution of suitable habitats for 13 *Hordeum* species. **(C)** Comparison of current (1970-2000) suitable habitat area and distribution type among different *Hordeum* species.

MaxEnt projections revealed marked biogeographic heterogeneity in the global distribution of *Hordeum* species (**Figures 1A, B**). Diversity hotspots, characterized by the highest species richness, were concentrated in four distinct geographic regions: (1) Southwest/Central Asia, the domestication cradle of cultivated barley and a key reservoir of wild barley genetic diversity; (2) the Mediterranean Basin, where coastal and insular zones support multiple annual taxa; (3) western North America, extending from the Rocky Mountains to the Pacific coastal zone, which acts as the continental distribution center for endemic native *Hordeum* species of North America; (4) the Andean-Patagonian region, a primary diversification hub for South American endemic taxa. Species richness in these hotspots reached 4–6 taxa, collectively delineating the global core distribution range of the genus. In sharp contrast, tropical regions, most parts of Africa, and high-latitude areas (including Antarctica and Greenland) are largely unsuitable for *Hordeum* colonization, exhibiting extensive low-richness zones or completely uninhabitable areas where only 1–2 widespread species occur sporadically.

Life-history strategies (annual vs. perennial) of *Hordeum* species were closely associated with their geographic distribution patterns and niche characteristics (**Figure 1B**). Perennial taxa (e.g., *H. bogdanii*, *H. brevisubulatum*) predominantly occupied continental climate zones characterized by pronounced seasonal fluctuations, particularly in interior Central Asian and the North American Great Plains. These regions experience high temperature seasonality and substantial environmental stress, favoring perennial adaptations such as nutrient storage via underground rhizomes and biomass allocation to structural stress tolerance—traits that facilitate persistence under harsh and variable conditions. In contrast, annual species (e.g., *H. murinum*, *H. spontaneum*) were highly restricted to Mediterranean-type climates, which are characterized by winter-spring moisture and summer drought. These annuals employ a stress-avoidance strategy: they rapidly complete their life cycles during the favorable wet season and produce drought-resistant seeds to survive the dry summer, representing a highly efficient adaptation to seasonal aridity.

Potential suitable area and niche breadth varied considerably among the 13 *Hordeum* species (**Figure 1C**). Based on the extent of predicted distribution, species were clearly delineated into two functional groups: (1) Broadly adapted generalists, represented by *H. chilense* (total suitable area: 134.2×10^6^ km^2^) and *H. californicum* (92.8×10^6^ km^2^), whose ranges spanned temperate zones across multiple continents, reflecting wide ecological niches and strong environmental tolerance; (2) Narrowly adapted specialists, represented by *H. stenostachys* (32.8×10^6^ km^2^) and *H. marinum* (29.1×10^6^ km^2^) (**Supplementary Table 5**). *H. stenostachys* is confined to montane environments in Central Asia, while *H. marinum* specializes in coastal saline habitats along the Mediterranean-Middle East-European corridor, indicating highly specialized ecological requirements for both.

**Figure 2:**
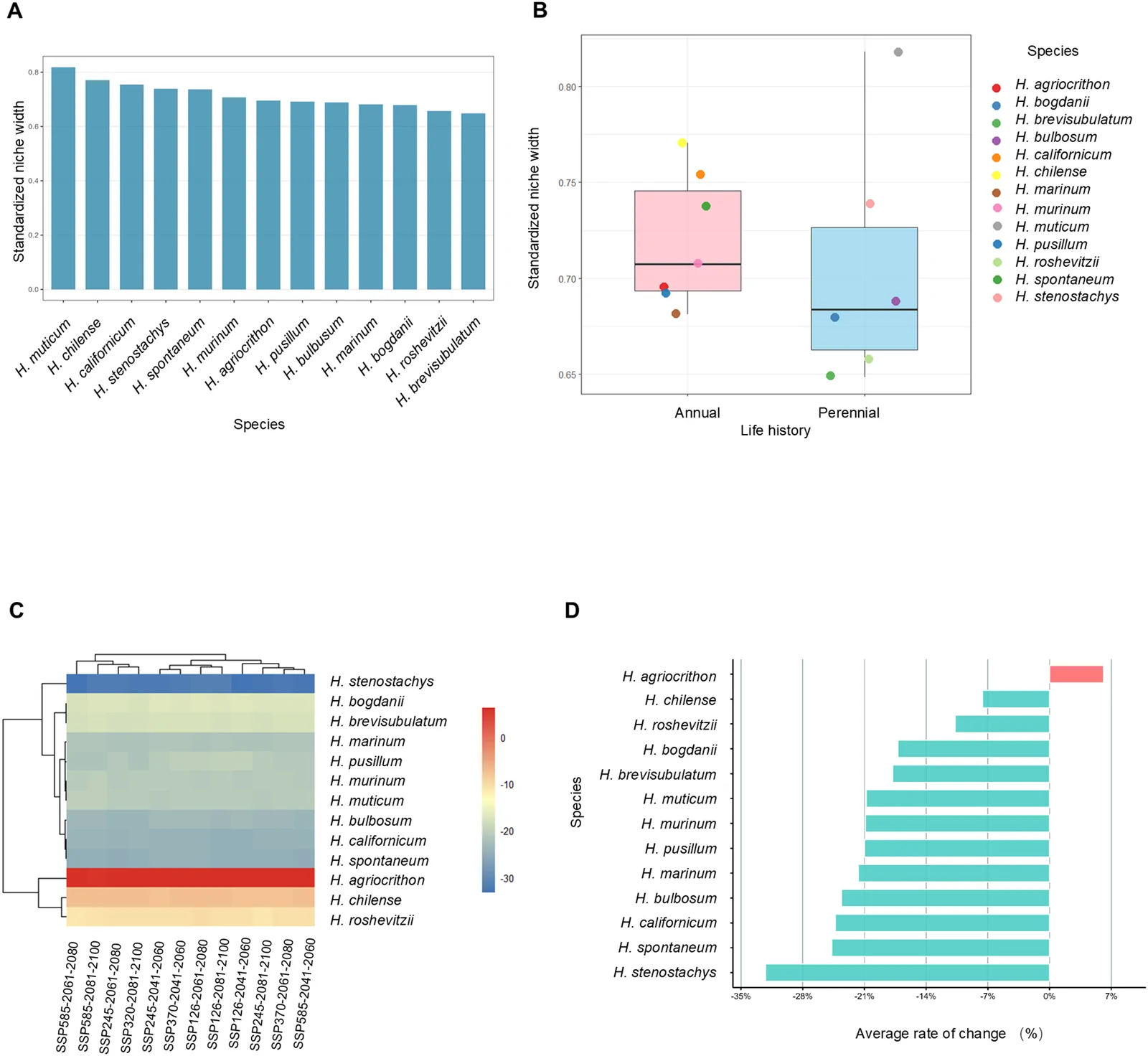
Multidimensional characteristics and climate change responses of niche breadth in *Hordeum* species. **(A)** Bar chart showing comparison of standardized niche breadth among different *Hordeum* species. **(B)** Boxplot illustrating niche breadth differences between annual (n = 7) and perennial (n = 6) *Hordeum* species (Wilcoxon rank-sum test, W = 29, p = 0.295, r = 0.317, medium effect size). **(C)** Heatmap of *Hordeum* species niche breadth under different SSP climate scenarios and periods. **(D)** Barplot depicting the average rate of change in niche breadth across *Hordeum* species.

Niche breadth analysis (**Figure 2A**) further clarified the underlying ecological strategies of these species. The perennial species *H. muticum* exhibited the widest niche (0.818), while *H. brevisubulatum* is the narrowest (0.648). Among annuals, the widespread *H. chilense* possessed a relatively wide niche (0.770), whereas the specialized *H. marinum* maintained a comparatively narrow niche (0.681) (**Supplementary Table 6**). Although annual species nominally exhibited wider niche breadths (median = 0.707, n = 7) than perennials (median = 0.684, n = 6), this difference did not reach statistical significance (two-tailed Wilcoxon rank-sum test, W = 29, p = 0.295, r = 0.317, medium effect size), likely due to limited sample size and high intragroup variability, notably the outlier *H. muticum* (0.818) in the perennial group (**Figure 2B**). Nevertheless, the observed trend aligns with the resource-exploitation strategy of annuals, characterized by rapid resource utilization, high reproductive allocation, and opportunistic growth across diverse seasonal environments, whereas perennials maintain narrower, climatically conservative niches tied to long-term local adaptation.

### 3.3 Projected dynamic impacts of future climate change on *Hordeum* habitat suitability

Niche breadth dynamics were highly consistent with the trends in suitable area (**Figures 2C, D**). *H. agriocrithon* was the only species showing niche expansion. All other species underwent niche contraction, with reduction rates ranging from -32.2% to -7.6%. The most severe niche contraction was again observed in *H. stenostachys* (-32.2%) and *H. spontaneum* (-24.7%), while *H. chilense* exhibited the mildest reduction (-7.6%). This convergence indicates that the future adaptive capacity of *Hordeum* species is primarily governed by species-specific ecological and functional traits rather than by current niche breadth alone.

**Figure 3:**
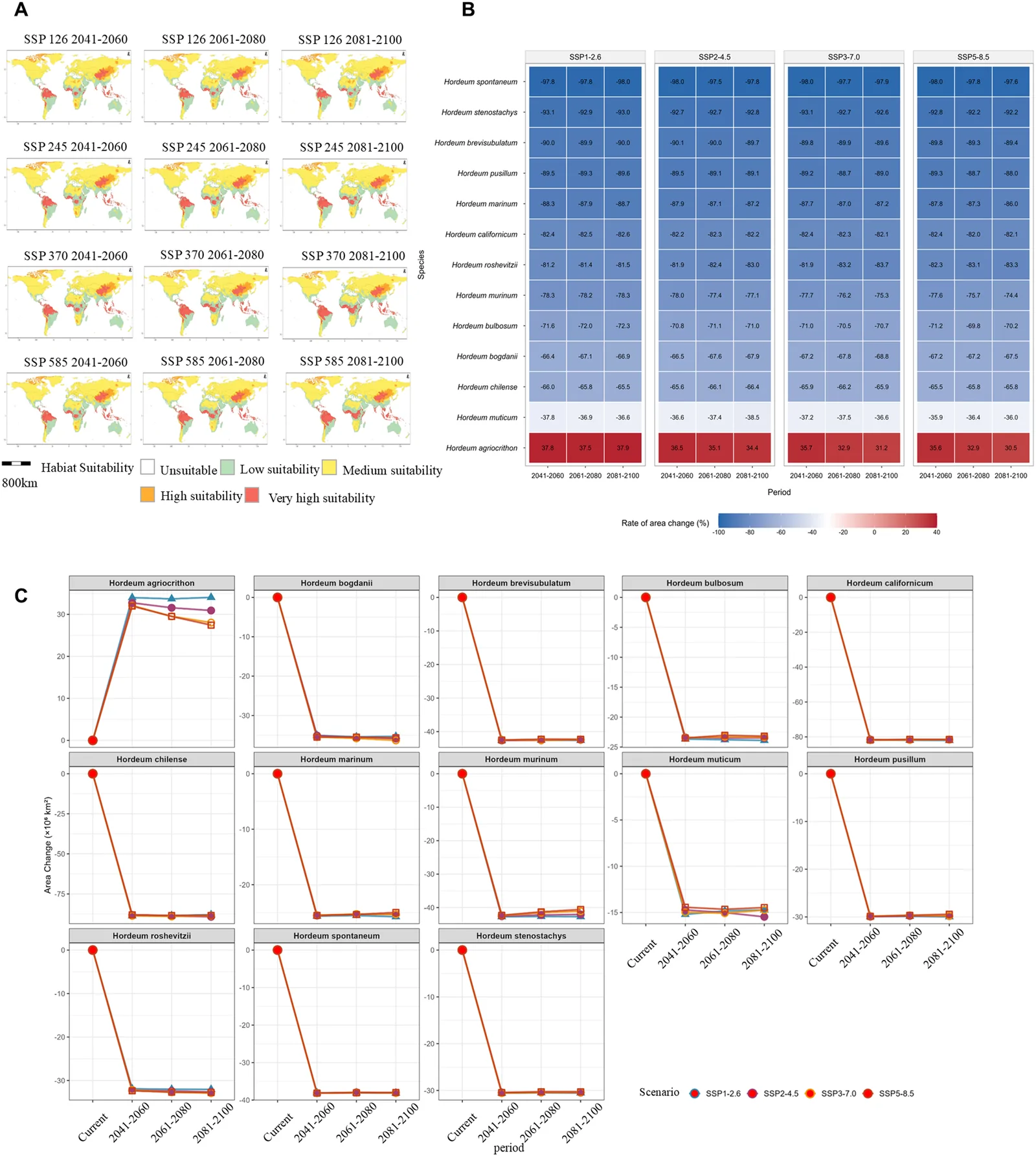
Dynamic predictions of potential suitable areas for *Hordeum* species under future climate scenarios. **(A)** Global spatial distribution of potential suitable habitats for *Hordeum agriocrithon* under multiple future climate scenarios. **(B)** Heatmap of the rate of change in suitable habitat area for 13 *Hordeum* species under future climate scenarios. **(C)** Temporal trends in suitable habitat area for 13 *Hordeum* species under multiple future climate scenarios.

Projections across all four SSP scenarios (SSP1-2.6, SSP2-4.5, SSP3-7.0, SSP5-8.5) indicate a severe and widespread contraction of Hordeum species’ ranges (**Supplementary Table 7**). By 2081–2100, the aggregated suitable habitat summed across all species is projected to drop by 62.3–62.96% compared with the 72.49×10^7^ km^2^ cumulative value under the 1970–2000 contemporary climate baseline (**Figure 3A**; **Supplementary Figures 5–17**). Note that this total incorporates overlapping suitable pixels of different species and is consequently larger than the global total land area. Notably, the contraction magnitude exhibited a nonlinear threshold response: mid-range emission scenarios (SSP2-4.5, SSP3-7.0) incurred the maximum losses (62.95% and 62.96%, respectively), while both low- (SSP1-2.6: 62.3%) and high-emission scenarios (SSP5-8.5: 62.62%) showed slightly attenuated contraction. This suggests the existence of a critical thermal threshold beyond which cumulative habitat loss saturates, with emission trajectories primarily modulating local-scale suitability patterns rather than the aggregated total habitat area across all species.

Despite this overall contraction trend, interspecific variation in climate change responses was substantial, highlighting the decisive role of species-specific biological traits. *H. agriocrithon* was the only species to exhibit consistent range expansion across all scenarios and future periods (**Figure 3B**), with its suitable area increasing by an average of 34.84% by the 2080s—demonstrating strong adaptive potential under warmer, drier conditions. The remaining 12 species all experienced range contraction, with the magnitude intensifying over time and with increasing emission intensity (**Supplementary Figure 5**). The highest-risk taxa included *H. spontaneum* (the progenitor of cultivated barley, with a mean contraction of 97.83%) and the narrow endemic *H. stenostachys* (mean contraction of 92.73%), both facing acute extinction risks. In contrast, the broadly adapted *H. chilense* (mean contraction of 65.87%) and the wide-niched perennial *H. muticum* (mean contraction of 36.96%) showed relatively modest declines, reflecting robust climatic buffering capacity (**Figure 3C**; **Table 3**). Notably, niche breadth and suitable area capture distinct dimensions of climate vulnerability: niche breadth reflects the width of a species’ realized climatic tolerance along environmental axes, whereas suitable area depends on the geographic availability of sites where those climatic conditions co-occur. Consequently, a species may retain a relatively stable climatic tolerance range (as reflected by minimal niche breadth contraction) while experiencing severe geographic range loss if the spatial distribution of suitable climate shifts or contracts under future climate scenarios—a phenomenon driven by the spatial displacement of climate envelopes rather than by erosion of the species’ intrinsic adaptive capacity.

**Table 3:** Projected habitat change and contemporary ecological metrics for 13 *Hordeum* species under the SSP5-8.5 scenario (2081–2100).

Species	Current area (×10^6^ km^2^)	Niche breadth	Habitat change (%)	Risk category
*H. agriocrithon*	89.83	0.696	+34.84	Expander
*H. spontaneum*	38.90	0.737	−97.83	Extreme risk
*H. stenostachys*	32.83	0.739	−92.73	Extreme risk
*H. brevisubulatum*	47.32	0.648	−89.79	Severe risk
*H. pusillum*	33.44	0.692	−89.08	Severe risk
*H. marinum*	29.02	0.681	−87.51	Severe risk
*H. roshevitzii*	39.30	0.657	−82.41	Severe risk
*H. californicum*	99.28	0.754	−82.29	Severe risk
*H. murinum*	54.54	0.707	−77.01	High risk
*H. bogdanii*	52.78	0.679	−67.36	Moderate risk
*H. chilense*	134.42	0.770	−65.87	Moderate risk
*H. muticum*	40.21	0.818	−36.96	Low risk
*H. bulbosum*	33.02	0.690	−71.02	High risk

Negative values indicate habitat contraction; positive values indicate range expansion. Risk categories are defined by habitat change magnitude: Expander (>0%), Low risk (>−40%), Moderate risk (−40% to −70%), High risk (−70% to −80%), Severe risk (−80% to −90%), Extreme risk (<−90%). Niche breadth reflects the width of a species’ realized climatic tolerance along environmental axes. Full multi-scenario and multi-period results are provided in **Supplementary Table 7**.

### 3.4 Niche dynamics and spatial response of *Hordeum* under future climate change

**Figure 4:**
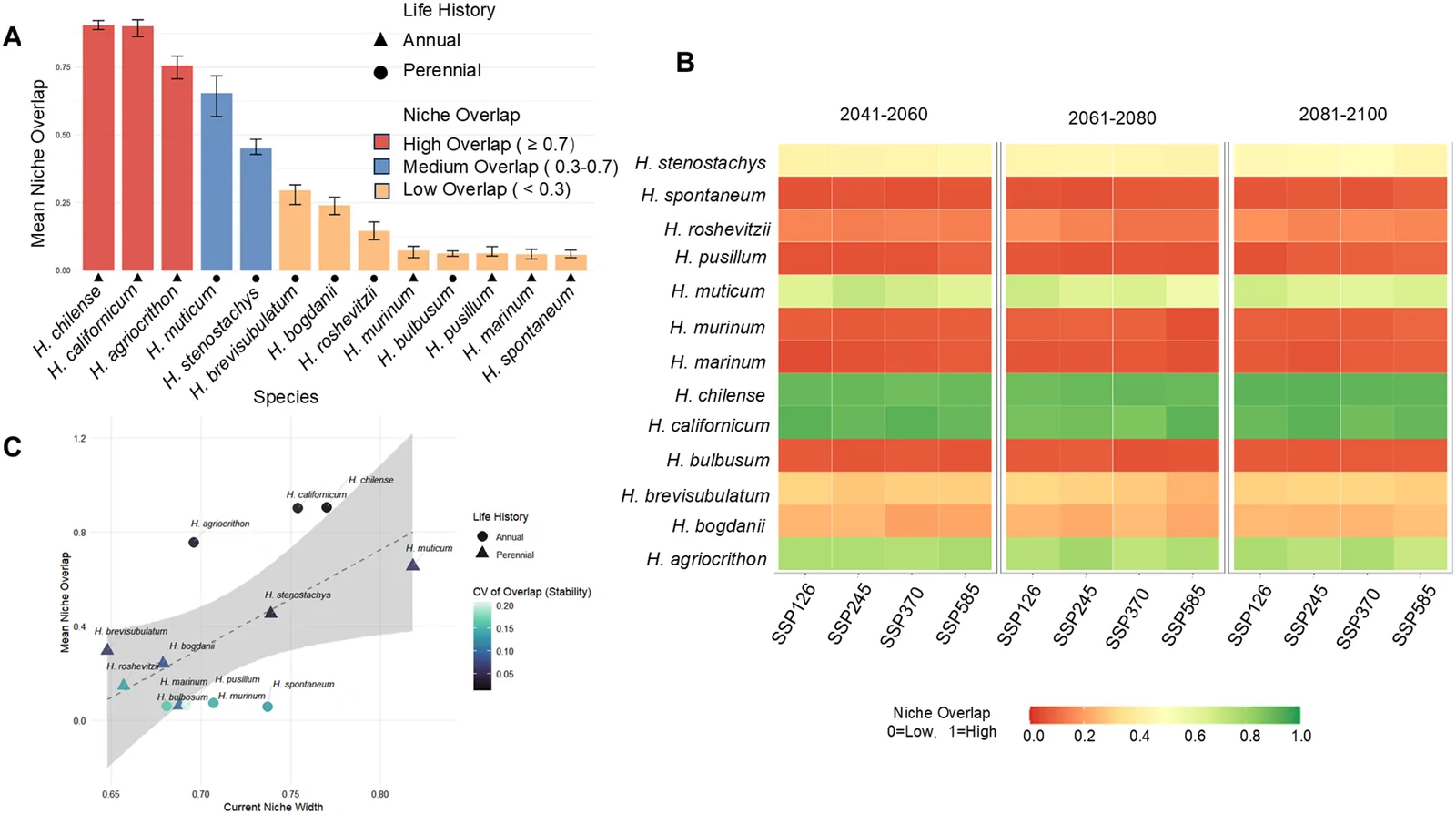
Pattern characteristics, climate change responses, and associations with Niche breadth of niche overlap in *Hordeum* species. **(A)** Bar chart comparing mean niche overlap among different *Hordeum* species, categorized by life history and overlap level. **(B)** Heatmap of *Hordeum* species niche overlap under different SSP climate scenarios and time periods. **(C)** Scatter plot showing the association between current niche breadth and mean niche overlap in *Hordeum* species, with uncertainty envelope (Pearson correlation: r = 0.605, R^2^ = 0.366, p = 0.029; Spearman correlation: ρ = 0.465, p = 0.109).

Niche overlap among *Hordeum* species exhibited a distinct three-tiered differentiation pattern (**Figure 4A**; **Supplementary Tables 8**, **S9**). The high-overlap type (≥ 0.7) comprised three annual species—*H. californicum*, *H. chilense*, and *H. agriocrithon*—with a mean pairwise overlap of 0.756–0.905 and a low coefficient of variation (CV = 0.012–0.031). This indicates highly similar ecological requirements, potential intense resource competition, and remarkably stable niche relationships among these taxa. The intermediate type (0.3–0.7) was dominated by perennial species such as *H. muticum* and *H. stenostachys*, with a mean overlap of 0.241–0.654 and moderately higher CV values (0.035–0.082). The low-overlap type (< 0.3) included six species with highly specialized niches (mean overlap: 0.058–0.146) and significant instability (CV: 0.101–0.206), suggesting vulnerability to environmental fluctuations.

The three-tiered niche overlap structure maintained remarkable stability across all climate scenarios and time horizons (**Figure 4B**). Even under the most extreme SSP5-8.5 scenario, the difference in mean niche overlap between the far future (2081–2100) and the near future (2041–2060) was less than 5%. This persistent structure implies that niche differentiation among *Hordeum* species is driven by deep-seated genetic and biological constraints rather than short-term phenotypic plasticity. Correlation analysis revealed a moderate positive trend between current niche breadth and mean niche overlap across the 13 species (Spearman ρ = 0.465), though statistical significance was not achieved (p = 0.109), likely reflecting limited sample size and non-normal distribution of overlap data (Shapiro-Wilk W = 0.822, p = 0.013). For comparison, Pearson correlation yielded a nominally significant result (r = 0.605, p = 0.029, R^2^ = 0.366), which should be interpreted cautiously given the violation of bivariate normality assumption (**Figure 4C**). Despite these exceptions—notably *H. spontaneum* and *H. murinum*, which exhibited moderate niche breadths (0.737 and 0.707) but exceptionally low overlap (0.058 and 0.074), likely due to geographic isolation and competitive exclusion—the overall pattern supports the view that annual species tend to occupy the high-breadth/high-overlap quadrant, while perennial species predominantly fall within the medium-low breadth/medium-low overlap domain.

**Figure 5:**
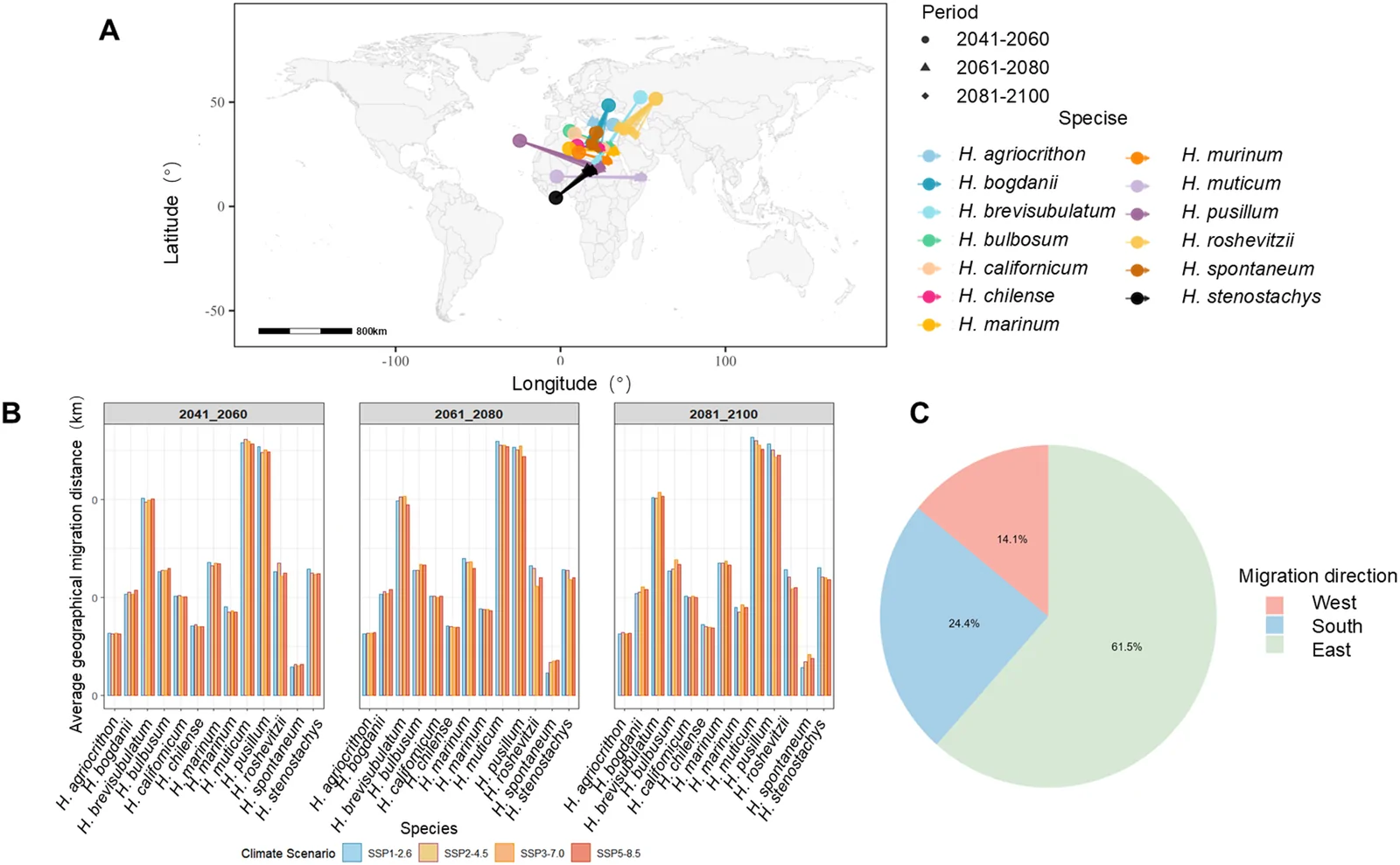
Geographic migration characteristics of Hordeum species under climate change scenarios. **(A)** Geographic migration pathways of Hordeum species under current and future climate scenarios, presented on a world map excluding the Antarctic region (latitude < -60°). Solid red circles denote each species’ current niche centroid, while colored lines with arrows—where colors correspond to individual species—connect these current positions to future centroids, indicating the direction of climate-driven shifts; future centroids are represented by colored points of distinct shapes (circle = 2041–2060, triangle = 2061–2080, diamond = 2081–2100) that match their species’ color coding, with a right-hand legend providing clear reference for period shapes and species colors to differentiate migration directions and distances across 13 Hordeum species and future climate scenarios; **(B)** Bar charts showing average migration distances of Hordeum species across different time periods and SSP climate scenarios; **(C)** Pie chart illustrating the proportional distribution of overall migration directions in Hordeum species.

Niche centroid displacement patterns exhibited pronounced species specificity under future climate change (**Figure 5A**; **Supplementary Table 10**). Eastward shifts predominated, accounting for 61.5% of the species (n=8), followed by southward (24.4%, n=3) and westward (14.1%, n=2) shifts (**Figure 5A**); most species maintained consistent directional trajectories of suitable habitat shift across scenarios (**Supplementary Figures 18–27**). Spatially, centroid displacement distances varied dramatically among species, ranging from <1,000 km in *H. spontaneum* to >3,000 km in *H. muticum*, *H. pusillum*, and *H. brevisubulatum*, with the remaining species showing intermediate displacements (1,000–3,000 km). Importantly, displacement distances were positively correlated with emission scenario intensity (**Figures 5B, C**).

These spatial responses were closely associated with the species’ drought tolerance strategies, functionally categorized based on precipitation of the driest month (bio14). Drought-tolerant taxa (e.g., *H. stenostachys*) exhibited the smallest niche centroid displacements, indicating strong *in situ* persistence capacity. In contrast, hydrophilic species (e.g., *H. brevisubulatum*) showed the longest displacements, reflecting heightened vulnerability to future aridification. Across all species, niche centroid displacements consistently tracked environmental gradients toward higher precipitation or reduced water stress, typically shifting to higher latitudes or areas influenced by specific moisture corridors. This universal pattern reflects a fundamental climatic niche-tracking strategy of suitable habitat employed by *Hordeum* species in response to ongoing climate change.

## 4 Discussion

### 4.1 Reliability of the methodology and model performance

The reliability of inferences drawn in this study hinges on the predictive accuracy of species distribution models. Systematic parameter optimization via the ENMeval package effectively mitigated overfitting risks associated with default MaxEnt settings, substantially enhancing both predictive performance and model transferability across different climatic contexts (Zhang et al., 2025). All 13 *Hordeum* species achieved a mean AUC of 0.907 and a mean TSS of 0.763, values consistent with performance benchmarks in high-quality distribution modeling studies (Shi et al., 2023; He et al., 2025), confirming the robustness of our predictions. However, these metrics reflect discriminatory ability rather than ecological realism; because biotic interactions, dispersal constraints, and anthropogenic factors were not incorporated, predictions indicate climatic suitability rather than realized distributions. Despite these limitations, these rigorously calibrated models provide an empirical foundation for subsequent analyses of *Hordeum* range dynamics, niche differentiation, and conservation prioritization.

### 4.2 The interplay of three determinants shaping contemporary *Hordeum* distribution

Historical, environmental and biological factors jointly govern the present distribution of *Hordeum*, with four major diversity hotspots (Southwest-Central Asia, the Mediterranean Basin, western North America and the Andean-Patagonian region) consistent with global diversity centers of temperate plants (Barberá et al., 2025). Previous studies indicate that Quaternary glacial cycles induced population isolation, range contraction and post-glacial expansion, which may have triggered allopatric speciation and adaptive divergence within *Hordeum* lineages (Xiong et al., 2025). Consistent with this interpretation, cumulative historical effects have turned Mediterranean and Central Asian Pleistocene refugia into core diversity hubs.

Environmental filters and habitat heterogeneity directly regulate species distribution and coexistence. Complex topographies create microhabitat differentiation and facilitate niche partitioning (Xu et al., 2025; Yang et al., 2025). Temperate *Hordeum* cannot endure hot and humid tropical climates, while extreme aridity restricts the formation of multi-species communities (Samat et al., 2025). Biological traits are tightly coupled with external environments. Perennial *Hordeum* evolve stress-tolerant strategies to adapt to continental climates, whereas annual taxa adopt drought-avoidance tactics suited to seasonal Mediterranean climates (Kelly et al., 2021; Poppenwimer et al., 2023). This life-history split shapes modern geographic patterns and predicts divergent climate responses. Long-term local adaptation and stable resource reliance force perennials to maintain narrow, climatically conservative niches with weak environmental plasticity (Boyko et al., 2023; Delalandre, 2024). In contrast, annual barley develops short life cycles, flexible reproduction and strong dispersal capacity to occupy variable habitats. The wider niche breadths of annual species stem from their opportunistic traits, highlighting the central role of biological characteristics in structuring species distributions across heterogeneous landscapes (Chen et al., 2025).

**Figure 6:**
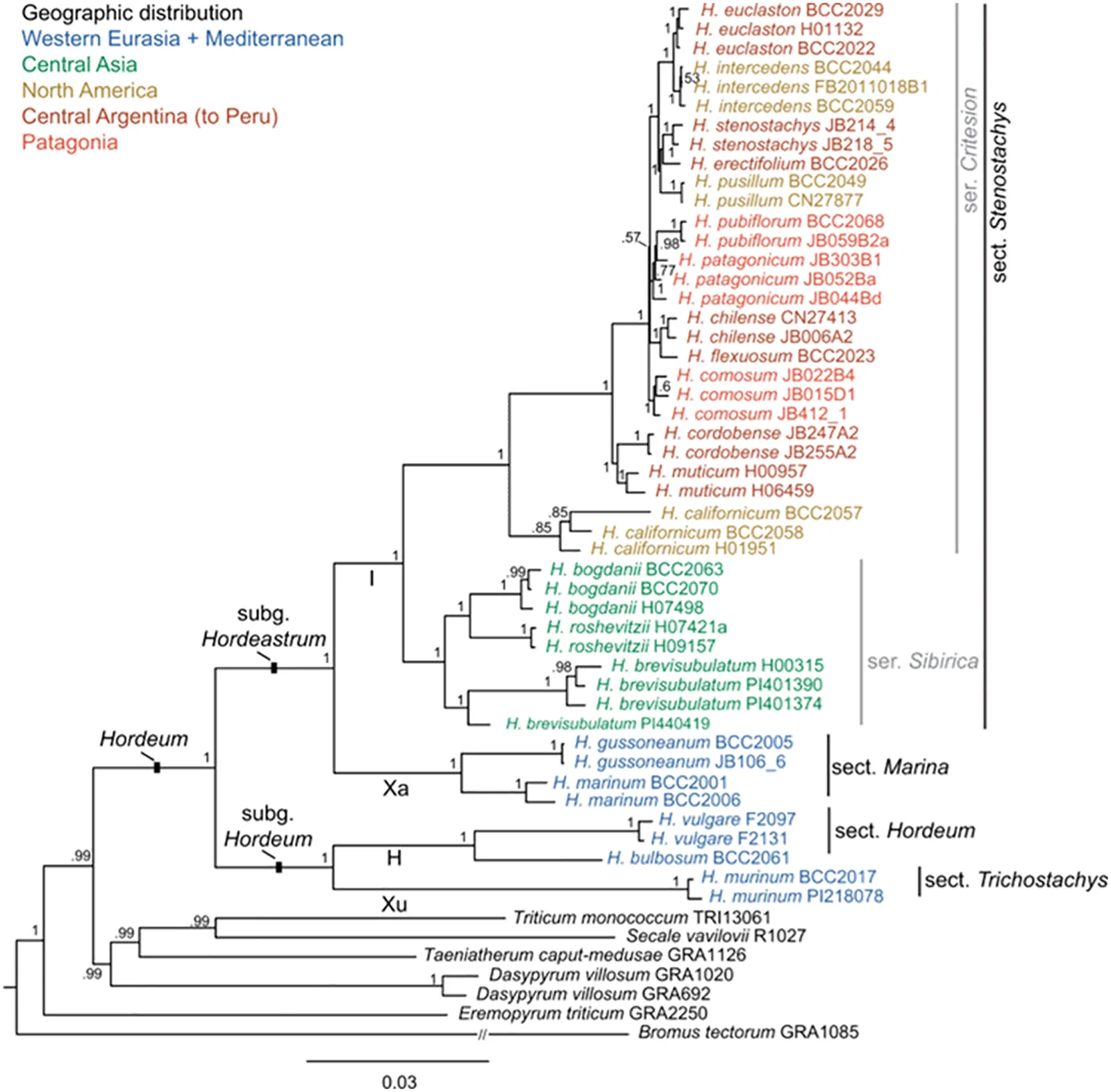
Phylogenetic tree derived from the concatenated supermatrix consisting of 13 loci of the diploid *Hordeum* taxa and six out-group species calculated with Bayesian inference. Posterior probability values of the clades are indicated close to the nodes. Infrageneric treatment of the genus and genome denominations (bold letters along branches) in *Hordeum* follow (Brassac and Blattner, 2015).

Our MaxEnt-based niche modelling results match the multilocus coalescent phylogeny of the genus established by Brassac & Blattner (Brassac and Blattner, 2015) (**Figure 6**). The phylogenetic tree subdivides *Hordeum* into four genetically distinct monophyletic clades whose topology spatially corresponds to the four diversity hotspots identified in this study: the Central Asian–Siberian clade covers the Eurasian continental core, the Mediterranean H/Xu/Xa clades correspond to the circum-Mediterranean basin, the North American *Critesion* clade spans western United States coastal zones, and the South American *Stenostachys* clade is restricted to the Andean-Patagonian zone. Such spatial consistency supports the interpretation that long-term allopatric isolation has served as the primary historical driver of modern macro-scale *Hordeum* distributions.

Deep phylogenetic separation between annual and perennial lineages provides a plausible evolutionary explanation for the niche breadth differences detected in our analyses. All perennial species examined belong to the early-diverging Siberian basal clade, including *H. bogdanii*, *H. brevisubulatum* and *H. roshevitzii*, while nearly all terminal branches are composed of annual taxa. Notably, *H. muticum* represents a rare perennial within New World derived lineages, with a standardized niche breadth of 0.818. This feature may originate from secondary perennial adaptation after lineage differentiation. This ancient life-history dichotomy produces two distinct adaptive modes: annual barley evolves broader climatic niches and drought-avoidance strategies, while perennials retain narrow, climate-stable niches, leading to systematic disparities in climate vulnerability.

All New World *Hordeum* fall on terminal phylogenetic branches and form a recently diverged monophyletic group, among which the *stenostachys* clade is endemic to Patagonia (Blattner, 2009). This lineage has a short evolutionary timeline and limited accumulated genetic variation, resulting in narrow environmental tolerance and explaining the severe range shrinkage of Patagonian narrow endemics predicted by our models. Simulations show the suitable habitat of the diploid endemic *H. stenostachys* decreases by 89.4%, and the wild progenitor of cultivated barley *H. spontaneum* loses up to 97.83% of its suitable areas; both are narrow endemic diploids with simple genetic backgrounds. Furthermore, repeated independent allopolyploidization events occurred across the four H, Xu, Xa and I genomic lineages, involving at least six extinct diploid ancestors during polyploid formation. The genetic buffering effect brought by combined multiple genomes improves climatic resistance. Our projections show polyploids only suffer a 36.96%–57.82% range reduction, far less than diploid endemics with over 89% habitat loss, demonstrating that genomic composition is a key factor regulating species responses to climate change.

Collectively, divergent climate responses among wild barley likely reflect evolutionary outcomes shaped by deep phylogenetic history, life-history strategies and genomic architecture, rather than being mainly determined by short-term phenotypic plasticity. Such phylogenetically structured vulnerability indicates that evolutionary lineage background must be fully considered when prioritizing germplasm conservation and breeding climate-resilient barley varieties.

### 4.3 Commonalities and specificities in future climate change responses

The projected ~62% contraction of total *Hordeum* suitable range under the BCC-CSM2-MR model aligns with findings for crop wild relatives (CWRs) (Ziska, 2021; Boyko et al., 2023), highlighting the systematic vulnerability of temperate Poaceae CWRs to global warming. Severe loss of high-suitability habitats will cause core population collapse and genetic erosion, and low-emission pathways (SSP1-2.6) can significantly mitigate these impacts (De Oliveira Aparecido et al., 2025). Interspecific differences in contraction magnitude reflect deterministic biological constraints: *H. agriocrithon* is the only expanding taxon, with unique genomic characteristics conferring strong climatic adaptability (Wang et al., 2023; Zhou et al., 2024; Kaňuková et al., 2025), making it a valuable genetic resource for breeding climate-resilient barley.

Extreme contraction (>90% habitat loss) characterizes *H. spontaneum* and *H. stenostachys*, likely driven by their narrow climatic envelopes and limited adaptive capacity. Severe contraction (80%–90% loss) affects *H. brevisubulatum*, *H. pusillum*, *H. marinum*, *H. californicum*, and *H. roshevitzii*. Notably, *H. murinum* (~74%–78% loss), despite its subspecific genetic differentiation across a broad geographic range, exemplifies the “ubiquity paradox” (Chano et al., 2021), where ecological generalism fails to buffer against rapid climate change. *H. chilense* (~65%–66% loss), the most widespread species with the broadest standardized niche width (**Figure 2A**), shows relatively moderate contraction, suggesting that broad climatic tolerance may confer partial resilience. The mildest decline is observed in *H. muticum* (~36%–38% loss), whose intermediate distribution range and stable niche structure may underlie its relative stability across emission pathways.

The contrasting fates of species across the range-size spectrum challenge simplistic predictions: the severe contraction of intermediate-ranging *H. roshevitzii* (-81% to -84%) and narrow endemic *H. marinum* (-86% to -89%) demonstrates that neither broad distribution nor specialized niche adaptation guarantees climatic resilience, particularly when environmental shifts exceed physiological tolerances. This pattern cautions against uncritical application of the “edge adaptation hypothesis” to CWR conservation planning without species-specific vulnerability assessment (Ovesna et al., 2023).

Furthermore, the decoupling between niche breadth stability and geographic range collapse observed in *H. chilense* exemplifies climate-space displacement—a phenomenon where the geographic footprint of a stable climatic envelope shifts or contracts due to altered climate patterns, rather than reflecting erosion of intrinsic adaptive capacity (Wohlgemuth, 2018). This underscores that conservation planning must account for both species’ intrinsic adaptive capacity and the spatial dynamics of their climatic niches (Sexton et al., 2017).

Additionally, the stability of interspecific niche overlap structures indicates that the fundamental niche of *Hordeum* is a phylogenetically conserved trait (Gagnon et al., 2023; Wu S. et al., 2024), challenging the assumption that “rapid niche evolution rescues species”. Collectively, these findings demonstrate that future adaptive capacity in CWRs is not determined by current niche breadth per se, but rather by the interplay between intrinsic physiological tolerance and the spatial rearrangement of climate envelopes—a critical distinction for predicting species-specific responses under accelerating global warming.

It should be emphasized that these projected range shifts represent potential trajectories under the BCC-CSM2-MR model and the CMIP6 SSP scenarios, which embody inherently uncertain socioeconomic trajectories. The use of a single Earth System Model does not capture the full inter-model variability in climate sensitivity and regional patterns that multi-model ensembles would provide. Consequently, the exact magnitudes of habitat contraction may vary under alternative climate models; nevertheless, the directional trends—marked range contraction under high-emission pathways and interspecific divergence in vulnerability—are robust across the scenarios examined and informative for conservation planning.

### 4.4 Spatial adaptation strategies: migration as a “chasing climate” response

*Hordeum* species migrate to track suitable climates and escape increasingly unsuitable habitats, with both migration direction and magnitude positively correlated with the intensity of climatic stress. The consistent pattern of migration toward polar regions and higher precipitation areas confirms the universality of climate-driven distribution dynamics in temperate plants (Ziska, 2021). Differences in migration distances among species are driven by moisture sensitivity: hydrophilic taxa (e.g., *H. brevisubulatum*) require long-distance migration, dependent on dispersal capacity and habitat connectivity; drought-tolerant taxa (e.g., *H. stenostachys*) show minimal displacement and are capable of persisting *in situ* (Shubhi Sharma et al., 2022). The exceptionally long migration distances observed in polyploid species such as *H. brevisubulatum* may leverage genomic heterozygosity to cope with environmental uncertainty (Naghiloo and Vamosi, 2023; Feng et al., 2025). Nevertheless, for most species, projected migration requirements exceed realized dispersal rates, leading to obvious migration lag. To contextualize the magnitude of migration lag, our models project that species such as *H. brevisubulatum* require northward migration exceeding 3000 km by 2100 to track suitable climates (**Supplementary Table 10**). In contrast, documented natural dispersal rates for temperate grasses (Poaceae) typically range from 0.1 to 2 km per year (Nathan, 2006; Cain et al., 1998; Higgins and Cain, 2002). Under an optimistic scenario (2 km/year), a 3000-km range shift would require ~1500 years—15 times longer than the projected 100-year timeframe. Even for species with relatively high dispersal capacity, realized migration will likely achieve only 5–10% of the required range adjustment. This stark discrepancy confirms that natural dispersal capacity is grossly insufficient to keep pace with rapid climate alteration, which intensifies habitat loss, population decline and long-term extinction risk under continuous global change (Román-Palacios and Wiens, 2020; Zani et al., 2023).

### 4.5 Research limitations and future perspectives

This study has inherent limitations. First, models only incorporate climatic variables and did not account for soil properties, fine-scale topography, land-use change, biotic interactions, or CO_2_ fertilization effects; all of which may influence realized species distributions. Land-use change and CO_2_ fertilization are particularly relevant given ongoing agricultural expansion and altered competitive dynamics. Second, the assumption of unlimited dispersal capacity may overestimate future migration potential. Our quantitative comparison between projected climate-tracking distances (>3000 km over 100 years) and empirically documented grass dispersal rates (0.1–2 km/year) suggests that realized migration will likely achieve only 5–10% of the required range adjustment, even for species with relatively high dispersal capacity. Third, occurrence datasets may retain residual sampling bias, and phylogenetic relationships were not incorporated. Fourth, future projections rely on a single climate model; multi-model ensembles would better quantify uncertainty. Fifth, the exclusion of intraspecific genetic variation limits generalizability. Future research should integrate mechanistic dispersal models, account for land-use dynamics and biotic interactions, incorporate multi-model ensembles, and validate predictions through field observations. Interdisciplinary approaches will deepen understanding of *Hordeum* dynamics and inform targeted conservation strategies for this genus and other temperate crop wild relatives.

## 5 Conclusions

By integrating MaxEnt niche modeling with CMIP6 climate scenario analyses, this study systematically delineates the global distribution patterns and future adaptive trajectories of 13 *Hordeum* species. Our findings suggest that Quaternary evolutionary history and species-specific life-history strategies synergistically shape the contemporary diversity hotspots of the genus, providing a plausible theoretical foundation for understanding its biogeographic patterns. Under high-emission climate scenarios, *Hordeum* is projected to experience an aggregate range contraction exceeding 60%, with substantial divergent responses among species. Notably, *H. spontaneum*—a primary progenitor of cultivated barley—may face elevated future extinction risk, highlighting the potential importance of strengthened *in-situ* habitat protection and standardized *ex-situ* germplasm preservation to safeguard the precious wild barley resources. In contrast, *H. agriocrithon* shows considerable potential for range expansion, which may render it a promising genetic resource for future climate-resilient barley breeding programs. Critically, low-emission pathways (SSP1-2.6) substantially mitigate these climate-driven threats, demonstrating that proactive climate change mitigation could be pivotal for safeguarding *Hordeum* genetic resources and supporting sustained global food under ongoing climate change.

## Data Availability

The datasets presented in this study can be found in online repositories. The names of the repository/repositories and accession number(s) can be found in the article/**Supplementary Material**.
