# Supplementary material for: Climate change reshapes the global distribution of wild barley relatives: integrating life-history traits and ecological niche modeling
Source: Front Plant Sci. 2026 Aug 25;17:1935270. doi: 10.3389/fpls.2026.1935270 (PMC13550181; doi:10.3389/fpls.2026.1935270)
Supplement: Supplementary file 1 [file Table1.docx]

Supplementary Material

# 1 Supplementary Figures and Tables

## 1.1 Supplementary Figures

**Supplementary Figure 1.** Global geographic distribution patterns of 13 *Hordeum* species. Distribution points of each species are marked in red. The scale bar represents an actual distance of 800 km, and each panel shows the geographic distribution range of a single species.

Supplementary Figure 2. Pearson correlation coefficient heatmap of environmental variables. The heatmap displays pairwise Pearson correlation coefficients between different environmental variables (bio1–bio19, elevation). The color gradient represents the strength and direction of correlations, with red indicating positive correlations and blue indicating negative correlations.

**Supplementary Figure 3.** Principal Component Analysis (PCA) results of environmental variables for niche comparison analysis. A, Loading plot of environmental variables in principal component analysis. B, Scree plot showing the percentage of variance explained by each principal component.

**Supplementary Figure 4.** Performance evaluation and optimal parameter combination screening of MaxEnt niche models for *Hordeum* species. A, Receiver operating characteristic (ROC) curves showing the AUC values of the optimal MaxEnt models for each *Hordeum* species. B, Parameter response curves for identifying the optimal model parameter combinations in MaxEnt modeling of *Hordeum* species.

**Supplementary Figure 5.** Predicted potential suitable habitat distributions of *Hordeum agriocrithon* under future climate scenarios. Each panel presents the spatial distribution of potential suitable habitats for *Hordeum agriocrithon* under four shared socioeconomic pathway (SSP) climate scenarios (SSP1-2.6, SSP2-4.5, SSP3-7.0, SSP5-8.5) across three future time periods (2041–2060, 2061–2080, 2081–2100). Habitat suitability is categorized into five levels: unsuitable, low suitability, medium suitability, high suitability, and very high suitability, with a scale bar indicating an actual distance of 800 km.

**Supplementary Figure 6.** Predicted potential suitable habitat distributions of *Hordeum bogdanii* under future climate scenarios. Each panel presents the spatial distribution of potential suitable habitats for *Hordeum bogdanii* under four shared socioeconomic pathway (SSP) climate scenarios (SSP1-2.6, SSP2-4.5, SSP3-7.0, SSP5-8.5) across three future time periods (2041–2060, 2061–2080, 2081–2100). Habitat suitability is categorized into five levels: unsuitable, low suitability, medium suitability, high suitability, and very high suitability, with a scale bar indicating an actual distance of 800 km.

Supplementary Figure 7. Predicted potential suitable habitat distributions of *Hordeum* *brevisubulatum* under future climate scenarios. Each panel presents the spatial distribution of potential suitable habitats for *Hordeum* *brevisubulatum* under four shared socioeconomic pathway (SSP) climate scenarios (SSP1-2.6, SSP2-4.5, SSP3-7.0, SSP5-8.5) across three future time periods (2041–2060, 2061–2080, 2081–2100). Habitat suitability is categorized into five levels: unsuitable, low suitability, medium suitability, high suitability, and very high suitability, with a scale bar indicating an actual distance of 800 km.

Supplementary Figure 8. Predicted potential suitable habitat distributions of *Hordeum bulbosum* under future climate scenarios. Each panel presents the spatial distribution of potential suitable habitats for *Hordeum bulbosum* under four shared socioeconomic pathway (SSP) climate scenarios (SSP1-2.6, SSP2-4.5, SSP3-7.0, SSP5-8.5) across three future time periods (2041–2060, 2061–2080, 2081–2100). Habitat suitability is categorized into five levels: unsuitable, low suitability, medium suitability, high suitability, and very high suitability, with a scale bar indicating an actual distance of 800 km.

Supplementary Figure 9. Predicted potential suitable habitat distributions of *Hordeum californicum* under future climate scenarios. Each panel presents the spatial distribution of potential suitable habitats for *Hordeum californicum* under four shared socioeconomic pathway (SSP) climate scenarios (SSP1-2.6, SSP2-4.5, SSP3-7.0, SSP5-8.5) across three future time periods (2041–2060, 2061–2080, 2081–2100). Habitat suitability is categorized into five levels: unsuitable, low suitability, medium suitability, high suitability, and very high suitability, with a scale bar indicating an actual distance of 800 km.

**Supplementary Figure 10.** Predicted potential suitable habitat distributions of *Hordeum chilense* under future climate scenarios. Each panel presents the spatial distribution of potential suitable habitats for *Hordeum chilense* under four shared socioeconomic pathway (SSP) climate scenarios (SSP1-2.6, SSP2-4.5, SSP3-7.0, SSP5-8.5) across three future time periods (2041–2060, 2061–2080, 2081–2100). Habitat suitability is categorized into five levels: unsuitable, low suitability, medium suitability, high suitability, and very high suitability, with a scale bar indicating an actual distance of 800 km.

**Supplementary Figure 11.** Predicted potential suitable habitat distributions of *Hordeum marinum* under future climate scenarios. Each panel presents the spatial distribution of potential suitable habitats for *Hordeum marinum* under four shared socioeconomic pathway (SSP) climate scenarios (SSP1-2.6, SSP2-4.5, SSP3-7.0, SSP5-8.5) across three future time periods (2041–2060, 2061–2080, 2081–2100). Habitat suitability is categorized into five levels: unsuitable, low suitability, medium suitability, high suitability, and very high suitability, with a scale bar indicating an actual distance of 800 km.

**Supplementary Figure 12.** Predicted potential suitable habitat distributions of *Hordeum murinum* under future climate scenarios. Each panel presents the spatial distribution of potential suitable habitats for *Hordeum murinum* under four shared socioeconomic pathway (SSP) climate scenarios (SSP1-2.6, SSP2-4.5, SSP3-7.0, SSP5-8.5) across three future time periods (2041–2060, 2061–2080, 2081–2100). Habitat suitability is categorized into five levels: unsuitable, low suitability, medium suitability, high suitability, and very high suitability, with a scale bar indicating an actual distance of 800 km.

**Supplementary Figure 13.** Predicted potential suitable habitat distributions of *Hordeum muticum* under future climate scenarios. Each panel presents the spatial distribution of potential suitable habitats for *Hordeum muticum* under four shared socioeconomic pathway (SSP) climate scenarios (SSP1-2.6, SSP2-4.5, SSP3-7.0, SSP5-8.5) across three future time periods (2041–2060, 2061–2080, 2081–2100). Habitat suitability is categorized into five levels: unsuitable, low suitability, medium suitability, high suitability, and very high suitability, with a scale bar indicating an actual distance of 800 km.

**Supplementary Figure 14.** Predicted potential suitable habitat distributions of *Hordeum pusillum* under future climate scenarios. Each panel presents the spatial distribution of potential suitable habitats for *Hordeum pusillum* under four shared socioeconomic pathway (SSP) climate scenarios (SSP1-2.6, SSP2-4.5, SSP3-7.0, SSP5-8.5) across three future time periods (2041–2060, 2061–2080, 2081–2100). Habitat suitability is categorized into five levels: unsuitable, low suitability, medium suitability, high suitability, and very high suitability, with a scale bar indicating an actual distance of 800 km.

**Supplementary Figure 15.** Predicted potential suitable habitat distributions of *Hordeum roshevitzii* under future climate scenarios. Each panel presents the spatial distribution of potential suitable habitats for *Hordeum roshevitzii* under four shared socioeconomic pathway (SSP) climate scenarios (SSP1-2.6, SSP2-4.5, SSP3-7.0, SSP5-8.5) across three future time periods (2041–2060, 2061–2080, 2081–2100). Habitat suitability is categorized into five levels: unsuitable, low suitability, medium suitability, high suitability, and very high suitability, with a scale bar indicating an actual distance of 800 km.

**Supplementary Figure 16.** Predicted potential suitable habitat distributions of *Hordeum spontaneum* under future climate scenarios. Each panel presents the spatial distribution of potential suitable habitats for *Hordeum spontaneum* under four shared socioeconomic pathway (SSP) climate scenarios (SSP1-2.6, SSP2-4.5, SSP3-7.0, SSP5-8.5) across three future time periods (2041–2060, 2061–2080, 2081–2100). Habitat suitability is categorized into five levels: unsuitable, low suitability, medium suitability, high suitability, and very high suitability, with a scale bar indicating an actual distance of 800 km.

**Supplementary Figure 17.** Predicted potential suitable habitat distributions of *Hordeum stenostachys* under future climate scenarios. Each panel presents the spatial distribution of potential suitable habitats for *Hordeum stenostachys* under four shared socioeconomic pathway (SSP) climate scenarios (SSP1-2.6, SSP2-4.5, SSP3-7.0, SSP5-8.5) across three future time periods (2041–2060, 2061–2080, 2081–2100). Habitat suitability is categorized into five levels: unsuitable, low suitability, medium suitability, high suitability, and very high suitability, with a scale bar indicating an actual distance of 800 km.

**Supplementary Figure 18.** Geographic distribution centroid migration pathways of *Hordeum agriocrithon* under future climate scenarios. This Figure illustrates the projected migration trajectories of the geographic distribution centroids for *Hordeum agriocrithon* under four shared socioeconomic pathway (SSP) climate scenarios (SSP1-2.6, SSP2-4.5, SSP3-7.0, SSP5-8.5) across three future time periods (2041–2060, 2061–2080, 2081–2100). Colored lines represent centroid movement under different climate scenarios, with endpoint symbols distinguishing the time periods, to visualize the direction and magnitude of range shifts driven by climate change.

**Supplementary Figure 19.** Geographic distribution centroid migration pathways under future climate scenarios: (A) *Hordeum bogdanii*; (B) *Hordeum brevisubulatum*.This Figure illustrates the projected migration trajectories of the geographic distribution centroids for *Hordeum bogdanii* (A) and *Hordeum brevisubulatum* (B) under four shared socioeconomic pathway (SSP) climate scenarios (SSP1-2.6, SSP2-4.5, SSP3-7.0, SSP5-8.5) across three future time periods (2041–2060, 2061–2080, 2081–2100). Distinct colors and line types represent centroid movements of the two species under different climate scenarios, with endpoint symbols distinguishing the time periods, to visualize and compare the direction, magnitude, and interspecific differences in range shifts driven by climate change.

**Supplementary Figure 20.** Geographic distribution centroid migration pathways of *Hordeum bulbosum* under future climate scenarios. This Figure illustrates the projected migration trajectories of the geographic distribution centroids for *Hordeum bulbosum* under four shared socioeconomic pathway (SSP) climate scenarios (SSP1-2.6, SSP2-4.5, SSP3-7.0, SSP5-8.5) across three future time periods (2041–2060, 2061–2080, 2081–2100). Colored lines represent centroid movement under different climate scenarios, with endpoint symbols distinguishing the time periods, to visualize the direction and magnitude of range shifts driven by climate change.

**Supplementary Figure 21.** Geographic distribution centroid migration pathways of *Hordeum californicum* under future climate scenarios. This Figure illustrates the projected migration trajectories of the geographic distribution centroids for *Hordeum californicum* under four shared socioeconomic pathway (SSP) climate scenarios (SSP1-2.6, SSP2-4.5, SSP3-7.0, SSP5-8.5) across three future time periods (2041–2060, 2061–2080, 2081–2100). Colored lines represent centroid movement under different climate scenarios, with endpoint symbols distinguishing the time periods, to visualize the direction and magnitude of range shifts driven by climate change.

**Supplementary Figure 22.** Geographic distribution centroid migration pathways of *Hordeum chilense* under future climate scenarios. This Figure illustrates the projected migration trajectories of the geographic distribution centroids for *Hordeum chilense* under four shared socioeconomic pathway (SSP) climate scenarios (SSP1-2.6, SSP2-4.5, SSP3-7.0, SSP5-8.5) across three future time periods (2041–2060, 2061–2080, 2081–2100). Colored lines represent centroid movement under different climate scenarios, with endpoint symbols distinguishing the time periods, to visualize the direction and magnitude of range shifts driven by climate change.

**Supplementary Figure 23.** Geographic distribution centroid migration pathways of *Hordeum marinum* under future climate scenarios. This Figure illustrates the projected migration trajectories of the geographic distribution centroids for *Hordeum marinum* under four shared socioeconomic pathway (SSP) climate scenarios (SSP1-2.6, SSP2-4.5, SSP3-7.0, SSP5-8.5) across three future time periods (2041–2060, 2061–2080, 2081–2100). Colored lines represent centroid movement under different climate scenarios, with endpoint symbols distinguishing the time periods, to visualize the direction and magnitude of range shifts driven by climate change.

**Supplementary Figure 24.** Geographic distribution centroid migration pathways of *Hordeum murinum* under future climate scenarios. This Figure illustrates the projected migration trajectories of the geographic distribution centroids for *Hordeum murinum* under four shared socioeconomic pathway (SSP) climate scenarios (SSP1-2.6, SSP2-4.5, SSP3-7.0, SSP5-8.5) across three future time periods (2041–2060, 2061–2080, 2081–2100). Colored lines represent centroid movement under different climate scenarios, with endpoint symbols distinguishing the time periods, to visualize the direction and magnitude of range shifts driven by climate change.

**Supplementary Figure 25.** Geographic distribution centroid migration pathways under future climate scenarios: (A) *Hordeum muticum*; (B) *Hordeum pusillum*.This Figure illustrates the projected migration trajectories of the geographic distribution centroids for *Hordeum muticum* (A) and *Hordeum pusillum* (B) under four shared socioeconomic pathway (SSP) climate scenarios (SSP1-2.6, SSP2-4.5, SSP3-7.0, SSP5-8.5) across three future time periods (2041–2060, 2061–2080, 2081–2100). Distinct colors and line types represent centroid movements of the two species under different climate scenarios, with endpoint symbols distinguishing the time periods, to visualize and compare the direction, magnitude, and interspecific differences in range shifts driven by climate change.

**Supplementary Figure 26.** Geographic distribution centroid migration pathways under future climate scenarios: (A) *Hordeum roshevitzii*; (B) *Hordeum spontaneum*.This Figure illustrates the projected migration trajectories of the geographic distribution centroids for *Hordeum roshevitzii* (a) and *Hordeum spontaneum* (b) under four shared socioeconomic pathway (SSP) climate scenarios (SSP1-2.6, SSP2-4.5, SSP3-7.0, SSP5-8.5) across three future time periods (2041–2060, 2061–2080, 2081–2100). Distinct colors and line types represent centroid movements of the two species under different climate scenarios, with endpoint symbols distinguishing the time periods, to visualize and compare the direction, magnitude, and interspecific differences in range shifts driven by climate change.

**Supplementary Figure 27.** Geographic distribution centroid migration pathways of *Hordeum stenostachys* under future climate scenarios. This Figure illustrates the projected migration trajectories of the geographic distribution centroids for *Hordeum stenostachys* under four shared socioeconomic pathway (SSP) climate scenarios (SSP1-2.6, SSP2-4.5, SSP3-7.0, SSP5-8.5) across three future time periods (2041–2060, 2061–2080, 2081–2100). Colored lines represent centroid movement under different climate scenarios, with endpoint symbols distinguishing the time periods, to visualize the direction and magnitude of range shifts driven by climate change.

## 1.2 Supplementary Table

**Supplementary Table 1.** Geographic distribution point information of 13 *Hordeum* species.

| Specise | Longitude | Latitude |
| --- | --- | --- |
| *Hordeum agriocrithon* A. E. Åberg | 102 | 30 |
| *Hordeum agriocrithon* A. E. Åberg | 97.85 | 29.67 |
| *Hordeum agriocrithon* A. E. Åberg | 101.12 | 30.98 |
| *Hordeum agriocrithon* A. E. Åberg | 86.4 | 42.32 |
| *Hordeum agriocrithon* A. E. Åberg | 101.43 | 36.05 |
| *Hordeum agriocrithon* A. E. Åberg | 116.21 | 40.02 |
| *Hordeum agriocrithon* A. E. Åberg | 94.36667 | 29.41667 |
| *Hordeum agriocrithon* A. E. Åberg | 94.367 | 29.417 |
| *Hordeum agriocrithon* A. E. Åberg | 88.892 | 29.264 |
| *Hordeum agriocrithon* A. E. Åberg | 101.263 | 36.728 |
| *Hordeum agriocrithon* A. E. Åberg | 101.363425 | 30.569713 |
| *Hordeum agriocrithon* A. E. Åberg | 91.107 | 29.658 |
| *Hordeum agriocrithon* A. E. Åberg | 96.978 | 33.032 |
| *Hordeum agriocrithon* A. E. Åberg | 100.182 | 31.647 |
| *Hordeum agriocrithon* A. E. Åberg | 98.675 | 29.698 |
| *Hordeum agriocrithon* A. E. Åberg | 101.95 | 30.05 |
| *Hordeum agriocrithon* A. E. Åberg | 91.13 | 29.65 |
| *Hordeum agriocrithon* A. E. Åberg | 96.78 | 33.05 |
| *Hordeum agriocrithon* A. E. Åberg | 88.87 | 29.25 |
| *Hordeum agriocrithon* A. E. Åberg | 102.45 | 32.05 |
| *Hordeum agriocrithon* A. E. Åberg | 101.18 | 30.92 |
| *Hordeum agriocrithon* A. E. Åberg | 94.25 | 29.15 |
| *Hordeum agriocrithon* A. E. Åberg | 96.45 | 32.28 |
| *Hordeum agriocrithon* A. E. Åberg | 89.63 | 28.95 |
| *Hordeum agriocrithon* A. E. Åberg | 102.25 | 31.95 |
| *Hordeum agriocrithon* A. E. Åberg | 95.68 | 31.45 |
| *Hordeum agriocrithon* A. E. Åberg | 100.28 | 34.42 |
| *Hordeum agriocrithon* A. E. Åberg | 101.23 | 27.98 |
| *Hordeum agriocrithon* A. E. Åberg | 91.22 | 29.68 |
| *Hordeum agriocrithon* A. E. Åberg | 101.48 | 36.05 |
| *Hordeum agriocrithon* A. E. Åberg | 100.68 | 31.35 |
| *Hordeum agriocrithon* A. E. Åberg | 79.85 | 31.42 |
| *Hordeum agriocrithon* A. E. Åberg | 100.28 | 30.05 |
| *Hordeum agriocrithon* A. E. Åberg | 102.08 | 35.52 |
| *Hordeum agriocrithon* A. E. Åberg | 98.15 | 33.05 |
| *Hordeum agriocrithon* A. E. Åberg | 92.48 | 28.45 |
| *Hordeum agriocrithon* A. E. Åberg | 101.98 | 36.85 |
| *Hordeum bogdanii* Wilensky | -1.54006 | 53.721559 |
| *Hordeum bogdanii* Wilensky | 101.52 | 34.57 |
| *Hordeum bogdanii* Wilensky | 100.62 | 36.28 |
| *Hordeum bogdanii* Wilensky | 103.68333 | 36.05 |
| *Hordeum bogdanii* Wilensky | 103.08 | 38.62 |
| *Hordeum bogdanii* Wilensky | 105 | 35 |
| *Hordeum bogdanii* Wilensky | 105.67 | 38.83 |
| *Hordeum bogdanii* Wilensky | 108.02 | 35.82 |
| *Hordeum bogdanii* Wilensky | 108.666667 | 48.2 |
| *Hordeum bogdanii* Wilensky | 113.833333 | 24.466667 |
| *Hordeum bogdanii* Wilensky | 114.566667 | 29.033333 |
| *Hordeum bogdanii* Wilensky | 115.67 | 37.07 |
| *Hordeum bogdanii* Wilensky | 116.45 | 38.383333 |
| *Hordeum bogdanii* Wilensky | 116.03333 | 43.96667 |
| *Hordeum bogdanii* Wilensky | 119.166667 | 47.5 |
| *Hordeum bogdanii* Wilensky | 120.08 | 30.21 |
| *Hordeum bogdanii* Wilensky | 122.06 | 46.06 |
| *Hordeum bogdanii* Wilensky | 35.921096 | 56.848837 |
| *Hordeum bogdanii* Wilensky | 44.54171 | 48.494067 |
| *Hordeum bogdanii* Wilensky | 46.666246 | 50.681241 |
| *Hordeum bogdanii* Wilensky | 47.630817 | 50.484967 |
| *Hordeum bogdanii* Wilensky | 47.858611 | 53.141667 |
| *Hordeum bogdanii* Wilensky | 49.1223 | 55.7969 |
| *Hordeum bogdanii* Wilensky | 52.215567 | 51.288745 |
| *Hordeum bogdanii* Wilensky | 52.708333 | 35.808333 |
| *Hordeum bogdanii* Wilensky | 56.6966 | 51.2266 |
| *Hordeum bogdanii* Wilensky | 55.9643 | 53.6528 |
| *Hordeum bogdanii* Wilensky | 59.64028 | 47.81778 |
| *Hordeum bogdanii* Wilensky | 57.6929 | 51.0751 |
| *Hordeum bogdanii* Wilensky | 61.525497 | 46.110016 |
| *Hordeum bogdanii* Wilensky | 61.2323 | 53.9348 |
| *Hordeum bogdanii* Wilensky | 62.056458 | 55.230179 |
| *Hordeum bogdanii* Wilensky | 64.13528 | 52.36381 |
| *Hordeum bogdanii* Wilensky | 63.63207 | 53.21448 |
| *Hordeum bogdanii* Wilensky | 67.27 | 34.87 |
| *Hordeum bogdanii* Wilensky | 65 | 36 |
| *Hordeum bogdanii* Wilensky | 67.023296 | 48.601644 |
| *Hordeum bogdanii* Wilensky | 68.4 | 33.7 |
| *Hordeum bogdanii* Wilensky | 69.02 | 35.4 |
| *Hordeum bogdanii* Wilensky | 72.35 | 36.35 |
| *Hordeum bogdanii* Wilensky | 70.807 | 38.643 |
| *Hordeum bogdanii* Wilensky | 71.78333 | 43.01667 |
| *Hordeum bogdanii* Wilensky | 74.65 | 36.87 |
| *Hordeum bogdanii* Wilensky | 74.335727 | 39.821029 |
| *Hordeum bogdanii* Wilensky | 74.655556 | 41.296389 |
| *Hordeum bogdanii* Wilensky | 73.174429 | 49.852268 |
| *Hordeum bogdanii* Wilensky | 75.73 | 35.43 |
| *Hordeum bogdanii* Wilensky | 75.42 | 38.97 |
| *Hordeum bogdanii* Wilensky | 75.48251 | 49.37582 |
| *Hordeum bogdanii* Wilensky | 77.55 | 34.75 |
| *Hordeum bogdanii* Wilensky | 79.86667 | 43.01667 |
| *Hordeum bogdanii* Wilensky | 79.6813 | 51.8338 |
| *Hordeum bogdanii* Wilensky | 80.38 | 40.63 |
| *Hordeum bogdanii* Wilensky | 81.83 | 43.22 |
| *Hordeum bogdanii* Wilensky | 81.0918 | 51.4434 |
| *Hordeum bogdanii* Wilensky | 84.975171 | 41.282539 |
| *Hordeum bogdanii* Wilensky | 82.88 | 44.6 |
| *Hordeum bogdanii* Wilensky | 82.98 | 46.2 |
| *Hordeum bogdanii* Wilensky | 82.625 | 52.487 |
| *Hordeum bogdanii* Wilensky | 86 | 42 |
| *Hordeum bogdanii* Wilensky | 87.26667 | 43.46667 |
| *Hordeum bogdanii* Wilensky | 85.72 | 46.8 |
| *Hordeum bogdanii* Wilensky | 86.42 | 48.07 |
| *Hordeum bogdanii* Wilensky | 88.118532 | 31.712092 |
| *Hordeum bogdanii* Wilensky | 87.91667 | 44.13333 |
| *Hordeum bogdanii* Wilensky | 89.843333 | 46.979167 |
| *Hordeum bogdanii* Wilensky | 88.135278 | 47.842778 |
| *Hordeum bogdanii* Wilensky | 90.28 | 43.83 |
| *Hordeum bogdanii* Wilensky | 90.38 | 46.67 |
| *Hordeum bogdanii* Wilensky | 92.25 | 49.917 |
| *Hordeum bogdanii* Wilensky | 94.9 | 36.42 |
| *Hordeum bogdanii* Wilensky | 94.88 | 39.52 |
| *Hordeum bogdanii* Wilensky | 94.67 | 40.13 |
| *Hordeum bogdanii* Wilensky | 94.7 | 43.25 |
| *Hordeum bogdanii* Wilensky | 92.836833 | 47.017017 |
| *Hordeum bogdanii* Wilensky | 93.171 | 50.6136 |
| *Hordeum bogdanii* Wilensky | 97.37 | 37.37 |
| *Hordeum bogdanii* Wilensky | 97.05 | 40.28 |
| *Hordeum bogdanii* Wilensky | 99.408333 | 27.608333 |
| *Hordeum bogdanii* Wilensky | 99.62 | 38.83 |
| *Hordeum brevisubulatum* (Trin.) Link | -173.5755 | 64.4446 |
| *Hordeum brevisubulatum* (Trin.) Link | -1.2916 | 50.700802 |
| *Hordeum brevisubulatum* (Trin.) Link | -1.718167 | 53.657667 |
| *Hordeum brevisubulatum* (Trin.) Link | -2.8385 | 55.550701 |
| *Hordeum brevisubulatum* (Trin.) Link | 100.62 | 36.28 |
| *Hordeum brevisubulatum* (Trin.) Link | 101.97 | 38.25 |
| *Hordeum brevisubulatum* (Trin.) Link | 101.429663 | 47.47315 |
| *Hordeum brevisubulatum* (Trin.) Link | 100.483927 | 48.469376 |
| *Hordeum brevisubulatum* (Trin.) Link | 100.254681 | 50.654686 |
| *Hordeum brevisubulatum* (Trin.) Link | 102.497 | 53.5534 |
| *Hordeum brevisubulatum* (Trin.) Link | 101.73 | 55.582 |
| *Hordeum brevisubulatum* (Trin.) Link | 103.891767 | 36.519977 |
| *Hordeum brevisubulatum* (Trin.) Link | 104.080077 | 43.491926 |
| *Hordeum brevisubulatum* (Trin.) Link | 103.312639 | 46.6618 |
| *Hordeum brevisubulatum* (Trin.) Link | 104.331231 | 47.847778 |
| *Hordeum brevisubulatum* (Trin.) Link | 103.714583 | 51.659363 |
| *Hordeum brevisubulatum* (Trin.) Link | 103.545746 | 52.817011 |
| *Hordeum brevisubulatum* (Trin.) Link | 103.304 | 56.236 |
| *Hordeum brevisubulatum* (Trin.) Link | 106.57 | 29.6 |
| *Hordeum brevisubulatum* (Trin.) Link | 105.72 | 34.56 |
| *Hordeum brevisubulatum* (Trin.) Link | 107.4 | 37.78 |
| *Hordeum brevisubulatum* (Trin.) Link | 107.137644 | 47.802542 |
| *Hordeum brevisubulatum* (Trin.) Link | 105.529516 | 51.644942 |
| *Hordeum brevisubulatum* (Trin.) Link | 106.427169 | 52.645797 |
| *Hordeum brevisubulatum* (Trin.) Link | 109.32 | 36.87 |
| *Hordeum brevisubulatum* (Trin.) Link | 107.98 | 39.1 |
| *Hordeum brevisubulatum* (Trin.) Link | 108.392296 | 47.594515 |
| *Hordeum brevisubulatum* (Trin.) Link | 107.701095 | 51.825583 |
| *Hordeum brevisubulatum* (Trin.) Link | 109.983178 | 53.90382 |
| *Hordeum brevisubulatum* (Trin.) Link | 109.7311 | 55.7576 |
| *Hordeum brevisubulatum* (Trin.) Link | 108.2052 | 59.2613 |
| *Hordeum brevisubulatum* (Trin.) Link | 108.0065 | 61.2749 |
| *Hordeum brevisubulatum* (Trin.) Link | 108.397 | 62.9524 |
| *Hordeum brevisubulatum* (Trin.) Link | 11.28333 | 51.81667 |
| *Hordeum brevisubulatum* (Trin.) Link | 110.25 | 37.5 |
| *Hordeum brevisubulatum* (Trin.) Link | 111.65444 | 40.815 |
| *Hordeum brevisubulatum* (Trin.) Link | 112 | 44 |
| *Hordeum brevisubulatum* (Trin.) Link | 110.62786 | 47.270493 |
| *Hordeum brevisubulatum* (Trin.) Link | 110.371147 | 48.219208 |
| *Hordeum brevisubulatum* (Trin.) Link | 110.783203 | 52.409195 |
| *Hordeum brevisubulatum* (Trin.) Link | 110.295 | 53.7147 |
| *Hordeum brevisubulatum* (Trin.) Link | 111.4096 | 55.1546 |
| *Hordeum brevisubulatum* (Trin.) Link | 111.0527 | 59.4711 |
| *Hordeum brevisubulatum* (Trin.) Link | 113.833333 | 24.466667 |
| *Hordeum brevisubulatum* (Trin.) Link | 114.566667 | 29.033333 |
| *Hordeum brevisubulatum* (Trin.) Link | 113.8 | 35.2 |
| *Hordeum brevisubulatum* (Trin.) Link | 114 | 41.9 |
| *Hordeum brevisubulatum* (Trin.) Link | 112.65 | 42.75 |
| *Hordeum brevisubulatum* (Trin.) Link | 114.083333 | 45.033333 |
| *Hordeum brevisubulatum* (Trin.) Link | 114.995597 | 47.918808 |
| *Hordeum brevisubulatum* (Trin.) Link | 113.236825 | 52.050561 |
| *Hordeum brevisubulatum* (Trin.) Link | 114.46 | 54.919 |
| *Hordeum brevisubulatum* (Trin.) Link | 114.4765 | 55.5817 |
| *Hordeum brevisubulatum* (Trin.) Link | 112.7134 | 59.1675 |
| *Hordeum brevisubulatum* (Trin.) Link | 113.5025 | 60.2272 |
| *Hordeum brevisubulatum* (Trin.) Link | 116.666668 | 28.616665 |
| *Hordeum brevisubulatum* (Trin.) Link | 116.37 | 39.92 |
| *Hordeum brevisubulatum* (Trin.) Link | 116 | 42.25 |
| *Hordeum brevisubulatum* (Trin.) Link | 116.03333 | 43.96667 |
| *Hordeum brevisubulatum* (Trin.) Link | 116.97 | 45.52 |
| *Hordeum brevisubulatum* (Trin.) Link | 116.82 | 48.67 |
| *Hordeum brevisubulatum* (Trin.) Link | 116.384042 | 50.519611 |
| *Hordeum brevisubulatum* (Trin.) Link | 116.489 | 52.978 |
| *Hordeum brevisubulatum* (Trin.) Link | 115.713 | 56.696 |
| *Hordeum brevisubulatum* (Trin.) Link | 115.269708 | 58.447762 |
| *Hordeum brevisubulatum* (Trin.) Link | 115.1059 | 60.9839 |
| *Hordeum brevisubulatum* (Trin.) Link | 117.1947 | 62.5986 |
| *Hordeum brevisubulatum* (Trin.) Link | 116.71922 | 65.04247 |
| *Hordeum brevisubulatum* (Trin.) Link | 119.92 | 42.29 |
| *Hordeum brevisubulatum* (Trin.) Link | 119.02 | 42.93 |
| *Hordeum brevisubulatum* (Trin.) Link | 119.93 | 47.18 |
| *Hordeum brevisubulatum* (Trin.) Link | 119.77 | 49.22 |
| *Hordeum brevisubulatum* (Trin.) Link | 117.943147 | 50.936723 |
| *Hordeum brevisubulatum* (Trin.) Link | 118.2595 | 56.8144 |
| *Hordeum brevisubulatum* (Trin.) Link | 117.688 | 57.592 |
| *Hordeum brevisubulatum* (Trin.) Link | 118.6511 | 62.0289 |
| *Hordeum brevisubulatum* (Trin.) Link | 120.051667 | 27.664722 |
| *Hordeum brevisubulatum* (Trin.) Link | 121.8 | 41.6 |
| *Hordeum brevisubulatum* (Trin.) Link | 122.35 | 42.95 |
| *Hordeum brevisubulatum* (Trin.) Link | 122.05 | 46.08 |
| *Hordeum brevisubulatum* (Trin.) Link | 120.73 | 49.28 |
| *Hordeum brevisubulatum* (Trin.) Link | 122.0156 | 54.2988 |
| *Hordeum brevisubulatum* (Trin.) Link | 122.2274 | 58.7062 |
| *Hordeum brevisubulatum* (Trin.) Link | 120.063611 | 60.658611 |
| *Hordeum brevisubulatum* (Trin.) Link | 121.598208 | 63.7375 |
| *Hordeum brevisubulatum* (Trin.) Link | 123.842343 | 42.286255 |
| *Hordeum brevisubulatum* (Trin.) Link | 123.5 | 43.52 |
| *Hordeum brevisubulatum* (Trin.) Link | 124.852127 | 46.638625 |
| *Hordeum brevisubulatum* (Trin.) Link | 122.75 | 47.98 |
| *Hordeum brevisubulatum* (Trin.) Link | 123.896165 | 53.475606 |
| *Hordeum brevisubulatum* (Trin.) Link | 124.5661 | 55.245 |
| *Hordeum brevisubulatum* (Trin.) Link | 122.5177 | 64.4657 |
| *Hordeum brevisubulatum* (Trin.) Link | 122.676 | 67.252 |
| *Hordeum brevisubulatum* (Trin.) Link | 125.9 | 47.18 |
| *Hordeum brevisubulatum* (Trin.) Link | 125.87 | 48.03 |
| *Hordeum brevisubulatum* (Trin.) Link | 127.1653 | 50.9813 |
| *Hordeum brevisubulatum* (Trin.) Link | 127.234887 | 53.71472 |
| *Hordeum brevisubulatum* (Trin.) Link | 125.472 | 57.945 |
| *Hordeum brevisubulatum* (Trin.) Link | 127.015 | 60.8157 |
| *Hordeum brevisubulatum* (Trin.) Link | 127.46642 | 63.923462 |
| *Hordeum brevisubulatum* (Trin.) Link | 127.4015 | 70.798 |
| *Hordeum brevisubulatum* (Trin.) Link | 128.2 | 41.42 |
| *Hordeum brevisubulatum* (Trin.) Link | 127.989653 | 49.614986 |
| *Hordeum brevisubulatum* (Trin.) Link | 127.5312 | 50.402 |
| *Hordeum brevisubulatum* (Trin.) Link | 127.8348 | 52.9712 |
| *Hordeum brevisubulatum* (Trin.) Link | 127.741 | 59.722 |
| *Hordeum brevisubulatum* (Trin.) Link | 128.057652 | 62.14497 |
| *Hordeum brevisubulatum* (Trin.) Link | 129.8391 | 62.5606 |
| *Hordeum brevisubulatum* (Trin.) Link | 128.81 | 71.6477 |
| *Hordeum brevisubulatum* (Trin.) Link | 131.9149 | 43.6542 |
| *Hordeum brevisubulatum* (Trin.) Link | 130.9504 | 48.4818 |
| *Hordeum brevisubulatum* (Trin.) Link | 130.0904 | 62.326266 |
| *Hordeum brevisubulatum* (Trin.) Link | 131.0848 | 62.8053 |
| *Hordeum brevisubulatum* (Trin.) Link | 131.7176 | 65.8257 |
| *Hordeum brevisubulatum* (Trin.) Link | 131.633 | 67.513 |
| *Hordeum brevisubulatum* (Trin.) Link | 132.5043 | 44.1154 |
| *Hordeum brevisubulatum* (Trin.) Link | 134.0705 | 45.9457 |
| *Hordeum brevisubulatum* (Trin.) Link | 134.2467 | 48.3923 |
| *Hordeum brevisubulatum* (Trin.) Link | 134.1447 | 58.7918 |
| *Hordeum brevisubulatum* (Trin.) Link | 134.2514 | 62.1961 |
| *Hordeum brevisubulatum* (Trin.) Link | 133.5926 | 62.6328 |
| *Hordeum brevisubulatum* (Trin.) Link | 132.7613 | 65.4125 |
| *Hordeum brevisubulatum* (Trin.) Link | 132.62 | 67.853 |
| *Hordeum brevisubulatum* (Trin.) Link | 135.4149 | 48.5143 |
| *Hordeum brevisubulatum* (Trin.) Link | 136.8314 | 50.5603 |
| *Hordeum brevisubulatum* (Trin.) Link | 136.368 | 58.2854 |
| *Hordeum brevisubulatum* (Trin.) Link | 135.404 | 62.523 |
| *Hordeum brevisubulatum* (Trin.) Link | 137.5322 | 62.926 |
| *Hordeum brevisubulatum* (Trin.) Link | 143.272923 | 63.402283 |
| *Hordeum brevisubulatum* (Trin.) Link | 146.8267 | 64.7436 |
| *Hordeum brevisubulatum* (Trin.) Link | 149.2131 | 59.8162 |
| *Hordeum brevisubulatum* (Trin.) Link | 147.8833 | 70.6333 |
| *Hordeum brevisubulatum* (Trin.) Link | 150.8124 | 59.6236 |
| *Hordeum brevisubulatum* (Trin.) Link | 15.33722 | 49.56222 |
| *Hordeum brevisubulatum* (Trin.) Link | 4.90993 | 52.3744 |
| *Hordeum brevisubulatum* (Trin.) Link | 22.977033 | 59.774082 |
| *Hordeum brevisubulatum* (Trin.) Link | 32.1481 | 40.7942 |
| *Hordeum brevisubulatum* (Trin.) Link | 30.305286 | 59.942155 |
| *Hordeum brevisubulatum* (Trin.) Link | 35.179672 | 39.060596 |
| *Hordeum brevisubulatum* (Trin.) Link | 35.9119 | 56.8596 |
| *Hordeum brevisubulatum* (Trin.) Link | 39.46667 | 40.45 |
| *Hordeum brevisubulatum* (Trin.) Link | 41.3 | 39.98333 |
| *Hordeum brevisubulatum* (Trin.) Link | 42.152489 | 43.775028 |
| *Hordeum brevisubulatum* (Trin.) Link | 41.96667 | 45.03333 |
| *Hordeum brevisubulatum* (Trin.) Link | 41.0102 | 57.061 |
| *Hordeum brevisubulatum* (Trin.) Link | 43.25 | 38.28333 |
| *Hordeum brevisubulatum* (Trin.) Link | 44.950001 | 40.580002 |
| *Hordeum brevisubulatum* (Trin.) Link | 42.603467 | 43.717098 |
| *Hordeum brevisubulatum* (Trin.) Link | 47.016667 | 35.95 |
| *Hordeum brevisubulatum* (Trin.) Link | 46.49944 | 37.73556 |
| *Hordeum brevisubulatum* (Trin.) Link | 45.23 | 40.029999 |
| *Hordeum brevisubulatum* (Trin.) Link | 46.128141 | 42.764239 |
| *Hordeum brevisubulatum* (Trin.) Link | 47.057687 | 50.916846 |
| *Hordeum brevisubulatum* (Trin.) Link | 49.93052 | 34.57073 |
| *Hordeum brevisubulatum* (Trin.) Link | 48.83333 | 36.48333 |
| *Hordeum brevisubulatum* (Trin.) Link | 47.91667 | 38.51667 |
| *Hordeum brevisubulatum* (Trin.) Link | 48.00861 | 41.20639 |
| *Hordeum brevisubulatum* (Trin.) Link | 49.4668 | 53.8022 |
| *Hordeum brevisubulatum* (Trin.) Link | 50.76667 | 32.05 |
| *Hordeum brevisubulatum* (Trin.) Link | 51.383333 | 36.216667 |
| *Hordeum brevisubulatum* (Trin.) Link | 51.047003 | 53.930933 |
| *Hordeum brevisubulatum* (Trin.) Link | 53 | 32 |
| *Hordeum brevisubulatum* (Trin.) Link | 53.2 | 35.95 |
| *Hordeum brevisubulatum* (Trin.) Link | 54.5181 | 53.7958 |
| *Hordeum brevisubulatum* (Trin.) Link | 56.4569 | 52.3427 |
| *Hordeum brevisubulatum* (Trin.) Link | 55.1086 | 53.6368 |
| *Hordeum brevisubulatum* (Trin.) Link | 58 | 37.83333 |
| *Hordeum brevisubulatum* (Trin.) Link | 59.166667 | 47.366667 |
| *Hordeum brevisubulatum* (Trin.) Link | 59.64028 | 47.81778 |
| *Hordeum brevisubulatum* (Trin.) Link | 58.3159 | 52.0519 |
| *Hordeum brevisubulatum* (Trin.) Link | 59.1478 | 53.1564 |
| *Hordeum brevisubulatum* (Trin.) Link | 58.9116 | 55.0174 |
| *Hordeum brevisubulatum* (Trin.) Link | 5.07711 | 51.56602 |
| *Hordeum brevisubulatum* (Trin.) Link | 60.807266 | 47.435751 |
| *Hordeum brevisubulatum* (Trin.) Link | 61.4406 | 53.1779 |
| *Hordeum brevisubulatum* (Trin.) Link | 61.71962 | 56.378072 |
| *Hordeum brevisubulatum* (Trin.) Link | 63.16944 | 41.75056 |
| *Hordeum brevisubulatum* (Trin.) Link | 64.795575 | 51.50577 |
| *Hordeum brevisubulatum* (Trin.) Link | 63.905 | 54.2681 |
| *Hordeum brevisubulatum* (Trin.) Link | 64.508077 | 55.17025 |
| *Hordeum brevisubulatum* (Trin.) Link | 67.18 | 34.82 |
| *Hordeum brevisubulatum* (Trin.) Link | 66.2143 | 54.7609 |
| *Hordeum brevisubulatum* (Trin.) Link | 66.017744 | 56.257601 |
| *Hordeum brevisubulatum* (Trin.) Link | 68.32 | 34.47 |
| *Hordeum brevisubulatum* (Trin.) Link | 69 | 35.42 |
| *Hordeum brevisubulatum* (Trin.) Link | 68.30153 | 39.05748 |
| *Hordeum brevisubulatum* (Trin.) Link | 69.3 | 41.33333 |
| *Hordeum brevisubulatum* (Trin.) Link | 69.300338 | 55.954647 |
| *Hordeum brevisubulatum* (Trin.) Link | 68.4514 | 58.2986 |
| *Hordeum brevisubulatum* (Trin.) Link | 72.42 | 36.1 |
| *Hordeum brevisubulatum* (Trin.) Link | 72 | 37.67 |
| *Hordeum brevisubulatum* (Trin.) Link | 70.05778 | 41.49444 |
| *Hordeum brevisubulatum* (Trin.) Link | 72.316474 | 42.6068 |
| *Hordeum brevisubulatum* (Trin.) Link | 71.35 | 51.33333 |
| *Hordeum brevisubulatum* (Trin.) Link | 70.177 | 56.509 |
| *Hordeum brevisubulatum* (Trin.) Link | 74.38 | 36.82 |
| *Hordeum brevisubulatum* (Trin.) Link | 72.88 | 38.63 |
| *Hordeum brevisubulatum* (Trin.) Link | 73.34028 | 40.24556 |
| *Hordeum brevisubulatum* (Trin.) Link | 74.66303 | 42.59742 |
| *Hordeum brevisubulatum* (Trin.) Link | 72.85 | 49.83 |
| *Hordeum brevisubulatum* (Trin.) Link | 73.13222 | 51.57333 |
| *Hordeum brevisubulatum* (Trin.) Link | 73.3682 | 54.9893 |
| *Hordeum brevisubulatum* (Trin.) Link | 72.760826 | 56.521738 |
| *Hordeum brevisubulatum* (Trin.) Link | 77.48 | 33.65 |
| *Hordeum brevisubulatum* (Trin.) Link | 76.95 | 36.45 |
| *Hordeum brevisubulatum* (Trin.) Link | 75.42 | 38.97 |
| *Hordeum brevisubulatum* (Trin.) Link | 75.86667 | 41.21667 |
| *Hordeum brevisubulatum* (Trin.) Link | 76.9514 | 43.1115 |
| *Hordeum brevisubulatum* (Trin.) Link | 76.016 | 54.578 |
| *Hordeum brevisubulatum* (Trin.) Link | 77.01 | 55.224 |
| *Hordeum brevisubulatum* (Trin.) Link | 79.616202 | 22.883478 |
| *Hordeum brevisubulatum* (Trin.) Link | 79.8 | 31.48 |
| *Hordeum brevisubulatum* (Trin.) Link | 78.03 | 32.95 |
| *Hordeum brevisubulatum* (Trin.) Link | 77.75 | 36.42 |
| *Hordeum brevisubulatum* (Trin.) Link | 77.881667 | 41.604444 |
| *Hordeum brevisubulatum* (Trin.) Link | 79.4325 | 44.27694 |
| *Hordeum brevisubulatum* (Trin.) Link | 78.324449 | 45.244722 |
| *Hordeum brevisubulatum* (Trin.) Link | 77.710612 | 52.298255 |
| *Hordeum brevisubulatum* (Trin.) Link | 78.393944 | 53.42189 |
| *Hordeum brevisubulatum* (Trin.) Link | 79.78875 | 55.007202 |
| *Hordeum brevisubulatum* (Trin.) Link | 80.1 | 32.02 |
| *Hordeum brevisubulatum* (Trin.) Link | 80.1 | 32.5 |
| *Hordeum brevisubulatum* (Trin.) Link | 80.8 | 37 |
| *Hordeum brevisubulatum* (Trin.) Link | 80.27 | 41.17 |
| *Hordeum brevisubulatum* (Trin.) Link | 81.32 | 43.92 |
| *Hordeum brevisubulatum* (Trin.) Link | 82.047388 | 52.177718 |
| *Hordeum brevisubulatum* (Trin.) Link | 81.237624 | 54.00026 |
| *Hordeum brevisubulatum* (Trin.) Link | 81.046511 | 55.084608 |
| *Hordeum brevisubulatum* (Trin.) Link | 84.02 | 28.67 |
| *Hordeum brevisubulatum* (Trin.) Link | 82.68 | 37.07 |
| *Hordeum brevisubulatum* (Trin.) Link | 82.96 | 41.71 |
| *Hordeum brevisubulatum* (Trin.) Link | 84.68 | 44.43 |
| *Hordeum brevisubulatum* (Trin.) Link | 82.98 | 46.75 |
| *Hordeum brevisubulatum* (Trin.) Link | 82.768937 | 52.479633 |
| *Hordeum brevisubulatum* (Trin.) Link | 83.497527 | 52.898852 |
| *Hordeum brevisubulatum* (Trin.) Link | 86.4 | 42.32 |
| *Hordeum brevisubulatum* (Trin.) Link | 85.5 | 42.6 |
| *Hordeum brevisubulatum* (Trin.) Link | 85.72 | 46.8 |
| *Hordeum brevisubulatum* (Trin.) Link | 85.42833 | 49.18778 |
| *Hordeum brevisubulatum* (Trin.) Link | 85.58333 | 51.58333 |
| *Hordeum brevisubulatum* (Trin.) Link | 85.37346 | 54.57919 |
| *Hordeum brevisubulatum* (Trin.) Link | 86.019 | 55.189 |
| *Hordeum brevisubulatum* (Trin.) Link | 88.118532 | 31.712092 |
| *Hordeum brevisubulatum* (Trin.) Link | 87.6 | 43.8 |
| *Hordeum brevisubulatum* (Trin.) Link | 89.52 | 47 |
| *Hordeum brevisubulatum* (Trin.) Link | 88.194039 | 49.066822 |
| *Hordeum brevisubulatum* (Trin.) Link | 88.311174 | 50.1589 |
| *Hordeum brevisubulatum* (Trin.) Link | 89.786275 | 54.498745 |
| *Hordeum brevisubulatum* (Trin.) Link | 88.992 | 55.036 |
| *Hordeum brevisubulatum* (Trin.) Link | 92.333581 | 46.023506 |
| *Hordeum brevisubulatum* (Trin.) Link | 91.129447 | 50.20135 |
| *Hordeum brevisubulatum* (Trin.) Link | 92.1963 | 53.8517 |
| *Hordeum brevisubulatum* (Trin.) Link | 90.223 | 56.0256 |
| *Hordeum brevisubulatum* (Trin.) Link | 92.479 | 62.422 |
| *Hordeum brevisubulatum* (Trin.) Link | 94.9 | 36.42 |
| *Hordeum brevisubulatum* (Trin.) Link | 94.88 | 39.52 |
| *Hordeum brevisubulatum* (Trin.) Link | 92.801807 | 47.649303 |
| *Hordeum brevisubulatum* (Trin.) Link | 93.7365 | 52.2226 |
| *Hordeum brevisubulatum* (Trin.) Link | 93.3423 | 52.8619 |
| *Hordeum brevisubulatum* (Trin.) Link | 92.9231 | 55.9914 |
| *Hordeum brevisubulatum* (Trin.) Link | 97.37 | 37.37 |
| *Hordeum brevisubulatum* (Trin.) Link | 97.463653 | 48.161408 |
| *Hordeum brevisubulatum* (Trin.) Link | 95.606 | 50.5454 |
| *Hordeum brevisubulatum* (Trin.) Link | 95.566 | 53.022 |
| *Hordeum brevisubulatum* (Trin.) Link | 95.9665 | 56.5661 |
| *Hordeum brevisubulatum* (Trin.) Link | 96.142 | 64.377 |
| *Hordeum brevisubulatum* (Trin.) Link | 99.98 | 35.58 |
| *Hordeum brevisubulatum* (Trin.) Link | 99.76327 | 48.148983 |
| *Hordeum brevisubulatum* (Trin.) Link | 98.993056 | 50.37 |
| *Hordeum brevisubulatum* (Trin.) Link | 99.822386 | 52.51427 |
| *Hordeum brevisubulatum* (Trin.) Link | 97.607 | 55.837 |
| *Hordeum brevisubulatum* (Trin.) Link | 97.829 | 60.888 |
| *Hordeum brevisubulatum* (Trin.) Link | 99.727 | 64.409 |
| *Hordeum bulbosum* L. | -106.778337 | 32.312316 |
| *Hordeum bulbosum* L. | -8.1 | 37.82 |
| *Hordeum bulbosum* L. | -107.851447 | 35.14726 |
| *Hordeum bulbosum* L. | -111.776067 | 42.986307 |
| *Hordeum bulbosum* L. | -110.969106 | 45.505902 |
| *Hordeum bulbosum* L. | -114.804929 | 44.029628 |
| *Hordeum bulbosum* L. | -113.45 | 45.61667 |
| *Hordeum bulbosum* L. | -116.76641 | 32.83505 |
| *Hordeum bulbosum* L. | -117.000214 | 43.273298 |
| *Hordeum bulbosum* L. | -115.541333 | 45.96764 |
| *Hordeum bulbosum* L. | -119.416629 | 37.079048 |
| *Hordeum bulbosum* L. | -119.664658 | 38.727199 |
| *Hordeum bulbosum* L. | -119.663269 | 40.73132 |
| *Hordeum bulbosum* L. | -118.192712 | 44.437384 |
| *Hordeum bulbosum* L. | -120.379925 | 39.323238 |
| *Hordeum bulbosum* L. | -122.177082 | 48.051764 |
| *Hordeum bulbosum* L. | -122.509445 | 37.521053 |
| *Hordeum bulbosum* L. | -123.035 | 44.9429 |
| *Hordeum bulbosum* L. | -1.31905 | 34.88921 |
| *Hordeum bulbosum* L. | -0.05454 | 45.63294 |
| *Hordeum bulbosum* L. | -0.16219 | 51.48455 |
| *Hordeum bulbosum* L. | -2 | 53 |
| *Hordeum bulbosum* L. | -25.7014967 | 37.7185213 |
| *Hordeum bulbosum* L. | -56 | -33 |
| *Hordeum bulbosum* L. | -5 | 32 |
| *Hordeum bulbosum* L. | -4.110271 | 34.029602 |
| *Hordeum bulbosum* L. | -4.72 | 36.93 |
| *Hordeum bulbosum* L. | -4.82 | 37.76 |
| *Hordeum bulbosum* L. | -3.66667 | 40.5 |
| *Hordeum bulbosum* L. | -5.66667 | 34.7667 |
| *Hordeum bulbosum* L. | -5.61 | 36.07 |
| *Hordeum bulbosum* L. | -5.38 | 39.38 |
| *Hordeum bulbosum* L. | -5.39 | 41.439999 |
| *Hordeum bulbosum* L. | -6.04 | 44.93 |
| *Hordeum bulbosum* L. | -72.25416 | 41.80916 |
| *Hordeum bulbosum* L. | -88.818387 | 33.4504 |
| *Hordeum bulbosum* L. | -89.01 | 41.34055 |
| *Hordeum bulbosum* L. | -90 | 45 |
| *Hordeum bulbosum* L. | -90.947086 | 40.931982 |
| *Hordeum bulbosum* L. | -97.147907 | 33.212181 |
| *Hordeum bulbosum* L. | -97.03833 | 37.06194 |
| *Hordeum bulbosum* L. | 2.2543 | 48.79645 |
| *Hordeum bulbosum* L. | 2.308541 | 51.265504 |
| *Hordeum bulbosum* L. | 12.468645 | 37.867149 |
| *Hordeum bulbosum* L. | 11.35 | 42.433333 |
| *Hordeum bulbosum* L. | 11.486805 | 43.825353 |
| *Hordeum bulbosum* L. | 11.216 | 46.05 |
| *Hordeum bulbosum* L. | 10.89333 | 49.9 |
| *Hordeum bulbosum* L. | 10.5 | 51.5 |
| *Hordeum bulbosum* L. | 12.3772 | 54.9768 |
| *Hordeum bulbosum* L. | 10.671 | 63.434 |
| *Hordeum bulbosum* L. | 113.8 | 35.2 |
| *Hordeum bulbosum* L. | 116.21 | 40.02 |
| *Hordeum bulbosum* L. | 118.79 | 32.05 |
| *Hordeum bulbosum* L. | 14.23067 | 37.08034 |
| *Hordeum bulbosum* L. | 14.9 | 37.87 |
| *Hordeum bulbosum* L. | 12.563307 | 41.933131 |
| *Hordeum bulbosum* L. | 13.80413 | 44.921325 |
| *Hordeum bulbosum* L. | 13.62167 | 45.11139 |
| *Hordeum bulbosum* L. | 14.98305 | 51.157217 |
| *Hordeum bulbosum* L. | 13.3151 | 53.21 |
| *Hordeum bulbosum* L. | 13.02351 | 55.59028 |
| *Hordeum bulbosum* L. | 127.9377 | 26.9344 |
| *Hordeum bulbosum* L. | 138.6 | -34.916667 |
| *Hordeum bulbosum* L. | 147.133333 | -41.466667 |
| *Hordeum bulbosum* L. | 149.13105 | -32.250463 |
| *Hordeum bulbosum* L. | 149.231632 | -34.022774 |
| *Hordeum bulbosum* L. | 149.15 | -35.25 |
| *Hordeum bulbosum* L. | 152.08442 | -28.248408 |
| *Hordeum bulbosum* L. | 17 | 25 |
| *Hordeum bulbosum* L. | 15.27306 | 37.08528 |
| *Hordeum bulbosum* L. | 15.47 | 38.18 |
| *Hordeum bulbosum* L. | 15.083333 | 41.883331 |
| *Hordeum bulbosum* L. | 16.537961 | 43.60078 |
| *Hordeum bulbosum* L. | 17.18924 | 49.77838 |
| *Hordeum bulbosum* L. | 19.9 | 39.616667 |
| *Hordeum bulbosum* L. | 17.908996 | 40.302596 |
| *Hordeum bulbosum* L. | 4.26667 | 36.7167 |
| *Hordeum bulbosum* L. | 3.89693 | 43.60708 |
| *Hordeum bulbosum* L. | 4.09473 | 45.81933 |
| *Hordeum bulbosum* L. | 4.8995 | 52.06525 |
| *Hordeum bulbosum* L. | 4.82795 | 53.04808 |
| *Hordeum bulbosum* L. | 21.91174 | 32 |
| *Hordeum bulbosum* L. | 20.9 | 32.5 |
| *Hordeum bulbosum* L. | 22.416667 | 36.583333 |
| *Hordeum bulbosum* L. | 21.47 | 39.74 |
| *Hordeum bulbosum* L. | 22.133333 | 40.816667 |
| *Hordeum bulbosum* L. | 22.31694 | 44.645 |
| *Hordeum bulbosum* L. | 20 | 47 |
| *Hordeum bulbosum* L. | 24.5 | 37.15 |
| *Hordeum bulbosum* L. | 24.816667 | 37.833333 |
| *Hordeum bulbosum* L. | 23.11111 | 40.59583 |
| *Hordeum bulbosum* L. | 23.74833 | 43.19556 |
| *Hordeum bulbosum* L. | 24.9688 | 45.8436 |
| *Hordeum bulbosum* L. | 26.033333 | 36.9 |
| *Hordeum bulbosum* L. | 26.066667 | 38.2 |
| *Hordeum bulbosum* L. | 26.05 | 40.933333 |
| *Hordeum bulbosum* L. | 25 | 43 |
| *Hordeum bulbosum* L. | 25.838432 | 52.518179 |
| *Hordeum bulbosum* L. | 27.583333 | 36.233333 |
| *Hordeum bulbosum* L. | 28.014997 | 39.542463 |
| *Hordeum bulbosum* L. | 27.79142 | 42.12011 |
| *Hordeum bulbosum* L. | 27.75889 | 42.71389 |
| *Hordeum bulbosum* L. | 32.383333 | 34.852778 |
| *Hordeum bulbosum* L. | 32.044547 | 36.65297 |
| *Hordeum bulbosum* L. | 31.16667 | 39.66667 |
| *Hordeum bulbosum* L. | 30.56398 | 50.41921 |
| *Hordeum bulbosum* L. | 34.989949 | 31.949801 |
| *Hordeum bulbosum* L. | 34.95389 | 32.503604 |
| *Hordeum bulbosum* L. | 33.2 | 35.06667 |
| *Hordeum bulbosum* L. | 32.95 | 39.916667 |
| *Hordeum bulbosum* L. | 33.792628 | 44.449543 |
| *Hordeum bulbosum* L. | 33.747542 | 45.096156 |
| *Hordeum bulbosum* L. | 35.138725 | 32.435141 |
| *Hordeum bulbosum* L. | 35.750733 | 33.221514 |
| *Hordeum bulbosum* L. | 36.565 | 35.73056 |
| *Hordeum bulbosum* L. | 35.33333 | 39.58333 |
| *Hordeum bulbosum* L. | 35.66222 | 40.97056 |
| *Hordeum bulbosum* L. | 35.134995 | 44.9547 |
| *Hordeum bulbosum* L. | 36.469044 | 45.351825 |
| *Hordeum bulbosum* L. | 38.333333 | 37.483333 |
| *Hordeum bulbosum* L. | 38.1 | 38.48333 |
| *Hordeum bulbosum* L. | 41.7833 | 36.3833 |
| *Hordeum bulbosum* L. | 40.398056 | 37.806111 |
| *Hordeum bulbosum* L. | 41.01916 | 43.003674 |
| *Hordeum bulbosum* L. | 44 | 33 |
| *Hordeum bulbosum* L. | 44.971681 | 36.562922 |
| *Hordeum bulbosum* L. | 44.88705 | 39.88168 |
| *Hordeum bulbosum* L. | 44.65 | 40.36667 |
| *Hordeum bulbosum* L. | 46.85 | 34.416667 |
| *Hordeum bulbosum* L. | 45.206676 | 35.983939 |
| *Hordeum bulbosum* L. | 45.23 | 39.93 |
| *Hordeum bulbosum* L. | 45 | 40.1 |
| *Hordeum bulbosum* L. | 47.471516 | 42.899897 |
| *Hordeum bulbosum* L. | 48.7 | 31.31667 |
| *Hordeum bulbosum* L. | 47.716667 | 33.1 |
| *Hordeum bulbosum* L. | 48.4528 | 38.8103 |
| *Hordeum bulbosum* L. | 48.277578 | 42.048293 |
| *Hordeum bulbosum* L. | 47.634324 | 42.888059 |
| *Hordeum bulbosum* L. | 52.43333 | 29.73333 |
| *Hordeum bulbosum* L. | 51.148708 | 31.346788 |
| *Hordeum bulbosum* L. | 50.98333 | 35.8 |
| *Hordeum bulbosum* L. | 51.033333 | 49.106082 |
| *Hordeum bulbosum* L. | 52.58667 | 29.72028 |
| *Hordeum bulbosum* L. | 53 | 30.0833 |
| *Hordeum bulbosum* L. | 56 | 37.35 |
| *Hordeum bulbosum* L. | 56.3836 | 38.4997 |
| *Hordeum bulbosum* L. | 57.5667 | 38.7 |
| *Hordeum bulbosum* L. | 6.86667 | 36.2333 |
| *Hordeum bulbosum* L. | 5.42045 | 43.29169 |
| *Hordeum bulbosum* L. | 7.44504 | 46.95288 |
| *Hordeum bulbosum* L. | 6.077055 | 50.75558 |
| *Hordeum bulbosum* L. | 60 | 40 |
| *Hordeum bulbosum* L. | 64.3 | 31.66667 |
| *Hordeum bulbosum* L. | 63.11667 | 34.98333 |
| *Hordeum bulbosum* L. | 64.43333 | 35.83333 |
| *Hordeum bulbosum* L. | 67.083333 | 36.716667 |
| *Hordeum bulbosum* L. | 67.20191 | 38.206791 |
| *Hordeum bulbosum* L. | 67.68333 | 36.68333 |
| *Hordeum bulbosum* L. | 69.40556 | 38.13556 |
| *Hordeum bulbosum* L. | 69.598897 | 42.30379 |
| *Hordeum bulbosum* L. | 69.89257 | 42.798058 |
| *Hordeum bulbosum* L. | 7.83333 | 36.3333 |
| *Hordeum bulbosum* L. | 8.573166 | 39.562879 |
| *Hordeum bulbosum* L. | 9.06667 | 40.03333 |
| *Hordeum bulbosum* L. | 9.47799 | 42.77772 |
| *Hordeum bulbosum* L. | 7.531023 | 47.202903 |
| *Hordeum bulbosum* L. | 7.766627 | 48.58363 |
| *Hordeum bulbosum* L. | 71.05 | 34.2 |
| *Hordeum bulbosum* L. | 70.57972 | 37.1175 |
| *Hordeum bulbosum* L. | 70.04111 | 38.42583 |
| *Hordeum bulbosum* L. | 70.119927 | 41.147965 |
| *Hordeum bulbosum* L. | 70.9965 | 42.765 |
| *Hordeum bulbosum* L. | 73.821986 | 39.6407 |
| *Hordeum bulbosum* L. | 73.427635 | 40.306732 |
| *Hordeum bulbosum* L. | 73.51379 | 42.852157 |
| *Hordeum bulbosum* L. | 94.9 | 36.42 |
| *Hordeum californicum* Covas & Stebbins | -112.1499193 | 36.5186842 |
| *Hordeum californicum* Covas & Stebbins | -116.572 | 32.9718 |
| *Hordeum californicum* Covas & Stebbins | -116.825928 | 33.319978 |
| *Hordeum californicum* Covas & Stebbins | -116.911422 | 34.243896 |
| *Hordeum californicum* Covas & Stebbins | -117.208333 | 32.708333 |
| *Hordeum californicum* Covas & Stebbins | -117.248703 | 33.529542 |
| *Hordeum californicum* Covas & Stebbins | -117.966256 | 34.347636 |
| *Hordeum californicum* Covas & Stebbins | -117.387951 | 35.853676 |
| *Hordeum californicum* Covas & Stebbins | -117.9164 | 45.660829 |
| *Hordeum californicum* Covas & Stebbins | -117.001189 | 46.400409 |
| *Hordeum californicum* Covas & Stebbins | -118.41667 | 33.38333 |
| *Hordeum californicum* Covas & Stebbins | -118.9886 | 34.0808 |
| *Hordeum californicum* Covas & Stebbins | -118.6529673 | 35.7407184 |
| *Hordeum californicum* Covas & Stebbins | -118.8108863 | 36.2249423 |
| *Hordeum californicum* Covas & Stebbins | -118.14443 | 37.50175 |
| *Hordeum californicum* Covas & Stebbins | -119.5484 | 33.2789 |
| *Hordeum californicum* Covas & Stebbins | -119.69819 | 34.42083 |
| *Hordeum californicum* Covas & Stebbins | -119.868105 | 35.542196 |
| *Hordeum californicum* Covas & Stebbins | -119.420833 | 36.963889 |
| *Hordeum californicum* Covas & Stebbins | -119.85472 | 37.88222 |
| *Hordeum californicum* Covas & Stebbins | -119.6445813 | 38.3536112 |
| *Hordeum californicum* Covas & Stebbins | -119.3414 | 45.9175 |
| *Hordeum californicum* Covas & Stebbins | -120.04146 | 33.99327 |
| *Hordeum californicum* Covas & Stebbins | -120.1987864 | 34.5265552 |
| *Hordeum californicum* Covas & Stebbins | -120.261244 | 35.294975 |
| *Hordeum californicum* Covas & Stebbins | -120.456458 | 37.857818 |
| *Hordeum californicum* Covas & Stebbins | -120.6230943 | 38.7460955 |
| *Hordeum californicum* Covas & Stebbins | -120.353982 | 39.345451 |
| *Hordeum californicum* Covas & Stebbins | -120.237776 | 41.316851 |
| *Hordeum californicum* Covas & Stebbins | -120.15024 | 42.3881 |
| *Hordeum californicum* Covas & Stebbins | -121.204 | 35.861 |
| *Hordeum californicum* Covas & Stebbins | -121.6101287 | 36.9109274 |
| *Hordeum californicum* Covas & Stebbins | -121.97954 | 37.087204 |
| *Hordeum californicum* Covas & Stebbins | -121.826675 | 38.273079 |
| *Hordeum californicum* Covas & Stebbins | -121.9794 | 39.16481 |
| *Hordeum californicum* Covas & Stebbins | -121.1371891 | 40.7356954 |
| *Hordeum californicum* Covas & Stebbins | -121.7806 | 42.225 |
| *Hordeum californicum* Covas & Stebbins | -121.20999 | 43.728158 |
| *Hordeum californicum* Covas & Stebbins | -122.05806 | 36.99556 |
| *Hordeum californicum* Covas & Stebbins | -122.35372 | 37.52282 |
| *Hordeum californicum* Covas & Stebbins | -122.712178 | 38.062824 |
| *Hordeum californicum* Covas & Stebbins | -122.641667 | 39.898611 |
| *Hordeum californicum* Covas & Stebbins | -122.5052333 | 41.5150715 |
| *Hordeum californicum* Covas & Stebbins | -122.1422 | 42.8681 |
| *Hordeum californicum* Covas & Stebbins | -122.13147 | 43.07657 |
| *Hordeum californicum* Covas & Stebbins | -123.72607 | 38.93809 |
| *Hordeum californicum* Covas & Stebbins | -123.2064144 | 39.1445931 |
| *Hordeum californicum* Covas & Stebbins | -123.7768598 | 40.0017691 |
| *Hordeum californicum* Covas & Stebbins | -123.545278 | 41.933056 |
| *Hordeum californicum* Covas & Stebbins | -123.637278 | 42.249 |
| *Hordeum californicum* Covas & Stebbins | -124.1428644 | 41.0597638 |
| *Hordeum californicum* Covas & Stebbins | -149.95 | 60.48333 |
| *Hordeum californicum* Covas & Stebbins | 139.453049 | 35.507417 |
| *Hordeum chilense* Roem. & Schult. | -119.857669 | 34.424334 |
| *Hordeum chilense* Roem. & Schult. | -1.705289 | 53.699609 |
| *Hordeum chilense* Roem. & Schult. | -64 | -21 |
| *Hordeum chilense* Roem. & Schult. | -64.670931 | -16.714611 |
| *Hordeum chilense* Roem. & Schult. | -65.29 | -23.54 |
| *Hordeum chilense* Roem. & Schult. | -68.898889 | -32.883889 |
| *Hordeum chilense* Roem. & Schult. | -69.250278 | -32.408056 |
| *Hordeum chilense* Roem. & Schult. | -69.094444 | -34.047222 |
| *Hordeum chilense* Roem. & Schult. | -71 | -30 |
| *Hordeum chilense* Roem. & Schult. | -71 | -32 |
| *Hordeum chilense* Roem. & Schult. | -70.766667 | -32.35 |
| *Hordeum chilense* Roem. & Schult. | -70.5 | -33.59 |
| *Hordeum chilense* Roem. & Schult. | -70.9833 | -34.6667 |
| *Hordeum chilense* Roem. & Schult. | -70.866667 | -35.2 |
| *Hordeum chilense* Roem. & Schult. | -70.966667 | -36.266667 |
| *Hordeum chilense* Roem. & Schult. | -70.1 | -38.9 |
| *Hordeum chilense* Roem. & Schult. | -71.3 | -30 |
| *Hordeum chilense* Roem. & Schult. | -71.308333 | -30.008333 |
| *Hordeum chilense* Roem. & Schult. | -71.416667 | -31.5 |
| *Hordeum chilense* Roem. & Schult. | -71.51611 | -32.92194 |
| *Hordeum chilense* Roem. & Schult. | -71.0333 | -33.0667 |
| *Hordeum chilense* Roem. & Schult. | -71.1 | -34.033333 |
| *Hordeum chilense* Roem. & Schult. | -71.5833 | -35.85 |
| *Hordeum chilense* Roem. & Schult. | -71.016667 | -36.666667 |
| *Hordeum chilense* Roem. & Schult. | -71.37389 | -37.8525 |
| *Hordeum chilense* Roem. & Schult. | -71.09 | -39.11 |
| *Hordeum chilense* Roem. & Schult. | -71.316667 | -41.133333 |
| *Hordeum chilense* Roem. & Schult. | -71.716667 | -46.533333 |
| *Hordeum chilense* Roem. & Schult. | -72.4333 | -35 |
| *Hordeum chilense* Roem. & Schult. | -72.016667 | -35.066667 |
| *Hordeum chilense* Roem. & Schult. | -72.94 | -36.56 |
| *Hordeum chilense* Roem. & Schult. | -72.716667 | -39.016667 |
| *Hordeum chilense* Roem. & Schult. | -73.19 | -36.76 |
| *Hordeum chilense* Roem. & Schult. | -73.38 | -39.85 |
| *Hordeum chilense* Roem. & Schult. | 4.802 | 52.50283 |
| *Hordeum marinum* Huds. | -101.467183 | 31.191331 |
| *Hordeum marinum* Huds. | -103.4 | 33.92 |
| *Hordeum marinum* Huds. | -106.63739 | 32.00189 |
| *Hordeum marinum* Huds. | -105.83897 | 46.400991 |
| *Hordeum marinum* Huds. | -9.920055 | 30.702342 |
| *Hordeum marinum* Huds. | -7.885447 | 37.022727 |
| *Hordeum marinum* Huds. | -8.9 | 38.77 |
| *Hordeum marinum* Huds. | -8.42248 | 40.205091 |
| *Hordeum marinum* Huds. | -8.014 | 42.912 |
| *Hordeum marinum* Huds. | -108.8175 | 37.341389 |
| *Hordeum marinum* Huds. | -109.572267 | 38.070952 |
| *Hordeum marinum* Huds. | -109.528747 | 40.46196 |
| *Hordeum marinum* Huds. | -112.173917 | 33.528739 |
| *Hordeum marinum* Huds. | -111.644678 | 36.800199 |
| *Hordeum marinum* Huds. | -111.83624 | 39.69394 |
| *Hordeum marinum* Huds. | -111.471111 | 40.106944 |
| *Hordeum marinum* Huds. | -113.209 | 34.3547 |
| *Hordeum marinum* Huds. | -114.625978 | 35.233908 |
| *Hordeum marinum* Huds. | -112.63785 | 40.75655 |
| *Hordeum marinum* Huds. | -114.9339673 | 43.0462144 |
| *Hordeum marinum* Huds. | -114.47157 | 47.46721 |
| *Hordeum marinum* Huds. | -116.308442 | 31.017745 |
| *Hordeum marinum* Huds. | -117.12795 | 32.81038 |
| *Hordeum marinum* Huds. | -115.523333 | 41.065 |
| *Hordeum marinum* Huds. | -116.71878 | 43.933909 |
| *Hordeum marinum* Huds. | -116.99917 | 46.775931 |
| *Hordeum marinum* Huds. | -116.780466 | 47.677683 |
| *Hordeum marinum* Huds. | -118.039581 | 33.794905 |
| *Hordeum marinum* Huds. | -119.73516 | 37.109227 |
| *Hordeum marinum* Huds. | -119.79849 | 37.93799 |
| *Hordeum marinum* Huds. | -119.56 | 40.92 |
| *Hordeum marinum* Huds. | -119.5347 | 44.4683 |
| *Hordeum marinum* Huds. | -119.399286 | 45.4029 |
| *Hordeum marinum* Huds. | -118.51861 | 47.75861 |
| *Hordeum marinum* Huds. | -120.04164 | 34.6925 |
| *Hordeum marinum* Huds. | -121.740128 | 36.82404 |
| *Hordeum marinum* Huds. | -122.434271 | 37.737084 |
| *Hordeum marinum* Huds. | -122.2449134 | 40.9401706 |
| *Hordeum marinum* Huds. | -121.292963 | 44.360386 |
| *Hordeum marinum* Huds. | -120.463362 | 45.13085 |
| *Hordeum marinum* Huds. | -122.33083 | 47.60639 |
| *Hordeum marinum* Huds. | -122.689178 | 38.569845 |
| *Hordeum marinum* Huds. | -123.408681 | 41.336132 |
| *Hordeum marinum* Huds. | -123.26327 | 44.55779 |
| *Hordeum marinum* Huds. | -122.63806 | 45.44639 |
| *Hordeum marinum* Huds. | -123.520522 | 48.38715 |
| *Hordeum marinum* Huds. | -13.8212044 | 28.788556 |
| *Hordeum marinum* Huds. | -15.631788 | 28.139388 |
| *Hordeum marinum* Huds. | -17.008333 | 32.708333 |
| *Hordeum marinum* Huds. | -2.195 | 34.2264 |
| *Hordeum marinum* Huds. | -2.1 | 36.86 |
| *Hordeum marinum* Huds. | -1.57 | 38.830002 |
| *Hordeum marinum* Huds. | -1.72 | 40.28 |
| *Hordeum marinum* Huds. | -1.8612176 | 42.8288892 |
| *Hordeum marinum* Huds. | -2.460715 | 47.302998 |
| *Hordeum marinum* Huds. | -2.488901 | 47.516963 |
| *Hordeum marinum* Huds. | -0.054443 | 51.547751 |
| *Hordeum marinum* Huds. | -0.335237 | 52.541141 |
| *Hordeum marinum* Huds. | -1.620715 | 55.514123 |
| *Hordeum marinum* Huds. | -21.383 | 64.75 |
| *Hordeum marinum* Huds. | -25.169466 | 36.957482 |
| *Hordeum marinum* Huds. | -2.72694 | 34.5719 |
| *Hordeum marinum* Huds. | -3.22 | 37.4 |
| *Hordeum marinum* Huds. | -2.82 | 37.92 |
| *Hordeum marinum* Huds. | -3.72 | 40.44 |
| *Hordeum marinum* Huds. | -4.951 | 42.828 |
| *Hordeum marinum* Huds. | -2.52369 | 47.29234 |
| *Hordeum marinum* Huds. | -2.709812 | 47.618292 |
| *Hordeum marinum* Huds. | -3.63612 | 50.299552 |
| *Hordeum marinum* Huds. | -3.126067 | 53.41364 |
| *Hordeum marinum* Huds. | -3.1678 | 55.946701 |
| *Hordeum marinum* Huds. | -3.633711 | 57.614905 |
| *Hordeum marinum* Huds. | -64 | -35 |
| *Hordeum marinum* Huds. | -64 | -38 |
| *Hordeum marinum* Huds. | -6.33333 | 33.6833 |
| *Hordeum marinum* Huds. | -6.51 | 36.99 |
| *Hordeum marinum* Huds. | -6.05 | 38.03 |
| *Hordeum marinum* Huds. | -5.14 | 40.37 |
| *Hordeum marinum* Huds. | -5.75 | 42.82 |
| *Hordeum marinum* Huds. | -5.266622 | 50.205044 |
| *Hordeum marinum* Huds. | -69.8 | -36.4 |
| *Hordeum marinum* Huds. | -71 | -30 |
| *Hordeum marinum* Huds. | -71.55 | -31.55 |
| *Hordeum marinum* Huds. | -71.716667 | -34.383333 |
| *Hordeum marinum* Huds. | -71.03 | -36.83 |
| *Hordeum marinum* Huds. | -70.45 | -39.43 |
| *Hordeum marinum* Huds. | -71.49 | -43.02 |
| *Hordeum marinum* Huds. | -70.92666 | -47.44472 |
| *Hordeum marinum* Huds. | -74.85198 | 40.10084 |
| *Hordeum marinum* Huds. | -77.0283 | 38.899446 |
| *Hordeum marinum* Huds. | -79.893677 | -26.347778 |
| *Hordeum marinum* Huds. | -83.107889 | 41.506711 |
| *Hordeum marinum* Huds. | -87.971793 | 41.917846 |
| *Hordeum marinum* Huds. | -93.466095 | 42.037537 |
| *Hordeum marinum* Huds. | 2.3 | 34.8333 |
| *Hordeum marinum* Huds. | 0.19 | 38.77 |
| *Hordeum marinum* Huds. | 0.12 | 40.12 |
| *Hordeum marinum* Huds. | 2.44654 | 44.93436 |
| *Hordeum marinum* Huds. | 0.35957 | 46.58365 |
| *Hordeum marinum* Huds. | 2.320781 | 48.951745 |
| *Hordeum marinum* Huds. | 1.002222 | 51.794722 |
| *Hordeum marinum* Huds. | 1.103093 | 52.953269 |
| *Hordeum marinum* Huds. | 10.1 | 33.8863 |
| *Hordeum marinum* Huds. | 10.05 | 36.283333 |
| *Hordeum marinum* Huds. | 12.463242 | 37.908974 |
| *Hordeum marinum* Huds. | 11.217517 | 42.464812 |
| *Hordeum marinum* Huds. | 10.28333 | 43.71667 |
| *Hordeum marinum* Huds. | 10.040859 | 45.070042 |
| *Hordeum marinum* Huds. | 10.96 | 48.27 |
| *Hordeum marinum* Huds. | 12.291566 | 51.374957 |
| *Hordeum marinum* Huds. | 10.75109 | 53.067252 |
| *Hordeum marinum* Huds. | 12.339253 | 55.830724 |
| *Hordeum marinum* Huds. | 11.93079 | 57.69181 |
| *Hordeum marinum* Huds. | 114.838149 | -28.648805 |
| *Hordeum marinum* Huds. | 116.258503 | -29.251861 |
| *Hordeum marinum* Huds. | 116.019722 | -30.569444 |
| *Hordeum marinum* Huds. | 115.558333 | -33.553333 |
| *Hordeum marinum* Huds. | 118.157 | -32.1035 |
| *Hordeum marinum* Huds. | 118.446389 | -33.411389 |
| *Hordeum marinum* Huds. | 14.281115 | 36.061969 |
| *Hordeum marinum* Huds. | 14.99496 | 37.73386 |
| *Hordeum marinum* Huds. | 12.91 | 41.4 |
| *Hordeum marinum* Huds. | 13.844667 | 44.828122 |
| *Hordeum marinum* Huds. | 14.040328 | 45.039979 |
| *Hordeum marinum* Huds. | 13.561153 | 49.749408 |
| *Hordeum marinum* Huds. | 14.410891 | 50.068232 |
| *Hordeum marinum* Huds. | 13.13005 | 57.45895 |
| *Hordeum marinum* Huds. | 120.1267 | -33.949285 |
| *Hordeum marinum* Huds. | 127.7571 | 26.1827 |
| *Hordeum marinum* Huds. | 130.416667 | -30.616667 |
| *Hordeum marinum* Huds. | 133.833333 | -23.833333 |
| *Hordeum marinum* Huds. | 134.885278 | -33.559444 |
| *Hordeum marinum* Huds. | 136.81302 | -31.165387 |
| *Hordeum marinum* Huds. | 135.550278 | -34.584722 |
| *Hordeum marinum* Huds. | 136.90524 | -35.91136 |
| *Hordeum marinum* Huds. | 135.16667 | 34.65 |
| *Hordeum marinum* Huds. | 137.73381 | -32.48711 |
| *Hordeum marinum* Huds. | 138.615244 | -34.935627 |
| *Hordeum marinum* Huds. | 138.47011 | -35.15516 |
| *Hordeum marinum* Huds. | 138.44705 | 35.70887 |
| *Hordeum marinum* Huds. | 140.7805 | -34.16615 |
| *Hordeum marinum* Huds. | 142.15372 | -37.33267 |
| *Hordeum marinum* Huds. | 142.08468 | -37.748521 |
| *Hordeum marinum* Huds. | 143.534471 | -34.214669 |
| *Hordeum marinum* Huds. | 143.76391 | -35.78429 |
| *Hordeum marinum* Huds. | 144.437567 | -38.249303 |
| *Hordeum marinum* Huds. | 143.904861 | -40.129444 |
| *Hordeum marinum* Huds. | 146.04377 | -34.660551 |
| *Hordeum marinum* Huds. | 145.08333 | -36.51913 |
| *Hordeum marinum* Huds. | 145.381269 | -38.103134 |
| *Hordeum marinum* Huds. | 147.131425 | -41.615964 |
| *Hordeum marinum* Huds. | 147.433333 | -42.833333 |
| *Hordeum marinum* Huds. | 147.980935 | -31.794084 |
| *Hordeum marinum* Huds. | 148.681128 | -34.850473 |
| *Hordeum marinum* Huds. | 149.19974 | -36.30916 |
| *Hordeum marinum* Huds. | 147.501282 | -38.498474 |
| *Hordeum marinum* Huds. | 147.976057 | -40.1859 |
| *Hordeum marinum* Huds. | 147.526367 | -42.740214 |
| *Hordeum marinum* Huds. | 151.917747 | -26.581748 |
| *Hordeum marinum* Huds. | 151.716667 | -29.716667 |
| *Hordeum marinum* Huds. | 151.54722 | -30.84361 |
| *Hordeum marinum* Huds. | 151.084491 | -33.91509 |
| *Hordeum marinum* Huds. | 153.109394 | -27.390069 |
| *Hordeum marinum* Huds. | 15.019455 | 37.137716 |
| *Hordeum marinum* Huds. | 15.241747 | 38.242811 |
| *Hordeum marinum* Huds. | 17.38377 | 40.89409 |
| *Hordeum marinum* Huds. | 15.202661 | 44.246839 |
| *Hordeum marinum* Huds. | 16.975989 | 47.107414 |
| *Hordeum marinum* Huds. | 16.38214 | 48.21728 |
| *Hordeum marinum* Huds. | 17.35621 | 62.49306 |
| *Hordeum marinum* Huds. | 169.913674 | -45.283934 |
| *Hordeum marinum* Huds. | 18.892174 | -32.181734 |
| *Hordeum marinum* Huds. | 18.471803 | -33.709477 |
| *Hordeum marinum* Huds. | 19.9 | 39.616667 |
| *Hordeum marinum* Huds. | 19.4957 | 42.05415 |
| *Hordeum marinum* Huds. | 17.842985 | 42.719911 |
| *Hordeum marinum* Huds. | 19.090078 | 47.144577 |
| *Hordeum marinum* Huds. | 19.228468 | 47.705083 |
| *Hordeum marinum* Huds. | 18.4788 | 54.4996 |
| *Hordeum marinum* Huds. | 18.03642 | 59.33229 |
| *Hordeum marinum* Huds. | 172.441345 | -43.728816 |
| *Hordeum marinum* Huds. | 170.087397 | -45.575967 |
| *Hordeum marinum* Huds. | 174.588333 | -38.813611 |
| *Hordeum marinum* Huds. | 174.142002 | -41.727906 |
| *Hordeum marinum* Huds. | 172.687013 | -43.744137 |
| *Hordeum marinum* Huds. | 175.9996207 | -39.4981959 |
| *Hordeum marinum* Huds. | 175.006884 | -40.8739435 |
| *Hordeum marinum* Huds. | 177.649437 | -37.803908 |
| *Hordeum marinum* Huds. | 3.8 | 34.85 |
| *Hordeum marinum* Huds. | 3.71667 | 35.9333 |
| *Hordeum marinum* Huds. | 2.6225 | 39.711667 |
| *Hordeum marinum* Huds. | 4.0965 | 40.0373 |
| *Hordeum marinum* Huds. | 4.401047 | 43.472214 |
| *Hordeum marinum* Huds. | 2.9 | 45.7 |
| *Hordeum marinum* Huds. | 2.90812 | 51.24647 |
| *Hordeum marinum* Huds. | 4.610229 | 52.554123 |
| *Hordeum marinum* Huds. | 20.430142 | -32.470581 |
| *Hordeum marinum* Huds. | 20.447748 | -32.539762 |
| *Hordeum marinum* Huds. | 21.23124 | 32 |
| *Hordeum marinum* Huds. | 22.161111 | 37.230556 |
| *Hordeum marinum* Huds. | 21.22 | 39.79 |
| *Hordeum marinum* Huds. | 22.033333 | 40.9 |
| *Hordeum marinum* Huds. | 21.9 | 42.553333 |
| *Hordeum marinum* Huds. | 21.445958 | 46.500307 |
| *Hordeum marinum* Huds. | 20.369122 | 47.522768 |
| *Hordeum marinum* Huds. | 22.128139 | 60.488096 |
| *Hordeum marinum* Huds. | 21.634973 | 63.069125 |
| *Hordeum marinum* Huds. | 24.066667 | 35.5 |
| *Hordeum marinum* Huds. | 24.95 | 37.866667 |
| *Hordeum marinum* Huds. | 22.583333 | 40.466667 |
| *Hordeum marinum* Huds. | 23.2286 | 42.6543 |
| *Hordeum marinum* Huds. | 23.906558 | 46.910817 |
| *Hordeum marinum* Huds. | 23.830407 | 61.63199 |
| *Hordeum marinum* Huds. | 25.4833 | 29.2 |
| *Hordeum marinum* Huds. | 27.2167 | 31.3 |
| *Hordeum marinum* Huds. | 26.266667 | 35.25 |
| *Hordeum marinum* Huds. | 25.116667 | 39.85 |
| *Hordeum marinum* Huds. | 26.15 | 41.666667 |
| *Hordeum marinum* Huds. | 27.47833 | 42.51694 |
| *Hordeum marinum* Huds. | 27.280731 | 45.225174 |
| *Hordeum marinum* Huds. | 26.75 | 58.35 |
| *Hordeum marinum* Huds. | 25.018642 | 60.110161 |
| *Hordeum marinum* Huds. | 29.95 | 31.1667 |
| *Hordeum marinum* Huds. | 27.766667 | 35.933333 |
| *Hordeum marinum* Huds. | 27.508333 | 37.808333 |
| *Hordeum marinum* Huds. | 27.858556 | 42.159491 |
| *Hordeum marinum* Huds. | 28.62861 | 44.21778 |
| *Hordeum marinum* Huds. | 28.031111 | 45.425833 |
| *Hordeum marinum* Huds. | 31.280001 | 40.580002 |
| *Hordeum marinum* Huds. | 32.080407 | 47.108615 |
| *Hordeum marinum* Huds. | 34.98986 | 31.727887 |
| *Hordeum marinum* Huds. | 34.961993 | 32.501726 |
| *Hordeum marinum* Huds. | 33.0667 | 35.3 |
| *Hordeum marinum* Huds. | 34.9 | 39 |
| *Hordeum marinum* Huds. | 32.908333 | 40.108333 |
| *Hordeum marinum* Huds. | 33.729118 | 44.440797 |
| *Hordeum marinum* Huds. | 33.044967 | 45.295703 |
| *Hordeum marinum* Huds. | 35.164967 | 31.79414 |
| *Hordeum marinum* Huds. | 35.694567 | 33.017347 |
| *Hordeum marinum* Huds. | 36.53056 | 35.68694 |
| *Hordeum marinum* Huds. | 37.41667 | 39.5 |
| *Hordeum marinum* Huds. | 37.466492 | 44.706524 |
| *Hordeum marinum* Huds. | 37.116265 | 45.046208 |
| *Hordeum marinum* Huds. | 39.989444 | 37.220278 |
| *Hordeum marinum* Huds. | 39.239161 | 43.979156 |
| *Hordeum marinum* Huds. | 38.63057 | 45.078483 |
| *Hordeum marinum* Huds. | 41.208333 | 39.908333 |
| *Hordeum marinum* Huds. | 41.0102 | 57.061 |
| *Hordeum marinum* Huds. | 44.1 | 32.8 |
| *Hordeum marinum* Huds. | 44.608333 | 39.208333 |
| *Hordeum marinum* Huds. | 44.543607 | 40.90485 |
| *Hordeum marinum* Huds. | 42.953492 | 44.175907 |
| *Hordeum marinum* Huds. | 47 | 35.31667 |
| *Hordeum marinum* Huds. | 46.116389 | 39.498056 |
| *Hordeum marinum* Huds. | 45.180209 | 41.289304 |
| *Hordeum marinum* Huds. | 45.9353 | 44.5067 |
| *Hordeum marinum* Huds. | 49.61667 | 30.73333 |
| *Hordeum marinum* Huds. | 48.508333 | 34.908333 |
| *Hordeum marinum* Huds. | 49.901667 | 36.934444 |
| *Hordeum marinum* Huds. | 48.908333 | 38.408333 |
| *Hordeum marinum* Huds. | 47.7965 | 42.4657 |
| *Hordeum marinum* Huds. | 47.661533 | 42.822756 |
| *Hordeum marinum* Huds. | 51.885464 | 32.106569 |
| *Hordeum marinum* Huds. | 51.708333 | 35.908333 |
| *Hordeum marinum* Huds. | 52.53333 | 29.6 |
| *Hordeum marinum* Huds. | 53 | 32 |
| *Hordeum marinum* Huds. | 57.1267 | 37.5272 |
| *Hordeum marinum* Huds. | 5.96667 | 35.4333 |
| *Hordeum marinum* Huds. | 6.736945 | 43.415194 |
| *Hordeum marinum* Huds. | 5.921405 | 45.559947 |
| *Hordeum marinum* Huds. | 7.256 | 47.903 |
| *Hordeum marinum* Huds. | 5.09 | 51.56 |
| *Hordeum marinum* Huds. | 5.95 | 53.35 |
| *Hordeum marinum* Huds. | 65.75361 | 36.66722 |
| *Hordeum marinum* Huds. | 67.20191 | 38.206791 |
| *Hordeum marinum* Huds. | 67.7 | 33.9 |
| *Hordeum marinum* Huds. | 68.708333 | 38.508333 |
| *Hordeum marinum* Huds. | 68.787207 | 40.80756 |
| *Hordeum marinum* Huds. | 8.1333 | 33.9183 |
| *Hordeum marinum* Huds. | 7.83333 | 36.3333 |
| *Hordeum marinum* Huds. | 9.153659 | 39.224138 |
| *Hordeum marinum* Huds. | 9.413406 | 41.959817 |
| *Hordeum marinum* Huds. | 9.42606 | 42.97212 |
| *Hordeum marinum* Huds. | 7.511256 | 47.225404 |
| *Hordeum marinum* Huds. | 9.9 | 47.95 |
| *Hordeum marinum* Huds. | 7.582427 | 51.934589 |
| *Hordeum marinum* Huds. | 8.869722 | 54.935278 |
| *Hordeum marinum* Huds. | 8.761944 | 55.328333 |
| *Hordeum marinum* Huds. | 7.980594 | 58.154391 |
| *Hordeum marinum* Huds. | 71.565588 | 40.403767 |
| *Hordeum marinum* Huds. | 70.962278 | 51.995778 |
| *Hordeum marinum* Huds. | 94.9 | 36.42 |
| *Hordeum marinum* Huds. | 98.08 | 36.3 |
| *Hordeum murinum* L. | -98.888333 | 19.491667 |
| *Hordeum murinum* L. | -98.272066 | 29.954478 |
| *Hordeum murinum* L. | -99.029102 | 31.341428 |
| *Hordeum murinum* L. | -98.738673 | 33.194 |
| *Hordeum murinum* L. | -101.850262 | 21.879233 |
| *Hordeum murinum* L. | -101.826389 | 29.903611 |
| *Hordeum murinum* L. | -100.47309 | 31.45852 |
| *Hordeum murinum* L. | -101.919242 | 34.979912 |
| *Hordeum murinum* L. | -101.92958 | 35.13798 |
| *Hordeum murinum* L. | -103.55833 | 31.86667 |
| *Hordeum murinum* L. | -103.199047 | 34.410747 |
| *Hordeum murinum* L. | -104.615278 | 35.595556 |
| *Hordeum murinum* L. | -104.91708 | 39.45573 |
| *Hordeum murinum* L. | -104.709132 | 40.423284 |
| *Hordeum murinum* L. | -103.00389 | 42.81772 |
| *Hordeum murinum* L. | -106.461667 | 31.913889 |
| *Hordeum murinum* L. | -105.819667 | 32.954117 |
| *Hordeum murinum* L. | -106.626595 | 35.078289 |
| *Hordeum murinum* L. | -105.183345 | 39.746447 |
| *Hordeum murinum* L. | -105.071525 | 40.573097 |
| *Hordeum murinum* L. | -106.306051 | 42.825512 |
| *Hordeum murinum* L. | -7.866667 | 31.2 |
| *Hordeum murinum* L. | -7.539944 | 33.566221 |
| *Hordeum murinum* L. | -7.821226 | 37.0887128 |
| *Hordeum murinum* L. | -8 | 39.5 |
| *Hordeum murinum* L. | -8.42248 | 40.205091 |
| *Hordeum murinum* L. | -8.397359 | 43.386768 |
| *Hordeum murinum* L. | -8.49231 | 51.883056 |
| *Hordeum murinum* L. | -8.196359 | 52.947914 |
| *Hordeum murinum* L. | -108.9259382 | 28.371316 |
| *Hordeum murinum* L. | -108.70869 | 32.382375 |
| *Hordeum murinum* L. | -108.964041 | 33.819275 |
| *Hordeum murinum* L. | -108.85 | 37.33 |
| *Hordeum murinum* L. | -108.2573 | 38.7584 |
| *Hordeum murinum* L. | -109.52936 | 40.46175 |
| *Hordeum murinum* L. | -109.010472 | 46.078138 |
| *Hordeum murinum* L. | -111.26667 | 26.11667 |
| *Hordeum murinum* L. | -110.281 | 31.3439 |
| *Hordeum murinum* L. | -111.73333 | 33.08333 |
| *Hordeum murinum* L. | -112.386867 | 37.203137 |
| *Hordeum murinum* L. | -112.392433 | 38.969482 |
| *Hordeum murinum* L. | -111.81667 | 41.73333 |
| *Hordeum murinum* L. | -112.44472 | 42.87139 |
| *Hordeum murinum* L. | -114.341667 | 29.358333 |
| *Hordeum murinum* L. | -114.7625 | 32.476669 |
| *Hordeum murinum* L. | -114.773333 | 34.891667 |
| *Hordeum murinum* L. | -113.606111 | 37.046667 |
| *Hordeum murinum* L. | -112.596585 | 38.70401 |
| *Hordeum murinum* L. | -113.88111 | 42.24333 |
| *Hordeum murinum* L. | -114.4731486 | 42.5705232 |
| *Hordeum murinum* L. | -114.33837 | 45.30695 |
| *Hordeum murinum* L. | -115.2346 | 28.1156 |
| *Hordeum murinum* L. | -115.616667 | 31.016667 |
| *Hordeum murinum* L. | -117.28333 | 33 |
| *Hordeum murinum* L. | -115.403671 | 35.116959 |
| *Hordeum murinum* L. | -116.599 | 39.8915 |
| *Hordeum murinum* L. | -115.776111 | 40.821111 |
| *Hordeum murinum* L. | -116.233333 | 43.666667 |
| *Hordeum murinum* L. | -117.17861 | 46.73139 |
| *Hordeum murinum* L. | -117.133333 | 49.55 |
| *Hordeum murinum* L. | -115.85 | 50.216667 |
| *Hordeum murinum* L. | -12.29103 | -37.11252 |
| *Hordeum murinum* L. | -10.0863 | 29.1428 |
| *Hordeum murinum* L. | -118.266667 | 29 |
| *Hordeum murinum* L. | -119.0357 | 33.4763 |
| *Hordeum murinum* L. | -119 | 37 |
| *Hordeum murinum* L. | -118.25 | 37.5 |
| *Hordeum murinum* L. | -119.663269 | 40.73132 |
| *Hordeum murinum* L. | -119.6435 | 44.5275 |
| *Hordeum murinum* L. | -117.81667 | 46.53333 |
| *Hordeum murinum* L. | -118.19556 | 48.586398 |
| *Hordeum murinum* L. | -118.666667 | 52.8 |
| *Hordeum murinum* L. | -120.06208 | 34.533539 |
| *Hordeum murinum* L. | -120.319167 | 36.2575 |
| *Hordeum murinum* L. | -121.78333 | 38.6 |
| *Hordeum murinum* L. | -122.074097 | 40.368401 |
| *Hordeum murinum* L. | -121.2569 | 44.2772 |
| *Hordeum murinum* L. | -120.73333 | 45.48333 |
| *Hordeum murinum* L. | -122.33083 | 47.60639 |
| *Hordeum murinum* L. | -120.866667 | 50.766667 |
| *Hordeum murinum* L. | -122.59169 | 37.9273 |
| *Hordeum murinum* L. | -124.3214 | 42.0658 |
| *Hordeum murinum* L. | -123.0339 | 44.9431 |
| *Hordeum murinum* L. | -122.6674 | 45.5407 |
| *Hordeum murinum* L. | -124.641 | 49.42025 |
| *Hordeum murinum* L. | -125.000035 | 49.952645 |
| *Hordeum murinum* L. | -125.184167 | 50.094722 |
| *Hordeum murinum* L. | -155.683 | 19.9167 |
| *Hordeum murinum* L. | -156.456443 | 20.868239 |
| *Hordeum murinum* L. | -13.862386 | 28.724651 |
| *Hordeum murinum* L. | -165.9333 | 54.1667 |
| *Hordeum murinum* L. | -15.6 | 27.9 |
| *Hordeum murinum* L. | -15.8812515 | 30.1385461 |
| *Hordeum murinum* L. | -16.347778 | 33.085556 |
| *Hordeum murinum* L. | -176.179602 | -44.345562 |
| *Hordeum murinum* L. | -1.7310461 | 32.2187033 |
| *Hordeum murinum* L. | -2.29333 | 33.9856 |
| *Hordeum murinum* L. | -2.38 | 37.04 |
| *Hordeum murinum* L. | -0.482492 | 38.349388 |
| *Hordeum murinum* L. | -1.368655 | 41.893049 |
| *Hordeum murinum* L. | -0.883614 | 42.591152 |
| *Hordeum murinum* L. | -2.17307 | 47.22854 |
| *Hordeum murinum* L. | -0.336647 | 49.153744 |
| *Hordeum murinum* L. | -1.1702 | 50.779999 |
| *Hordeum murinum* L. | -1.190742 | 54.114151 |
| *Hordeum murinum* L. | -1.633834 | 55.037465 |
| *Hordeum murinum* L. | -2.395601 | 57.669756 |
| *Hordeum murinum* L. | -18.1112531 | 27.7585095 |
| *Hordeum murinum* L. | -25.1211 | 36.97051 |
| *Hordeum murinum* L. | -27.19854 | 38.76079 |
| *Hordeum murinum* L. | -28.04289 | 39.04466 |
| *Hordeum murinum* L. | -31.15076 | 39.43924 |
| *Hordeum murinum* L. | -38.055 | -57.543 |
| *Hordeum murinum* L. | -56 | -33 |
| *Hordeum murinum* L. | -57 | -37.25 |
| *Hordeum murinum* L. | -57.421162 | -37.745583 |
| *Hordeum murinum* L. | -4.1310855 | 31.9086771 |
| *Hordeum murinum* L. | -4.516667 | 34.916667 |
| *Hordeum murinum* L. | -4.429 | 36.763 |
| *Hordeum murinum* L. | -3.606491 | 37.814149 |
| *Hordeum murinum* L. | -3.652 | 40.185 |
| *Hordeum murinum* L. | -4.919 | 42.972 |
| *Hordeum murinum* L. | -2.9039 | 47.38555 |
| *Hordeum murinum* L. | -3.49415 | 48.826348 |
| *Hordeum murinum* L. | -3.1791 | 51.481602 |
| *Hordeum murinum* L. | -2.951257 | 53.501903 |
| *Hordeum murinum* L. | -2.81201 | 55.618972 |
| *Hordeum murinum* L. | -3.258912 | 57.668602 |
| *Hordeum murinum* L. | -59.78 | -32.17 |
| *Hordeum murinum* L. | -58.480697 | -34.591617 |
| *Hordeum murinum* L. | -59.2 | -37.33 |
| *Hordeum murinum* L. | -57.77782 | -37.947426 |
| *Hordeum murinum* L. | -58.973148 | -51.827566 |
| *Hordeum murinum* L. | -60.870173 | -31.731105 |
| *Hordeum murinum* L. | -60.78 | -34.17 |
| *Hordeum murinum* L. | -61.040459 | -36.273121 |
| *Hordeum murinum* L. | -62.18 | -38.57 |
| *Hordeum murinum* L. | -64.23 | -27.43 |
| *Hordeum murinum* L. | -64 | -35 |
| *Hordeum murinum* L. | -64.01667 | -36.51667 |
| *Hordeum murinum* L. | -64 | -38 |
| *Hordeum murinum* L. | -64.36 | -40.07 |
| *Hordeum murinum* L. | -67.319167 | -33.671389 |
| *Hordeum murinum* L. | -65.044722 | -37.447222 |
| *Hordeum murinum* L. | -66.06 | -39.48 |
| *Hordeum murinum* L. | -67.5 | -40.88 |
| *Hordeum murinum* L. | -66.06 | -43.26 |
| *Hordeum murinum* L. | -65.996706 | -45.103326 |
| *Hordeum murinum* L. | -67.471944 | -47.786944 |
| *Hordeum murinum* L. | -6.39694 | 32.19694 |
| *Hordeum murinum* L. | -7.29 | 33.66 |
| *Hordeum murinum* L. | -6.15 | 35.19 |
| *Hordeum murinum* L. | -6.23 | 37.57 |
| *Hordeum murinum* L. | -5.62 | 42.47 |
| *Hordeum murinum* L. | -6.24 | 42.91 |
| *Hordeum murinum* L. | -5.12334 | 48.45716 |
| *Hordeum murinum* L. | -5.539715 | 50.107069 |
| *Hordeum murinum* L. | -6.152778 | 53.269167 |
| *Hordeum murinum* L. | -6.55438 | 56.657313 |
| *Hordeum murinum* L. | -68.165902 | -16.492803 |
| *Hordeum murinum* L. | -69.094444 | -34.047222 |
| *Hordeum murinum* L. | -69.792951 | -35.215112 |
| *Hordeum murinum* L. | -68.88 | -38.87 |
| *Hordeum murinum* L. | -68.9061 | -41.81 |
| *Hordeum murinum* L. | -69.99 | -42.66 |
| *Hordeum murinum* L. | -67.51528 | -47.39556 |
| *Hordeum murinum* L. | -68.393889 | -48.855833 |
| *Hordeum murinum* L. | -68.512833 | -50.022564 |
| *Hordeum murinum* L. | -70.055 | -14.834444 |
| *Hordeum murinum* L. | -70.55 | -24.55 |
| *Hordeum murinum* L. | -71 | -30 |
| *Hordeum murinum* L. | -71.55 | -31.55 |
| *Hordeum murinum* L. | -71.557044 | -33.018313 |
| *Hordeum murinum* L. | -71.03 | -36.83 |
| *Hordeum murinum* L. | -71.97262 | -39.277687 |
| *Hordeum murinum* L. | -71.55 | -41.43 |
| *Hordeum murinum* L. | -71.49 | -43.02 |
| *Hordeum murinum* L. | -70.75 | -46.63 |
| *Hordeum murinum* L. | -72.276597 | -50.341595 |
| *Hordeum murinum* L. | -70.921373 | -53.164786 |
| *Hordeum murinum* L. | -72.1 | 42.43333 |
| *Hordeum murinum* L. | -73.114175 | -36.796772 |
| *Hordeum murinum* L. | -72.809575 | -39.859936 |
| *Hordeum murinum* L. | -73.129188 | -40.572537 |
| *Hordeum murinum* L. | -73.086869 | -47.554498 |
| *Hordeum murinum* L. | -73.0518 | 41.489 |
| *Hordeum murinum* L. | -77.036667 | 38.895 |
| *Hordeum murinum* L. | -78.143994 | 35.694629 |
| *Hordeum murinum* L. | -81.103852 | 33.736123 |
| *Hordeum murinum* L. | -83.377078 | 33.950126 |
| *Hordeum murinum* L. | -85.505013 | 36.164246 |
| *Hordeum murinum* L. | -87.96 | 30.68 |
| *Hordeum murinum* L. | -94.706869 | 33.252216 |
| *Hordeum murinum* L. | -97.473241 | 30.629499 |
| *Hordeum murinum* L. | -96.758655 | 32.774525 |
| *Hordeum murinum* L. | 1.934465 | 34.297022 |
| *Hordeum murinum* L. | 0.776453 | 35.700472 |
| *Hordeum murinum* L. | 1.3 | 39 |
| *Hordeum murinum* L. | 2.00554 | 42.47053 |
| *Hordeum murinum* L. | 2.3867 | 44.69539 |
| *Hordeum murinum* L. | 2.48494 | 45.00195 |
| *Hordeum murinum* L. | 1.32557 | 47.74749 |
| *Hordeum murinum* L. | 0.1218 | 52.205299 |
| *Hordeum murinum* L. | 1.489543 | 52.861146 |
| *Hordeum murinum* L. | 100.62 | 36.28 |
| *Hordeum murinum* L. | 10.33 | 32.4 |
| *Hordeum murinum* L. | 10.66 | 34.66 |
| *Hordeum murinum* L. | 10.05 | 36.28 |
| *Hordeum murinum* L. | 12.335935 | 37.998391 |
| *Hordeum murinum* L. | 11.819167 | 42.056667 |
| *Hordeum murinum* L. | 10.617747 | 43.841513 |
| *Hordeum murinum* L. | 11.460916 | 45.474528 |
| *Hordeum murinum* L. | 11.458212 | 49.474925 |
| *Hordeum murinum* L. | 12.439255 | 52.406044 |
| *Hordeum murinum* L. | 11.2487 | 53.9484 |
| *Hordeum murinum* L. | 10.6 | 55.1 |
| *Hordeum murinum* L. | 11.93079 | 57.69181 |
| *Hordeum murinum* L. | 10.671 | 63.434 |
| *Hordeum murinum* L. | 113.666667 | -26.5 |
| *Hordeum murinum* L. | 114.166667 | -27.666667 |
| *Hordeum murinum* L. | 114.983333 | -30.333333 |
| *Hordeum murinum* L. | 114.9939 | -33.772717 |
| *Hordeum murinum* L. | 115.324454 | -26.098727 |
| *Hordeum murinum* L. | 115 | -28.333333 |
| *Hordeum murinum* L. | 116.833333 | -31.333333 |
| *Hordeum murinum* L. | 115 | -33.833333 |
| *Hordeum murinum* L. | 118.033333 | -26.366667 |
| *Hordeum murinum* L. | 119.5 | -30.333333 |
| *Hordeum murinum* L. | 119 | -34 |
| *Hordeum murinum* L. | 117.883333 | -35.016667 |
| *Hordeum murinum* L. | 12.863711 | 35.864172 |
| *Hordeum murinum* L. | 14.05 | 37.85 |
| *Hordeum murinum* L. | 13.620278 | 42.339167 |
| *Hordeum murinum* L. | 12.83333 | 42.83333 |
| *Hordeum murinum* L. | 14.57111 | 45.02472 |
| *Hordeum murinum* L. | 13.047222 | 47.809167 |
| *Hordeum murinum* L. | 12.545977 | 52.415787 |
| *Hordeum murinum* L. | 13.401306 | 52.565063 |
| *Hordeum murinum* L. | 14.7825 | 55.2878 |
| *Hordeum murinum* L. | 14.89544 | 58.45494 |
| *Hordeum murinum* L. | 14.54156 | 61.00651 |
| *Hordeum murinum* L. | 120.516667 | -29.35 |
| *Hordeum murinum* L. | 121.333333 | -30.833333 |
| *Hordeum murinum* L. | 121.666667 | -32.833333 |
| *Hordeum murinum* L. | 122.816667 | -22.883333 |
| *Hordeum murinum* L. | 122.833333 | -32.166667 |
| *Hordeum murinum* L. | 123.039412 | -33.81439 |
| *Hordeum murinum* L. | 124.743798 | 37.959691 |
| *Hordeum murinum* L. | 125.666667 | -30.166667 |
| *Hordeum murinum* L. | 126.259492 | 33.302146 |
| *Hordeum murinum* L. | 126.861557 | 37.354223 |
| *Hordeum murinum* L. | 126.889611 | 37.56575 |
| *Hordeum murinum* L. | 129.99795 | -28.43542 |
| *Hordeum murinum* L. | 128.166667 | -30.833333 |
| *Hordeum murinum* L. | 127.7805 | 26.158 |
| *Hordeum murinum* L. | 127.63588 | 34.90985 |
| *Hordeum murinum* L. | 129.17467 | 35.18114 |
| *Hordeum murinum* L. | 129.124708 | 37.515094 |
| *Hordeum murinum* L. | 132.333333 | -31.833333 |
| *Hordeum murinum* L. | 134 | -23.666667 |
| *Hordeum murinum* L. | 133.301069 | -26.99317 |
| *Hordeum murinum* L. | 134.09081 | -29.949011 |
| *Hordeum murinum* L. | 133.5 | -32 |
| *Hordeum murinum* L. | 134.5 | -32.666667 |
| *Hordeum murinum* L. | 133.541144 | 33.490872 |
| *Hordeum murinum* L. | 134.78333 | 35.63333 |
| *Hordeum murinum* L. | 135.734407 | -29.965389 |
| *Hordeum murinum* L. | 135 | -32.5 |
| *Hordeum murinum* L. | 136.166667 | -34.166667 |
| *Hordeum murinum* L. | 136.833333 | -35.333333 |
| *Hordeum murinum* L. | 135.84291 | 34.58167 |
| *Hordeum murinum* L. | 137.283333 | 36.733333 |
| *Hordeum murinum* L. | 138 | -29.666667 |
| *Hordeum murinum* L. | 137.833333 | -32.5 |
| *Hordeum murinum* L. | 139.166667 | -33.333333 |
| *Hordeum murinum* L. | 138.833333 | -35.333333 |
| *Hordeum murinum* L. | 139.934269 | 34.920011 |
| *Hordeum murinum* L. | 138 | 36 |
| *Hordeum murinum* L. | 139.033333 | 37.916667 |
| *Hordeum murinum* L. | 141.833333 | -20.5 |
| *Hordeum murinum* L. | 140 | -22.666667 |
| *Hordeum murinum* L. | 142 | -29.5 |
| *Hordeum murinum* L. | 141.612032 | -31.371622 |
| *Hordeum murinum* L. | 142.427079 | -34.433269 |
| *Hordeum murinum* L. | 140.333333 | -36.166667 |
| *Hordeum murinum* L. | 142.166667 | -37.833333 |
| *Hordeum murinum* L. | 140.75288 | 35.83085 |
| *Hordeum murinum* L. | 140.423869 | 38.601781 |
| *Hordeum murinum* L. | 141.48883 | 40.55286 |
| *Hordeum murinum* L. | 144.197856 | -23.397925 |
| *Hordeum murinum* L. | 144 | -26 |
| *Hordeum murinum* L. | 142.515833 | -29.333889 |
| *Hordeum murinum* L. | 143.987546 | -31.448753 |
| *Hordeum murinum* L. | 143.47017 | -34.815313 |
| *Hordeum murinum* L. | 144.479734 | -35.102916 |
| *Hordeum murinum* L. | 143.5 | -38.166667 |
| *Hordeum murinum* L. | 144.666667 | -40.5 |
| *Hordeum murinum* L. | 146.560349 | -29.432497 |
| *Hordeum murinum* L. | 147.485054 | -30.907631 |
| *Hordeum murinum* L. | 145.876153 | -33.085393 |
| *Hordeum murinum* L. | 146.678531 | -35.236348 |
| *Hordeum murinum* L. | 145 | -37.666667 |
| *Hordeum murinum* L. | 146.333333 | -41.333333 |
| *Hordeum murinum* L. | 147.333333 | -42.833333 |
| *Hordeum murinum* L. | 149.177592 | -24.332287 |
| *Hordeum murinum* L. | 149 | -27 |
| *Hordeum murinum* L. | 147.675104 | -29.998551 |
| *Hordeum murinum* L. | 148.328666 | -32.111072 |
| *Hordeum murinum* L. | 149.695159 | -34.720942 |
| *Hordeum murinum* L. | 148.278939 | -35.29507 |
| *Hordeum murinum* L. | 148 | -39.666667 |
| *Hordeum murinum* L. | 147.5 | -41.166667 |
| *Hordeum murinum* L. | 147.5 | -43 |
| *Hordeum murinum* L. | 152 | -24.5 |
| *Hordeum murinum* L. | 151.5 | -27.5 |
| *Hordeum murinum* L. | 151.372392 | -29.812536 |
| *Hordeum murinum* L. | 150.174423 | -30.531879 |
| *Hordeum murinum* L. | 150.31854 | -33.710173 |
| *Hordeum murinum* L. | 150.055703 | -35.911033 |
| *Hordeum murinum* L. | 153 | -27.5 |
| *Hordeum murinum* L. | 152.833333 | -28 |
| *Hordeum murinum* L. | 152.79435 | -31.35364 |
| *Hordeum murinum* L. | 159 | -31.5 |
| *Hordeum murinum* L. | 17.125 | -29.625 |
| *Hordeum murinum* L. | 17.416944 | -30.170833 |
| *Hordeum murinum* L. | 15.16667 | 32.33333 |
| *Hordeum murinum* L. | 15 | 37.13 |
| *Hordeum murinum* L. | 15.655033 | 38.087125 |
| *Hordeum murinum* L. | 15.10985 | 40.325813 |
| *Hordeum murinum* L. | 16.430658 | 43.508788 |
| *Hordeum murinum* L. | 15.934232 | 45.805851 |
| *Hordeum murinum* L. | 16.30881 | 48.17825 |
| *Hordeum murinum* L. | 16.885669 | 52.45078 |
| *Hordeum murinum* L. | 15.0248 | 53.3418 |
| *Hordeum murinum* L. | 16.446183 | 56.558041 |
| *Hordeum murinum* L. | 16.10622 | 58.59592 |
| *Hordeum murinum* L. | 17.316587 | 61.867609 |
| *Hordeum murinum* L. | 17.32118 | 62.51171 |
| *Hordeum murinum* L. | 167.9935 | -29.0406 |
| *Hordeum murinum* L. | 168.942935 | -44.96212 |
| *Hordeum murinum* L. | 169.309 | -45.217 |
| *Hordeum murinum* L. | 17.625 | -29.625 |
| *Hordeum murinum* L. | 19.875 | -31.375 |
| *Hordeum murinum* L. | 18.375 | -33.875 |
| *Hordeum murinum* L. | 19.8 | 39.6 |
| *Hordeum murinum* L. | 18.89306 | 42.39694 |
| *Hordeum murinum* L. | 18.1 | 42.63333 |
| *Hordeum murinum* L. | 19.8 | 45 |
| *Hordeum murinum* L. | 19.9667 | 47.6833 |
| *Hordeum murinum* L. | 18.684799 | 50.294371 |
| *Hordeum murinum* L. | 17.992491 | 53.130507 |
| *Hordeum murinum* L. | 18.86665 | 57.4347 |
| *Hordeum murinum* L. | 18.306345 | 57.608677 |
| *Hordeum murinum* L. | 19.943331 | 60.087819 |
| *Hordeum murinum* L. | 17.8701 | 62.6751 |
| *Hordeum murinum* L. | 170.095 | -43.719 |
| *Hordeum murinum* L. | 170.638027 | -45.848518 |
| *Hordeum murinum* L. | 173.370611 | -34.919444 |
| *Hordeum murinum* L. | 174.75 | -36.73333 |
| *Hordeum murinum* L. | 174.832802 | -39.865274 |
| *Hordeum murinum* L. | 174.748 | -41.278 |
| *Hordeum murinum* L. | 172.731634 | -43.516466 |
| *Hordeum murinum* L. | 175.715051 | -36.828309 |
| *Hordeum murinum* L. | 176.325785 | -38.046612 |
| *Hordeum murinum* L. | 175.616579 | -40.35566 |
| *Hordeum murinum* L. | 177.916667 | -38.583333 |
| *Hordeum murinum* L. | 2.923126 | 33.754772 |
| *Hordeum murinum* L. | 4.165784 | 36.337806 |
| *Hordeum murinum* L. | 2.704859 | 39.640602 |
| *Hordeum murinum* L. | 3.07931 | 42.49319 |
| *Hordeum murinum* L. | 3.00513 | 44.69704 |
| *Hordeum murinum* L. | 3.0687 | 45.0841 |
| *Hordeum murinum* L. | 4.35256 | 48.96621 |
| *Hordeum murinum* L. | 4.15271 | 52.003681 |
| *Hordeum murinum* L. | 4.80714 | 53.046163 |
| *Hordeum murinum* L. | 20.118889 | -32.125833 |
| *Hordeum murinum* L. | 20.375 | -34.125 |
| *Hordeum murinum* L. | 21.87586 | 32 |
| *Hordeum murinum* L. | 21.668016 | 32.757393 |
| *Hordeum murinum* L. | 22.37583 | 37.09083 |
| *Hordeum murinum* L. | 20.68556 | 38.34917 |
| *Hordeum murinum* L. | 22.16833 | 40.66333 |
| *Hordeum murinum* L. | 20.39245 | 44.820942 |
| *Hordeum murinum* L. | 20.44 | 46.7761 |
| *Hordeum murinum* L. | 21.729478 | 48.627042 |
| *Hordeum murinum* L. | 21.037285 | 52.161331 |
| *Hordeum murinum* L. | 20.4979 | 54.7105 |
| *Hordeum murinum* L. | 20.889409 | 55.223004 |
| *Hordeum murinum* L. | 22.260258 | 60.535398 |
| *Hordeum murinum* L. | 21.556791 | 63.043235 |
| *Hordeum murinum* L. | 24.08944 | -32.20194 |
| *Hordeum murinum* L. | 22.96483 | -34.083972 |
| *Hordeum murinum* L. | 24.80167 | 34.93139 |
| *Hordeum murinum* L. | 24.5 | 37.1 |
| *Hordeum murinum* L. | 22.7 | 38.9 |
| *Hordeum murinum* L. | 23.5203 | 42.3486 |
| *Hordeum murinum* L. | 23.35138 | 42.670922 |
| *Hordeum murinum* L. | 23.67944 | 46.79083 |
| *Hordeum murinum* L. | 24.079997 | 49.817335 |
| *Hordeum murinum* L. | 23.24219 | 50.72058 |
| *Hordeum murinum* L. | 23.8783 | 52.7429 |
| *Hordeum murinum* L. | 24.75 | 59.45 |
| *Hordeum murinum* L. | 24.925127 | 60.170408 |
| *Hordeum murinum* L. | 22.964918 | 63.541598 |
| *Hordeum murinum* L. | 25.092539 | -28.99853 |
| *Hordeum murinum* L. | 26.20806 | -32.33306 |
| *Hordeum murinum* L. | 25.5 | -33.7 |
| *Hordeum murinum* L. | 26.15 | 34.95 |
| *Hordeum murinum* L. | 25.2 | 35.2 |
| *Hordeum murinum* L. | 27.22111 | 38.46972 |
| *Hordeum murinum* L. | 25.6 | 42.1 |
| *Hordeum murinum* L. | 26.083334 | 44.5 |
| *Hordeum murinum* L. | 27.173882 | 45.69763 |
| *Hordeum murinum* L. | 25.9342 | 48.2642 |
| *Hordeum murinum* L. | 26.254124 | 50.522303 |
| *Hordeum murinum* L. | 26.033998 | 53.125073 |
| *Hordeum murinum* L. | 25.916667 | 59.25 |
| *Hordeum murinum* L. | 25.018642 | 60.110161 |
| *Hordeum murinum* L. | 25.405045 | 65.023135 |
| *Hordeum murinum* L. | 29.625 | -29.875 |
| *Hordeum murinum* L. | 27.959 | -30.6757 |
| *Hordeum murinum* L. | 29.55082 | 30.89981 |
| *Hordeum murinum* L. | 28.75 | 36.66667 |
| *Hordeum murinum* L. | 27.811025 | 37.856976 |
| *Hordeum murinum* L. | 28.78412 | 41.10488 |
| *Hordeum murinum* L. | 28.22859 | 44.24792 |
| *Hordeum murinum* L. | 27.556836 | 47.188297 |
| *Hordeum murinum* L. | 28.1884 | 48.2308 |
| *Hordeum murinum* L. | 28.673369 | 50.259391 |
| *Hordeum murinum* L. | 27.764685 | 53.825849 |
| *Hordeum murinum* L. | 29.033508 | 61.975724 |
| *Hordeum murinum* L. | 30 | 27 |
| *Hordeum murinum* L. | 30.2 | 30.5 |
| *Hordeum murinum* L. | 32.422222 | 34.747222 |
| *Hordeum murinum* L. | 32.431942 | 36.160356 |
| *Hordeum murinum* L. | 31.144347 | 39.020094 |
| *Hordeum murinum* L. | 32.258105 | 40.004666 |
| *Hordeum murinum* L. | 31.825991 | 47.116609 |
| *Hordeum murinum* L. | 31.446677 | 47.703331 |
| *Hordeum murinum* L. | 30.503652 | 50.430322 |
| *Hordeum murinum* L. | 31.922481 | 52.532667 |
| *Hordeum murinum* L. | 30.2134 | 60.3173 |
| *Hordeum murinum* L. | 34.92 | 29.52 |
| *Hordeum murinum* L. | 34.717194 | 31.506139 |
| *Hordeum murinum* L. | 34.934152 | 32.705359 |
| *Hordeum murinum* L. | 33.131671 | 36.138209 |
| *Hordeum murinum* L. | 32.85 | 39.61667 |
| *Hordeum murinum* L. | 32.9 | 40 |
| *Hordeum murinum* L. | 33.4868 | 44.5912 |
| *Hordeum murinum* L. | 33.722963 | 46.436214 |
| *Hordeum murinum* L. | 33.8314 | 48.8703 |
| *Hordeum murinum* L. | 33.24773 | 52.031251 |
| *Hordeum murinum* L. | 34.349049 | 53.264633 |
| *Hordeum murinum* L. | 34.2728 | 62.2048 |
| *Hordeum murinum* L. | 37.488472 | 24.598556 |
| *Hordeum murinum* L. | 37.239667 | 25.196583 |
| *Hordeum murinum* L. | 35.02 | 29.89 |
| *Hordeum murinum* L. | 35.392492 | 31.341215 |
| *Hordeum murinum* L. | 35.19317 | 32.751014 |
| *Hordeum murinum* L. | 36.619167 | 37.063333 |
| *Hordeum murinum* L. | 35.179672 | 39.060596 |
| *Hordeum murinum* L. | 35.66222 | 40.97056 |
| *Hordeum murinum* L. | 37.4554 | 44.7092 |
| *Hordeum murinum* L. | 36.825681 | 46.738429 |
| *Hordeum murinum* L. | 35.0423 | 48.4684 |
| *Hordeum murinum* L. | 36.225381 | 50.004752 |
| *Hordeum murinum* L. | 37.029876 | 53.564939 |
| *Hordeum murinum* L. | 35.9119 | 56.8596 |
| *Hordeum murinum* L. | 39.127444 | 21.909278 |
| *Hordeum murinum* L. | 39.910583 | 22.933972 |
| *Hordeum murinum* L. | 39.932462 | 25.662085 |
| *Hordeum murinum* L. | 38 | 30.75 |
| *Hordeum murinum* L. | 38.69917 | 33.50222 |
| *Hordeum murinum* L. | 38.505131 | 35.013134 |
| *Hordeum murinum* L. | 38.1 | 38.48333 |
| *Hordeum murinum* L. | 38.0765 | 44.5615 |
| *Hordeum murinum* L. | 38.271838 | 46.721469 |
| *Hordeum murinum* L. | 37.563 | 48.833 |
| *Hordeum murinum* L. | 39.205318 | 51.657283 |
| *Hordeum murinum* L. | 37.613417 | 54.29104 |
| *Hordeum murinum* L. | 37.72917 | 55.723881 |
| *Hordeum murinum* L. | 42.046694 | 16.779389 |
| *Hordeum murinum* L. | 41.315033 | 19.845033 |
| *Hordeum murinum* L. | 40.333333 | 21.166667 |
| *Hordeum murinum* L. | 40.256389 | 37.943333 |
| *Hordeum murinum* L. | 42.367 | 41.517 |
| *Hordeum murinum* L. | 41.107 | 44.9926 |
| *Hordeum murinum* L. | 41.36221 | 46.8423 |
| *Hordeum murinum* L. | 40.193 | 47.7794 |
| *Hordeum murinum* L. | 40.13289 | 50.450899 |
| *Hordeum murinum* L. | 40.496928 | 52.898237 |
| *Hordeum murinum* L. | 42.592278 | 17.168222 |
| *Hordeum murinum* L. | 42.508883 | 18.195233 |
| *Hordeum murinum* L. | 43.866367 | 24.807683 |
| *Hordeum murinum* L. | 44.983333 | 26.333333 |
| *Hordeum murinum* L. | 43.889624 | 34.096602 |
| *Hordeum murinum* L. | 42.961318 | 36.834468 |
| *Hordeum murinum* L. | 44.96667 | 38.55 |
| *Hordeum murinum* L. | 44.8 | 41.8 |
| *Hordeum murinum* L. | 42.907246 | 43.371921 |
| *Hordeum murinum* L. | 44.25111 | 46.309305 |
| *Hordeum murinum* L. | 44.513173 | 48.71355 |
| *Hordeum murinum* L. | 47.206667 | 17.236528 |
| *Hordeum murinum* L. | 46.245306 | 24.448472 |
| *Hordeum murinum* L. | 46.576167 | 25.345972 |
| *Hordeum murinum* L. | 46.033333 | 27.683333 |
| *Hordeum murinum* L. | 45.583333 | 34.5 |
| *Hordeum murinum* L. | 46.95 | 35.05 |
| *Hordeum murinum* L. | 47.36667 | 38.4 |
| *Hordeum murinum* L. | 45.03 | 40.36 |
| *Hordeum murinum* L. | 45.5706 | 42.6842 |
| *Hordeum murinum* L. | 46.4393 | 45.0055 |
| *Hordeum murinum* L. | 45.4022 | 50.0853 |
| *Hordeum murinum* L. | 49.577722 | 25.554444 |
| *Hordeum murinum* L. | 47.783333 | 29.585278 |
| *Hordeum murinum* L. | 49.06667 | 31.86667 |
| *Hordeum murinum* L. | 47.7 | 33.16667 |
| *Hordeum murinum* L. | 48.87389 | 38.4325 |
| *Hordeum murinum* L. | 49.3833 | 40 |
| *Hordeum murinum* L. | 47.633907 | 42.900198 |
| *Hordeum murinum* L. | 48.2338 | 46.1781 |
| *Hordeum murinum* L. | 50.094278 | 25.597861 |
| *Hordeum murinum* L. | 51.238061 | 29.221522 |
| *Hordeum murinum* L. | 51.15 | 30.35 |
| *Hordeum murinum* L. | 51.26667 | 36.36667 |
| *Hordeum murinum* L. | 50.349283 | 40.29535 |
| *Hordeum murinum* L. | 51.144169 | 43.64856 |
| *Hordeum murinum* L. | 52.0418 | 55.7619 |
| *Hordeum murinum* L. | 53 | 32 |
| *Hordeum murinum* L. | 54.5 | 37 |
| *Hordeum murinum* L. | 54.145946 | 39.334894 |
| *Hordeum murinum* L. | 52.966667 | 40.033333 |
| *Hordeum murinum* L. | 53.46728 | 43.36668 |
| *Hordeum murinum* L. | 53.2069 | 56.8527 |
| *Hordeum murinum* L. | 52.6414 | 58.137 |
| *Hordeum murinum* L. | 55.496492 | -21.109744 |
| *Hordeum murinum* L. | 57.4 | 29.966667 |
| *Hordeum murinum* L. | 57.473159 | 30.204707 |
| *Hordeum murinum* L. | 56.266667 | 38.433333 |
| *Hordeum murinum* L. | 57.2194 | 65.1362 |
| *Hordeum murinum* L. | 59.6 | 36.3 |
| *Hordeum murinum* L. | 58.38 | 37.95 |
| *Hordeum murinum* L. | 6.61641 | 36.336926 |
| *Hordeum murinum* L. | 7.407445 | 43.724644 |
| *Hordeum murinum* L. | 5.31292 | 46.49462 |
| *Hordeum murinum* L. | 5.82059 | 48.4797 |
| *Hordeum murinum* L. | 5.64187 | 51.970645 |
| *Hordeum murinum* L. | 6.0444 | 52.625201 |
| *Hordeum murinum* L. | 7.492386 | 58.059571 |
| *Hordeum murinum* L. | 62.11667 | 34.63333 |
| *Hordeum murinum* L. | 60.369951 | 41.380081 |
| *Hordeum murinum* L. | 62.86667 | 34.23333 |
| *Hordeum murinum* L. | 64.78333 | 35.91667 |
| *Hordeum murinum* L. | 64.416661 | 39.771758 |
| *Hordeum murinum* L. | 67.1297 | 29.903343 |
| *Hordeum murinum* L. | 66.58333 | 31.9 |
| *Hordeum murinum* L. | 65.12361 | 36.9525 |
| *Hordeum murinum* L. | 66.625 | 38.3111 |
| *Hordeum murinum* L. | 66.775948 | 40.527965 |
| *Hordeum murinum* L. | 65.3411 | 55.441 |
| *Hordeum murinum* L. | 67.878487 | 29.550776 |
| *Hordeum murinum* L. | 69.448056 | 31.341111 |
| *Hordeum murinum* L. | 69.53 | 34.6 |
| *Hordeum murinum* L. | 68.5 | 36.75 |
| *Hordeum murinum* L. | 68 | 39.2 |
| *Hordeum murinum* L. | 69.3 | 41.33333 |
| *Hordeum murinum* L. | 69.67485 | 42.78523 |
| *Hordeum murinum* L. | 8.71 | 34.4 |
| *Hordeum murinum* L. | 8.88 | 35.2 |
| *Hordeum murinum* L. | 9.333611 | 39.883889 |
| *Hordeum murinum* L. | 9.628151 | 40.994742 |
| *Hordeum murinum* L. | 7.639355 | 43.879524 |
| *Hordeum murinum* L. | 8.663235 | 46.875631 |
| *Hordeum murinum* L. | 8.520713 | 49.836182 |
| *Hordeum murinum* L. | 9.703305 | 52.326999 |
| *Hordeum murinum* L. | 9.883196 | 54.947839 |
| *Hordeum murinum* L. | 8.436429 | 55.467344 |
| *Hordeum murinum* L. | 9.1278309 | 58.6338932 |
| *Hordeum murinum* L. | 72.08333 | 34.63333 |
| *Hordeum murinum* L. | 71.8 | 35.8 |
| *Hordeum murinum* L. | 71.3 | 38.9 |
| *Hordeum murinum* L. | 71.937602 | 40.515425 |
| *Hordeum murinum* L. | 70.384255 | 43.138801 |
| *Hordeum murinum* L. | 73 | 34.5 |
| *Hordeum murinum* L. | 72.58 | 35.33 |
| *Hordeum murinum* L. | 73.041925 | 40.526333 |
| *Hordeum murinum* L. | 74.271945 | 42.869979 |
| *Hordeum murinum* L. | 77 | 33 |
| *Hordeum murinum* L. | 75.74056 | 41.83833 |
| *Hordeum murinum* L. | 77.257609 | 43.335409 |
| *Hordeum murinum* L. | 79.616202 | 22.883478 |
| *Hordeum murinum* L. | 78.405064 | 42.497274 |
| *Hordeum murinum* L. | 78.758148 | 44.864075 |
| *Hordeum murinum* L. | 82 | 34 |
| *Hordeum murinum* L. | 94.9 | 36.42 |
| *Hordeum murinum* L. | 98.08 | 36.3 |
| *Hordeum muticum* J.Presl | -0.892222 | 51.095278 |
| *Hordeum muticum* J.Presl | -56.945278 | -36.646944 |
| *Hordeum muticum* J.Presl | -58.62 | -34.65 |
| *Hordeum muticum* J.Presl | -65 | -17 |
| *Hordeum muticum* J.Presl | -65.617778 | -17.6375 |
| *Hordeum muticum* J.Presl | -65.588778 | -19.5285 |
| *Hordeum muticum* J.Presl | -65.101111 | -20.264167 |
| *Hordeum muticum* J.Presl | -65.10297 | -21.728871 |
| *Hordeum muticum* J.Presl | -65.98 | -22.43 |
| *Hordeum muticum* J.Presl | -65.8 | -23.87 |
| *Hordeum muticum* J.Presl | -65.408333 | -24.208333 |
| *Hordeum muticum* J.Presl | -65.87 | -26.61 |
| *Hordeum muticum* J.Presl | -65.913889 | -28.016667 |
| *Hordeum muticum* J.Presl | -65.115278 | -38.246944 |
| *Hordeum muticum* J.Presl | -66.108333 | -17.408333 |
| *Hordeum muticum* J.Presl | -66.963518 | -18.171607 |
| *Hordeum muticum* J.Presl | -66.910527 | -20.350722 |
| *Hordeum muticum* J.Presl | -66.07 | -21.95 |
| *Hordeum muticum* J.Presl | -66.02 | -22.98 |
| *Hordeum muticum* J.Presl | -67.9521 | -16.5619 |
| *Hordeum muticum* J.Presl | -67.306147 | -17.299972 |
| *Hordeum muticum* J.Presl | -67.308333 | -18.008333 |
| *Hordeum muticum* J.Presl | -67.37 | -26.03 |
| *Hordeum muticum* J.Presl | -68.935278 | -15.885833 |
| *Hordeum muticum* J.Presl | -68.692209 | -16.440043 |
| *Hordeum muticum* J.Presl | -68.712385 | -17.201279 |
| *Hordeum muticum* J.Presl | -69.53 | -14.13 |
| *Hordeum muticum* J.Presl | -69.494836 | -18.188473 |
| *Hordeum muticum* J.Presl | -69.16888 | -19.68555 |
| *Hordeum muticum* J.Presl | -69.83 | -28.73 |
| *Hordeum muticum* J.Presl | -70.412231 | -13.988289 |
| *Hordeum muticum* J.Presl | -70.859722 | -14.598611 |
| *Hordeum muticum* J.Presl | -70.801389 | -15.683889 |
| *Hordeum muticum* J.Presl | -70.55 | -36.016667 |
| *Hordeum muticum* J.Presl | -71.93333 | -13.48333 |
| *Hordeum muticum* J.Presl | -71.26666 | -14.68333 |
| *Hordeum muticum* J.Presl | -71.527007 | -16.455353 |
| *Hordeum muticum* J.Presl | -72.05027 | -13.28666 |
| *Hordeum muticum* J.Presl | -73.490833 | -15.469444 |
| *Hordeum muticum* J.Presl | -74.375627 | -9.163761 |
| *Hordeum muticum* J.Presl | -74.159722 | -13.340833 |
| *Hordeum muticum* J.Presl | -74.074444 | -14.688611 |
| *Hordeum muticum* J.Presl | -75.93 | -11.93 |
| *Hordeum muticum* J.Presl | -75.1 | -12.33 |
| *Hordeum muticum* J.Presl | -76.4729 | -11.3859 |
| *Hordeum muticum* J.Presl | -77.38333 | -9.5 |
| *Hordeum muticum* J.Presl | -77.6 | -8.91666 |
| *Hordeum muticum* J.Presl | 0 | 0 |
| *Hordeum muticum* J.Presl | 116.02 | -30.5694 |
| *Hordeum muticum* J.Presl | 13.11376 | 55.77235 |
| *Hordeum muticum* J.Presl | 7.58379 | 47.193854 |
| *Hordeum pusillum* Nutt. | -97.82172 | 27.49069 |
| *Hordeum pusillum* Nutt. | -98.456172 | 29.682445 |
| *Hordeum pusillum* Nutt. | -97.82399 | 30.698507 |
| *Hordeum pusillum* Nutt. | -99.244074 | 33.157907 |
| *Hordeum pusillum* Nutt. | -97.551584 | 35.727463 |
| *Hordeum pusillum* Nutt. | -98.522697 | 38.985058 |
| *Hordeum pusillum* Nutt. | -97.649006 | 40.735081 |
| *Hordeum pusillum* Nutt. | -99.937914 | 42.768884 |
| *Hordeum pusillum* Nutt. | -100.277937 | 29.969052 |
| *Hordeum pusillum* Nutt. | -100.451341 | 31.851267 |
| *Hordeum pusillum* Nutt. | -101.30417 | 34.61925 |
| *Hordeum pusillum* Nutt. | -100.78068 | 37.11256 |
| *Hordeum pusillum* Nutt. | -102.2686 | 38.94366 |
| *Hordeum pusillum* Nutt. | -100.974864 | 42.183038 |
| *Hordeum pusillum* Nutt. | -101.848833 | 43.942067 |
| *Hordeum pusillum* Nutt. | -103.251756 | 29.821482 |
| *Hordeum pusillum* Nutt. | -104.816667 | 32.41 |
| *Hordeum pusillum* Nutt. | -102.63295 | 33.45874 |
| *Hordeum pusillum* Nutt. | -102.96684 | 37.45823 |
| *Hordeum pusillum* Nutt. | -104.772637 | 39.752542 |
| *Hordeum pusillum* Nutt. | -103.696183 | 41.851585 |
| *Hordeum pusillum* Nutt. | -103.473168 | 44.748586 |
| *Hordeum pusillum* Nutt. | -103.7357 | 45.5698 |
| *Hordeum pusillum* Nutt. | -106.391667 | 29.291667 |
| *Hordeum pusillum* Nutt. | -106.701073 | 31.961837 |
| *Hordeum pusillum* Nutt. | -106.604281 | 34.927779 |
| *Hordeum pusillum* Nutt. | -106.03492 | 36.32049 |
| *Hordeum pusillum* Nutt. | -105.21 | 39.77 |
| *Hordeum pusillum* Nutt. | -105.15 | 40.51 |
| *Hordeum pusillum* Nutt. | -107.41063 | 44.025724 |
| *Hordeum pusillum* Nutt. | -105.533 | 45.3445 |
| *Hordeum pusillum* Nutt. | -107.0189 | 47.6232 |
| *Hordeum pusillum* Nutt. | -107.683 | 27.7333 |
| *Hordeum pusillum* Nutt. | -108.954 | 32.234 |
| *Hordeum pusillum* Nutt. | -108.261337 | 32.795462 |
| *Hordeum pusillum* Nutt. | -108.496861 | 37.174136 |
| *Hordeum pusillum* Nutt. | -108.1284 | 39.2072 |
| *Hordeum pusillum* Nutt. | -109.22372 | 40.1108 |
| *Hordeum pusillum* Nutt. | -107.9497 | 43.9122 |
| *Hordeum pusillum* Nutt. | -110 | 47 |
| *Hordeum pusillum* Nutt. | -107.792617 | 47.775847 |
| *Hordeum pusillum* Nutt. | -110.165 | 23.4426 |
| *Hordeum pusillum* Nutt. | -110.671612 | 32.148664 |
| *Hordeum pusillum* Nutt. | -111.949753 | 34.357175 |
| *Hordeum pusillum* Nutt. | -111.838091 | 37.14184 |
| *Hordeum pusillum* Nutt. | -110.926667 | 37.798333 |
| *Hordeum pusillum* Nutt. | -111.560912 | 40.727041 |
| *Hordeum pusillum* Nutt. | -112.49151 | 45.695576 |
| *Hordeum pusillum* Nutt. | -111.153025 | 47.570796 |
| *Hordeum pusillum* Nutt. | -114.75 | 29.2 |
| *Hordeum pusillum* Nutt. | -112.57472 | 34.59194 |
| *Hordeum pusillum* Nutt. | -113.854167 | 37.206944 |
| *Hordeum pusillum* Nutt. | -114.251506 | 47.333263 |
| *Hordeum pusillum* Nutt. | -116.05 | 30.833333 |
| *Hordeum pusillum* Nutt. | -116.913962 | 32.623851 |
| *Hordeum pusillum* Nutt. | -116.459045 | 37.966377 |
| *Hordeum pusillum* Nutt. | -116.18367 | 43.619053 |
| *Hordeum pusillum* Nutt. | -117.03611 | 46.425 |
| *Hordeum pusillum* Nutt. | -118.550353 | 32.945258 |
| *Hordeum pusillum* Nutt. | -119.3333 | 35.5833 |
| *Hordeum pusillum* Nutt. | -120.1094 | 33.9053 |
| *Hordeum pusillum* Nutt. | -120.916667 | 37.3 |
| *Hordeum pusillum* Nutt. | -121.78585 | 38.53655 |
| *Hordeum pusillum* Nutt. | -120.308333 | 42.208333 |
| *Hordeum pusillum* Nutt. | -120.8179376 | 44.2695534 |
| *Hordeum pusillum* Nutt. | -123.233333 | 48.433333 |
| *Hordeum pusillum* Nutt. | -1.987659 | 52.108934 |
| *Hordeum pusillum* Nutt. | -1.718167 | 53.657667 |
| *Hordeum pusillum* Nutt. | -3.590118 | 57.575034 |
| *Hordeum pusillum* Nutt. | -58.118897 | -32.685812 |
| *Hordeum pusillum* Nutt. | -62.236111 | -32.969722 |
| *Hordeum pusillum* Nutt. | -62.65 | -36.06 |
| *Hordeum pusillum* Nutt. | -72.39194 | 41.65277 |
| *Hordeum pusillum* Nutt. | -74.935122 | 39.567831 |
| *Hordeum pusillum* Nutt. | -73.990892 | 40.584745 |
| *Hordeum pusillum* Nutt. | -76.207175 | 35.462075 |
| *Hordeum pusillum* Nutt. | -76.486715 | 38.968228 |
| *Hordeum pusillum* Nutt. | -78.901692 | 34.408223 |
| *Hordeum pusillum* Nutt. | -78.944659 | 36.004672 |
| *Hordeum pusillum* Nutt. | -78.180426 | 37.949234 |
| *Hordeum pusillum* Nutt. | -78.208333 | 40.408333 |
| *Hordeum pusillum* Nutt. | -81.845 | 29.738 |
| *Hordeum pusillum* Nutt. | -81.805517 | 31.020067 |
| *Hordeum pusillum* Nutt. | -81.13766 | 34.123311 |
| *Hordeum pusillum* Nutt. | -80.805167 | 35.208326 |
| *Hordeum pusillum* Nutt. | -81.682034 | 37.628519 |
| *Hordeum pusillum* Nutt. | -82.42488 | 40.58843 |
| *Hordeum pusillum* Nutt. | -84.799665 | 29.890147 |
| *Hordeum pusillum* Nutt. | -83.97915 | 30.1164 |
| *Hordeum pusillum* Nutt. | -84.406892 | 33.3462 |
| *Hordeum pusillum* Nutt. | -84.991364 | 37.017273 |
| *Hordeum pusillum* Nutt. | -84.790905 | 37.757787 |
| *Hordeum pusillum* Nutt. | -83.737562 | 42.292913 |
| *Hordeum pusillum* Nutt. | -84.485198 | 42.731239 |
| *Hordeum pusillum* Nutt. | -85.2 | 29.95 |
| *Hordeum pusillum* Nutt. | -85.955 | 31.797222 |
| *Hordeum pusillum* Nutt. | -86.144172 | 34.678425 |
| *Hordeum pusillum* Nutt. | -87.002128 | 35.976588 |
| *Hordeum pusillum* Nutt. | -87.270268 | 39.904373 |
| *Hordeum pusillum* Nutt. | -85.375685 | 40.451317 |
| *Hordeum pusillum* Nutt. | -89.970001 | 29.9 |
| *Hordeum pusillum* Nutt. | -89.396462 | 30.463058 |
| *Hordeum pusillum* Nutt. | -87.620232 | 34.528186 |
| *Hordeum pusillum* Nutt. | -88.99924 | 35.956943 |
| *Hordeum pusillum* Nutt. | -89.428931 | 37.626017 |
| *Hordeum pusillum* Nutt. | -88.188439 | 40.122786 |
| *Hordeum pusillum* Nutt. | -89.337998 | 43.025538 |
| *Hordeum pusillum* Nutt. | -90.088839 | 29.999151 |
| *Hordeum pusillum* Nutt. | -90.55072 | 30.903673 |
| *Hordeum pusillum* Nutt. | -92.052035 | 32.603942 |
| *Hordeum pusillum* Nutt. | -90.59129 | 35.767318 |
| *Hordeum pusillum* Nutt. | -90.464378 | 38.557105 |
| *Hordeum pusillum* Nutt. | -90.439383 | 41.518212 |
| *Hordeum pusillum* Nutt. | -94.067572 | 29.869967 |
| *Hordeum pusillum* Nutt. | -94.275519 | 31.059104 |
| *Hordeum pusillum* Nutt. | -93.009889 | 34.555825 |
| *Hordeum pusillum* Nutt. | -94.022897 | 35.348052 |
| *Hordeum pusillum* Nutt. | -93.492355 | 38.969936 |
| *Hordeum pusillum* Nutt. | -92.981453 | 42.041691 |
| *Hordeum pusillum* Nutt. | -94.202774 | 42.782223 |
| *Hordeum pusillum* Nutt. | -95.438308 | 28.948647 |
| *Hordeum pusillum* Nutt. | -96.167203 | 31.126269 |
| *Hordeum pusillum* Nutt. | -97.097494 | 32.589162 |
| *Hordeum pusillum* Nutt. | -97.471358 | 35.263797 |
| *Hordeum pusillum* Nutt. | -95.2379 | 39.00031 |
| *Hordeum pusillum* Nutt. | -96.136536 | 41.342992 |
| *Hordeum pusillum* Nutt. | -96.272917 | 43.735389 |
| *Hordeum pusillum* Nutt. | -96.368808 | 45.257671 |
| *Hordeum pusillum* Nutt. | 0.4585 | 51.2649 |
| *Hordeum pusillum* Nutt. | 100.62 | 36.28 |
| *Hordeum pusillum* Nutt. | 11.99812 | 57.67508 |
| *Hordeum pusillum* Nutt. | 13.69624 | 57.19077 |
| *Hordeum pusillum* Nutt. | 120.488297 | 26.360211 |
| *Hordeum pusillum* Nutt. | 126.559444 | 33.304444 |
| *Hordeum pusillum* Nutt. | 126.861557 | 37.354223 |
| *Hordeum pusillum* Nutt. | 127.793415 | 26.725705 |
| *Hordeum pusillum* Nutt. | 129.1553 | 37.4615 |
| *Hordeum pusillum* Nutt. | 139.678032 | 35.474096 |
| *Hordeum pusillum* Nutt. | 17.00874 | 58.76063 |
| *Hordeum pusillum* Nutt. | 4.79076 | 52.49969 |
| *Hordeum pusillum* Nutt. | 4.76118 | 52.50852 |
| *Hordeum pusillum* Nutt. | 23.830407 | 61.63199 |
| *Hordeum pusillum* Nutt. | 49.1223 | 55.7969 |
| *Hordeum pusillum* Nutt. | 53.2069 | 56.8527 |
| *Hordeum pusillum* Nutt. | 6.793927 | 51.392471 |
| *Hordeum pusillum* Nutt. | 7.577203 | 47.19836 |
| *Hordeum roshevitzii* Bowden | 100.13 | 37.33 |
| *Hordeum roshevitzii* Bowden | 100.25 | 38.18 |
| *Hordeum roshevitzii* Bowden | 101.07 | 41.97 |
| *Hordeum roshevitzii* Bowden | 102.872639 | 30.29234 |
| *Hordeum roshevitzii* Bowden | 104.23 | 33.27 |
| *Hordeum roshevitzii* Bowden | 103.68333 | 36.05 |
| *Hordeum roshevitzii* Bowden | 103.864 | 51.75 |
| *Hordeum roshevitzii* Bowden | 103.522356 | 52.83824 |
| *Hordeum roshevitzii* Bowden | 105.01 | 31.08 |
| *Hordeum roshevitzii* Bowden | 105.65 | 36.57 |
| *Hordeum roshevitzii* Bowden | 107.37291 | 47.811688 |
| *Hordeum roshevitzii* Bowden | 106.766667 | 50.366667 |
| *Hordeum roshevitzii* Bowden | 106.580042 | 52.829836 |
| *Hordeum roshevitzii* Bowden | 108.02 | 35.82 |
| *Hordeum roshevitzii* Bowden | 108.8 | 37.6 |
| *Hordeum roshevitzii* Bowden | 107.98 | 51.677 |
| *Hordeum roshevitzii* Bowden | 109.368 | 55.592 |
| *Hordeum roshevitzii* Bowden | 110.25 | 37.5 |
| *Hordeum roshevitzii* Bowden | 112.348311 | 45.69265 |
| *Hordeum roshevitzii* Bowden | 111.023 | 49.7216 |
| *Hordeum roshevitzii* Bowden | 110.607791 | 54.428101 |
| *Hordeum roshevitzii* Bowden | 111.1019 | 62.8987 |
| *Hordeum roshevitzii* Bowden | 113.833333 | 24.466667 |
| *Hordeum roshevitzii* Bowden | 114.7 | 41.15 |
| *Hordeum roshevitzii* Bowden | 114.972553 | 50.189096 |
| *Hordeum roshevitzii* Bowden | 116 | 42.25 |
| *Hordeum roshevitzii* Bowden | 117 | 43.67 |
| *Hordeum roshevitzii* Bowden | 116.97 | 45.5 |
| *Hordeum roshevitzii* Bowden | 115.8877 | 50.1362 |
| *Hordeum roshevitzii* Bowden | 118.283333 | 37.833333 |
| *Hordeum roshevitzii* Bowden | 117.6 | 44.58 |
| *Hordeum roshevitzii* Bowden | 119.77 | 49.22 |
| *Hordeum roshevitzii* Bowden | 118.414 | 50.294 |
| *Hordeum roshevitzii* Bowden | 120.92 | 44.55 |
| *Hordeum roshevitzii* Bowden | 122.6 | 46.06 |
| *Hordeum roshevitzii* Bowden | 125.316667 | 46.4 |
| *Hordeum roshevitzii* Bowden | 131.8446 | 42.9993 |
| *Hordeum roshevitzii* Bowden | 131.447 | 63.016 |
| *Hordeum roshevitzii* Bowden | 131.7176 | 65.8257 |
| *Hordeum roshevitzii* Bowden | 134.2514 | 62.1961 |
| *Hordeum roshevitzii* Bowden | 132.7613 | 65.4125 |
| *Hordeum roshevitzii* Bowden | 136.005 | 61.501 |
| *Hordeum roshevitzii* Bowden | 151.47606 | 60.48294 |
| *Hordeum roshevitzii* Bowden | 69.748 | 56.837 |
| *Hordeum roshevitzii* Bowden | 84.608333 | 51.208333 |
| *Hordeum roshevitzii* Bowden | 84.964 | 54.728 |
| *Hordeum roshevitzii* Bowden | 86 | 42 |
| *Hordeum roshevitzii* Bowden | 86.208333 | 42.708333 |
| *Hordeum roshevitzii* Bowden | 85.533 | 54.619 |
| *Hordeum roshevitzii* Bowden | 87.608333 | 43.808333 |
| *Hordeum roshevitzii* Bowden | 88.144 | 50.078 |
| *Hordeum roshevitzii* Bowden | 89.899 | 53.451 |
| *Hordeum roshevitzii* Bowden | 89.305 | 55.061 |
| *Hordeum roshevitzii* Bowden | 91.459 | 51.372 |
| *Hordeum roshevitzii* Bowden | 90.453333 | 53.0025 |
| *Hordeum roshevitzii* Bowden | 90.022 | 55.421 |
| *Hordeum roshevitzii* Bowden | 93.6907 | 51.8155 |
| *Hordeum roshevitzii* Bowden | 93.3423 | 52.8619 |
| *Hordeum roshevitzii* Bowden | 93.0076 | 56.2333 |
| *Hordeum roshevitzii* Bowden | 97.36 | 37.36 |
| *Hordeum roshevitzii* Bowden | 95.33 | 37.83 |
| *Hordeum roshevitzii* Bowden | 97.1833 | 48.0333 |
| *Hordeum roshevitzii* Bowden | 95.0678 | 50.0849 |
| *Hordeum roshevitzii* Bowden | 95.5017 | 56.4422 |
| *Hordeum roshevitzii* Bowden | 99.98 | 35.58 |
| *Hordeum roshevitzii* Bowden | 99.62 | 38.83 |
| *Hordeum spontaneum* K. Koch | -117.338889 | 33.966667 |
| *Hordeum spontaneum* K. Koch | -1.622154 | 53.717837 |
| *Hordeum spontaneum* K. Koch | -5.833906 | 43.153692 |
| *Hordeum spontaneum* K. Koch | 102 | 30 |
| *Hordeum spontaneum* K. Koch | 101.43 | 36.05 |
| *Hordeum spontaneum* K. Koch | 100 | 66 |
| *Hordeum spontaneum* K. Koch | 103.908333 | 30.008333 |
| *Hordeum spontaneum* K. Koch | 10.422028 | 49.496777 |
| *Hordeum spontaneum* K. Koch | 113.8 | 35.2 |
| *Hordeum spontaneum* K. Koch | 116.21 | 40.02 |
| *Hordeum spontaneum* K. Koch | 16.411992 | 40.822473 |
| *Hordeum spontaneum* K. Koch | 21.1667 | 32.3667 |
| *Hordeum spontaneum* K. Koch | 21.7167 | 32.7667 |
| *Hordeum spontaneum* K. Koch | 21.17639 | 39.74667 |
| *Hordeum spontaneum* K. Koch | 24.2333 | 31.8333 |
| *Hordeum spontaneum* K. Koch | 22.8333 | 32.6 |
| *Hordeum spontaneum* K. Koch | 24.2 | 35.366667 |
| *Hordeum spontaneum* K. Koch | 22.95 | 38.03333 |
| *Hordeum spontaneum* K. Koch | 27.1667 | 31.35 |
| *Hordeum spontaneum* K. Koch | 27.183333 | 35.466667 |
| *Hordeum spontaneum* K. Koch | 26.133333 | 38.316667 |
| *Hordeum spontaneum* K. Koch | 28.061283 | 36.379678 |
| *Hordeum spontaneum* K. Koch | 32.402778 | 34.755556 |
| *Hordeum spontaneum* K. Koch | 31.16667 | 39.66667 |
| *Hordeum spontaneum* K. Koch | 30.198635 | 40.067909 |
| *Hordeum spontaneum* K. Koch | 34.933333 | 29.566667 |
| *Hordeum spontaneum* K. Koch | 34.77 | 32.04 |
| *Hordeum spontaneum* K. Koch | 34.966667 | 32.516667 |
| *Hordeum spontaneum* K. Koch | 33.8833 | 35.15 |
| *Hordeum spontaneum* K. Koch | 34.65 | 37.736588 |
| *Hordeum spontaneum* K. Koch | 32.549999 | 40.723541 |
| *Hordeum spontaneum* K. Koch | 35.028892 | 29.890937 |
| *Hordeum spontaneum* K. Koch | 35.233333 | 31.783333 |
| *Hordeum spontaneum* K. Koch | 35.533333 | 32.95 |
| *Hordeum spontaneum* K. Koch | 36.565 | 35.73056 |
| *Hordeum spontaneum* K. Koch | 35 | 39 |
| *Hordeum spontaneum* K. Koch | 39.906847 | 25.727896 |
| *Hordeum spontaneum* K. Koch | 38.3561 | 34.5694 |
| *Hordeum spontaneum* K. Koch | 37.77 | 36.4839 |
| *Hordeum spontaneum* K. Koch | 38.406237 | 37.581947 |
| *Hordeum spontaneum* K. Koch | 38 | 8 |
| *Hordeum spontaneum* K. Koch | 40.3428 | 36.3803 |
| *Hordeum spontaneum* K. Koch | 41.78333 | 38.06667 |
| *Hordeum spontaneum* K. Koch | 43.333333 | 36.1 |
| *Hordeum spontaneum* K. Koch | 43.1 | 40.65 |
| *Hordeum spontaneum* K. Koch | 46.95 | 34.61667 |
| *Hordeum spontaneum* K. Koch | 45.646111 | 35.376666 |
| *Hordeum spontaneum* K. Koch | 47.2 | 38.5 |
| *Hordeum spontaneum* K. Koch | 46.9986 | 40.0017 |
| *Hordeum spontaneum* K. Koch | 48.7167 | 31.4167 |
| *Hordeum spontaneum* K. Koch | 47.716667 | 33.1 |
| *Hordeum spontaneum* K. Koch | 47.7667 | 36.2667 |
| *Hordeum spontaneum* K. Koch | 48.4703 | 38.8006 |
| *Hordeum spontaneum* K. Koch | 47.7965 | 42.4657 |
| *Hordeum spontaneum* K. Koch | 51.78333 | 29.71667 |
| *Hordeum spontaneum* K. Koch | 50.7925 | 30.35917 |
| *Hordeum spontaneum* K. Koch | 51.25 | 35.5 |
| *Hordeum spontaneum* K. Koch | 51.033333 | 48.620761 |
| *Hordeum spontaneum* K. Koch | 53.016667 | 29.5 |
| *Hordeum spontaneum* K. Koch | 53 | 32 |
| *Hordeum spontaneum* K. Koch | 57.08333 | 30.28333 |
| *Hordeum spontaneum* K. Koch | 56.3 | 38.483 |
| *Hordeum spontaneum* K. Koch | 59.48333 | 35.98333 |
| *Hordeum spontaneum* K. Koch | 58.533333 | 37.883334 |
| *Hordeum spontaneum* K. Koch | 7.456944 | 46.948611 |
| *Hordeum spontaneum* K. Koch | 62.1 | 34.65 |
| *Hordeum spontaneum* K. Koch | 62.33778 | 35.28611 |
| *Hordeum spontaneum* K. Koch | 63 | 34.5667 |
| *Hordeum spontaneum* K. Koch | 62.9 | 35.633 |
| *Hordeum spontaneum* K. Koch | 63.15 | 39.3 |
| *Hordeum spontaneum* K. Koch | 66.9 | 30.3 |
| *Hordeum spontaneum* K. Koch | 67.246949 | 37.263709 |
| *Hordeum spontaneum* K. Koch | 67.255602 | 37.569245 |
| *Hordeum spontaneum* K. Koch | 67.0858 | 40.0103 |
| *Hordeum spontaneum* K. Koch | 69 | 35.75 |
| *Hordeum spontaneum* K. Koch | 68.140581 | 38.145199 |
| *Hordeum spontaneum* K. Koch | 69.91961 | 40.64392 |
| *Hordeum spontaneum* K. Koch | 71.273443 | 29.101954 |
| *Hordeum spontaneum* K. Koch | 72.471005 | 33.884557 |
| *Hordeum spontaneum* K. Koch | 70.57972 | 37.1175 |
| *Hordeum spontaneum* K. Koch | 71.3 | 38.9 |
| *Hordeum spontaneum* K. Koch | 71.565524 | 40.405859 |
| *Hordeum spontaneum* K. Koch | 70.033 | 42.5413 |
| *Hordeum spontaneum* K. Koch | 73.144425 | 34.043933 |
| *Hordeum spontaneum* K. Koch | 73.84548 | 40.53617 |
| *Hordeum spontaneum* K. Koch | 73.657 | 43.0645 |
| *Hordeum spontaneum* K. Koch | 86.4 | 42.32 |
| *Hordeum spontaneum* K. Koch | 87.5 | 48.2 |
| *Hordeum spontaneum* K. Koch | 90 | 32 |
| *Hordeum spontaneum* K. Koch | 94.36667 | 29.41667 |
| *Hordeum spontaneum* K. Koch | 97.85 | 29.67 |
| *Hordeum spontaneum* K. Koch | 99.98 | 31.62 |
| *Hordeum stenostachys* Godr. | -100 | 47 |
| *Hordeum stenostachys* Godr. | -49.951111 | -28.33 |
| *Hordeum stenostachys* Godr. | -50.7525 | -28.592777 |
| *Hordeum stenostachys* Godr. | -54.92 | -27.09 |
| *Hordeum stenostachys* Godr. | -54.379722 | -28.396944 |
| *Hordeum stenostachys* Godr. | -54.9 | -31.35 |
| *Hordeum stenostachys* Godr. | -55.92 | -27.48 |
| *Hordeum stenostachys* Godr. | -55.13 | -27.88 |
| *Hordeum stenostachys* Godr. | -55.8175 | -30.70722 |
| *Hordeum stenostachys* Godr. | -56.18333 | -34.88333 |
| *Hordeum stenostachys* Godr. | -57.24 | -36.38 |
| *Hordeum stenostachys* Godr. | -57.43 | -37.72 |
| *Hordeum stenostachys* Godr. | -2.610524 | 53.238512 |
| *Hordeum stenostachys* Godr. | -58.72 | -26.92 |
| *Hordeum stenostachys* Godr. | -59.13 | -27.7 |
| *Hordeum stenostachys* Godr. | -58.227512 | -32.489132 |
| *Hordeum stenostachys* Godr. | -58.043928 | -32.884006 |
| *Hordeum stenostachys* Godr. | -57.828333 | -37.084722 |
| *Hordeum stenostachys* Godr. | -59.12 | -38.26 |
| *Hordeum stenostachys* Godr. | -60.48 | -28.93 |
| *Hordeum stenostachys* Godr. | -60.64 | -32.07 |
| *Hordeum stenostachys* Godr. | -62.04 | -34.66 |
| *Hordeum stenostachys* Godr. | -60.475471 | -35.089558 |
| *Hordeum stenostachys* Godr. | -62.269722 | -38.211944 |
| *Hordeum stenostachys* Godr. | -64.83 | -26.51 |
| *Hordeum stenostachys* Godr. | -63.263611 | -29.979167 |
| *Hordeum stenostachys* Godr. | -62.678696 | -30.91774 |
| *Hordeum stenostachys* Godr. | -64.54 | -33.36 |
| *Hordeum stenostachys* Godr. | -63.699622 | -35.688136 |
| *Hordeum stenostachys* Godr. | -62.78 | -39.02 |
| *Hordeum stenostachys* Godr. | -62.88 | -40.12 |
| *Hordeum stenostachys* Godr. | -65.88 | -22.73 |
| *Hordeum stenostachys* Godr. | -66.35 | -27.53 |
| *Hordeum stenostachys* Godr. | -65.41 | -32.14 |
| *Hordeum stenostachys* Godr. | -65.96799 | -32.718212 |
| *Hordeum stenostachys* Godr. | -65.08 | -36.2 |
| *Hordeum stenostachys* Godr. | -67.17 | -37.66 |
| *Hordeum stenostachys* Godr. | -65.008333 | -41.408333 |
| *Hordeum stenostachys* Godr. | -66.06 | -43.26 |
| *Hordeum stenostachys* Godr. | -68.708333 | -29.408333 |
| *Hordeum stenostachys* Godr. | -68.74 | -30.24 |
| *Hordeum stenostachys* Godr. | -68.36 | -34.94 |
| *Hordeum stenostachys* Godr. | -68.58 | -35.6 |
| *Hordeum stenostachys* Godr. | -67.713473 | -37.774957 |
| *Hordeum stenostachys* Godr. | -70.66667 | -33.33333 |
| *Hordeum stenostachys* Godr. | -71.49 | -43.02 |
| *Hordeum stenostachys* Godr. | 10.1635 | 63.3122 |
| *Hordeum stenostachys* Godr. | 3.89689 | 43.60718 |
| *Hordeum stenostachys* Godr. | 2.55194 | 46.56444 |
| *Hordeum stenostachys* Godr. | 4.76118 | 52.50852 |
| *Hordeum stenostachys* Godr. | 24.375 | -28.375 |
| *Hordeum stenostachys* Godr. | 24.875 | -31.375 |
| *Hordeum stenostachys* Godr. | 23 | -33 |
| *Hordeum stenostachys* Godr. | 26 | -30 |
| *Hordeum stenostachys* Godr. | 25.025 | -31.465556 |
| *Hordeum stenostachys* Godr. | 6.21502 | 52.27361 |
| *Hordeum stenostachys* Godr. | 9.98 | 53.51417 |

Supplementary Table 2. Description of environmental variables used in niche modeling.

| Environmental Variable | Description | Environmental Variable | Description |
| --- | --- | --- | --- |
| Bio1 | Annual Mean Temperature | Bio11 | Mean Temperature of Coldest Quarter |
| Bio2 | Mean Diurnal Range | Bio12 | Annual Precipitation |
| Bio3 | Isothermality | Bio13 | Precipitation of Wettest Month |
| Bio4 | Temperature Seasonality | Bio14 | Precipitation of Driest Month |
| Bio5 | Max Temperature of Warmest Month | Bio15 | Precipitation Seasonality |
| Bio6 | Min Temperature of Coldest Month | Bio16 | Precipitation of Wettest Quarter |
| Bio7 | Temperature Annual Range | Bio17 | Precipitation of Driest Quarter |
| Bio8 | Mean Temperature of Wettest Quarter | Bio18 | Precipitation of Warmest Quarter |
| Bio9 | Mean Temperature of Driest Quarter | Bio19 | Precipitation of Coldest Quarter |
| Bio10 | Mean Temperature of Warmest Quarter | Terrain variable | Elevation |

Supplementary Table 3. Preliminary Screening of Bioclimatic and Terrain Environmental Variables

| Environmental Variable | Description |
| --- | --- |
| Bio2 | Mean Diurnal Range |
| Bio3 | Isothermality |
| Bio8 | Mean Temperature of Wettest Quarter |
| Bio9 | Mean Temperature of Driest Quarter |
| Bio13 | Precipitation of Wettest Month |
| Bio14 | Precipitation of Driest Month |
| Bio15 | Precipitation Seasonality |
| Bio18 | Precipitation of Warmest Quarter |
| Bio19 | Precipitation of Coldest Quarter |
| Elevation | Terrain variable |

Supplementary Table 4. Optimal parameter selection for MaxEnt niche models of 13 *Hordeum* species.

| Specise | Life History | TSS | TSS Average | FC | RM | AUC |
| --- | --- | --- | --- | --- | --- | --- |
| *Hordeum agriocrithon* A. E. Åberg | Annual | 0.886 | 0.889 | LQH | 2.5 | 0.906 |
|  |  | 0.880 |  |  |  |  |
|  |  | 0.833 |  |  |  |  |
|  |  | 0.892 |  |  |  |  |
|  |  | 0.893 |  |  |  |  |
|  |  | 0.905 |  |  |  |  |
|  |  | 0.905 |  |  |  |  |
|  |  | 0.905 |  |  |  |  |
|  |  | 0.905 |  |  |  |  |
|  |  | 0.883 |  |  |  |  |
| *Hordeum bogdanii* Wilensky | Perennial | 0.768 | 0.756 | LQHP | 2.5 | 0.927 |
|  |  | 0.743 |  |  |  |  |
|  |  | 0.758 |  |  |  |  |
|  |  | 0.757 |  |  |  |  |
|  |  | 0.772 |  |  |  |  |
|  |  | 0.757 |  |  |  |  |
|  |  | 0.775 |  |  |  |  |
|  |  | 0.744 |  |  |  |  |
|  |  | 0.748 |  |  |  |  |
|  |  | 0.742 |  |  |  |  |
| *Hordeum brevisubulatum* (Trin.) Link | Perennial | 0.695 | 0.710 | LQHP | 0.5 | 0.902 |
|  |  | 0.709 |  |  |  |  |
|  |  | 0.715 |  |  |  |  |
|  |  | 0.715 |  |  |  |  |
|  |  | 0.705 |  |  |  |  |
|  |  | 0.703 |  |  |  |  |
|  |  | 0.714 |  |  |  |  |
|  |  | 0.707 |  |  |  |  |
|  |  | 0.721 |  |  |  |  |
|  |  | 0.715 |  |  |  |  |
| *Hordeum bulbosum* L. | Perennial | 0.774 | 0.770 | LQH | 2.0 | 0.936 |
|  |  | 0.788 |  |  |  |  |
|  |  | 0.784 |  |  |  |  |
|  |  | 0.764 |  |  |  |  |
|  |  | 0.766 |  |  |  |  |
|  |  | 0.769 |  |  |  |  |
|  |  | 0.759 |  |  |  |  |
|  |  | 0.769 |  |  |  |  |
|  |  | 0.762 |  |  |  |  |
|  |  | 0.768 |  |  |  |  |
| *Hordeum californicum* Covas & Stebbins | Annual | 0.792 | 0.798 | H | 2.5 | 0.927 |
|  |  | 0.808 |  |  |  |  |
|  |  | 0.788 |  |  |  |  |
|  |  | 0.769 |  |  |  |  |
|  |  | 0.825 |  |  |  |  |
|  |  | 0.807 |  |  |  |  |
|  |  | 0.823 |  |  |  |  |
|  |  | 0.809 |  |  |  |  |
|  |  | 0.779 |  |  |  |  |
|  |  | 0.775 |  |  |  |  |
| *Hordeum chilense* Roem. & Schult. | Annual | 0.714 | 0.714 | LQHPT | 2.5 | 0.830 |
|  |  | 0.650 |  |  |  |  |
|  |  | 0.756 |  |  |  |  |
|  |  | 0.714 |  |  |  |  |
|  |  | 0.680 |  |  |  |  |
|  |  | 0.712 |  |  |  |  |
|  |  | 0.742 |  |  |  |  |
|  |  | 0.711 |  |  |  |  |
|  |  | 0.749 |  |  |  |  |
|  |  | 0.712 |  |  |  |  |
| *Hordeum marinum* Huds. | Annual | 0.769 | 0.770 | LQHP | 1.0 | 0.935 |
|  |  | 0.766 |  |  |  |  |
|  |  | 0.768 |  |  |  |  |
|  |  | 0.779 |  |  |  |  |
|  |  | 0.769 |  |  |  |  |
|  |  | 0.770 |  |  |  |  |
|  |  | 0.773 |  |  |  |  |
|  |  | 0.768 |  |  |  |  |
|  |  | 0.764 |  |  |  |  |
|  |  | 0.771 |  |  |  |  |
| *Hordeum murinum* L. | Annual | 0.662 | 0.660 | LQH | 2.0 | 0.875 |
|  |  | 0.661 |  |  |  |  |
|  |  | 0.662 |  |  |  |  |
|  |  | 0.656 |  |  |  |  |
|  |  | 0.659 |  |  |  |  |
|  |  | 0.657 |  |  |  |  |
|  |  | 0.658 |  |  |  |  |
|  |  | 0.662 |  |  |  |  |
|  |  | 0.660 |  |  |  |  |
|  |  | 0.658 |  |  |  |  |
| *Hordeum muticum* J.Presl | Perennial | 0.733 | 0.693 | LQ | 2.0 | 0.894 |
|  |  | 0.669 |  |  |  |  |
|  |  | 0.629 |  |  |  |  |
|  |  | 0.770 |  |  |  |  |
|  |  | 0.694 |  |  |  |  |
|  |  | 0.633 |  |  |  |  |
|  |  | 0.656 |  |  |  |  |
|  |  | 0.749 |  |  |  |  |
|  |  | 0.713 |  |  |  |  |
|  |  | 0.685 |  |  |  |  |
| *Hordeum pusillum* Nutt. | Annual | 0.822 | 0.821 | LQHP | 1.5 | 0.944 |
|  |  | 0.821 |  |  |  |  |
|  |  | 0.824 |  |  |  |  |
|  |  | 0.821 |  |  |  |  |
|  |  | 0.814 |  |  |  |  |
|  |  | 0.820 |  |  |  |  |
|  |  | 0.819 |  |  |  |  |
|  |  | 0.828 |  |  |  |  |
|  |  | 0.821 |  |  |  |  |
|  |  | 0.824 |  |  |  |  |
| *Hordeum roshevitzii* Bowden | Perennial | 0.809 | 0.814 | LQHPT | 3.0 | 0.940 |
|  |  | 0.808 |  |  |  |  |
|  |  | 0.825 |  |  |  |  |
|  |  | 0.815 |  |  |  |  |
|  |  | 0.807 |  |  |  |  |
|  |  | 0.799 |  |  |  |  |
|  |  | 0.821 |  |  |  |  |
|  |  | 0.823 |  |  |  |  |
|  |  | 0.841 |  |  |  |  |
|  |  | 0.788 |  |  |  |  |
| *Hordeum spontaneum* K. Koch | Annual | 0.720 | 0.719 | H | 1.0 | 0.901 |
|  |  | 0.706 |  |  |  |  |
|  |  | 0.723 |  |  |  |  |
|  |  | 0.739 |  |  |  |  |
|  |  | 0.725 |  |  |  |  |
|  |  | 0.725 |  |  |  |  |
|  |  | 0.718 |  |  |  |  |
|  |  | 0.690 |  |  |  |  |
|  |  | 0.734 |  |  |  |  |
|  |  | 0.711 |  |  |  |  |
| *Hordeum stenostachys* Godr. | Perennial | 0.825 | 0.821 | LQHPT | 2.5 | 0.904 |
|  |  | 0.850 |  |  |  |  |
|  |  | 0.803 |  |  |  |  |
|  |  | 0.813 |  |  |  |  |
|  |  | 0.833 |  |  |  |  |
|  |  | 0.818 |  |  |  |  |
|  |  | 0.815 |  |  |  |  |
|  |  | 0.802 |  |  |  |  |
|  |  | 0.816 |  |  |  |  |
|  |  | 0.832 |  |  |  |  |

Supplementary Table 5. Statistical table of potential suitable habitat areas at different levels for *Hordeum* species under different climate scenarios.

| Specise | Scenario | period | Level | Level Name | Area (×10^6^km^2^) |
| --- | --- | --- | --- | --- | --- |
| *Hordeum agriocrithon* A. E. Åberg | current | current | 1 | Unsuitable | 46.96 |
|  |  |  | 2 | Low suitability | 26.33 |
|  |  |  | 3 | Medium suitability | 28.74 |
|  |  |  | 4 | High suitability | 20.42 |
|  |  |  | 5 | Very high suitability | 14.34 |
|  |  |  | Total | 2-5 | 89.83 |
|  | ssp126 | 2041-2060 | 1 | Unsuitable | 13.00 |
|  |  |  | 2 | Low suitability | 42.34 |
|  |  |  | 3 | Medium suitability | 68.53 |
|  |  |  | 4 | High suitability | 8.79 |
|  |  |  | 5 | Very high suitability | 4.14 |
|  |  |  | Total | 2-5 | 123.80 |
|  |  | 2061-2080 | 1 | Unsuitable | 13.29 |
|  |  |  | 2 | Low suitability | 42.14 |
|  |  |  | 3 | Medium suitability | 68.54 |
|  |  |  | 4 | High suitability | 8.67 |
|  |  |  | 5 | Very high suitability | 4.17 |
|  |  |  | Total | 2-5 | 123.51 |
|  |  | 2081-2100 | 1 | Unsuitable | 12.94 |
|  |  |  | 2 | Low suitability | 41.76 |
|  |  |  | 3 | Medium suitability | 68.55 |
|  |  |  | 4 | High suitability | 9.38 |
|  |  |  | 5 | Very high suitability | 4.17 |
|  |  |  | Total | 2-5 | 123.86 |
|  | ssp245 | 2041-2060 | 1 | Unsuitable | 14.24 |
|  |  |  | 2 | Low suitability | 42.12 |
|  |  |  | 3 | Medium suitability | 67.79 |
|  |  |  | 4 | High suitability | 8.52 |
|  |  |  | 5 | Very high suitability | 4.13 |
|  |  |  | Total | 2-5 | 122.57 |
|  |  | 2061-2080 | 1 | Unsuitable | 15.42 |
|  |  |  | 2 | Low suitability | 42.91 |
|  |  |  | 3 | Medium suitability | 66.38 |
|  |  |  | 4 | High suitability | 7.99 |
|  |  |  | 5 | Very high suitability | 4.11 |
|  |  |  | Total | 2-5 | 121.39 |
|  |  | 2081-2100 | 1 | Unsuitable | 16.06 |
|  |  |  | 2 | Low suitability | 43.60 |
|  |  |  | 3 | Medium suitability | 65.43 |
|  |  |  | 4 | High suitability | 7.61 |
|  |  |  | 5 | Very high suitability | 4.12 |
|  |  |  | Total | 2-5 | 120.74 |
|  | ssp370 | 2041-2060 | 1 | Unsuitable | 14.88 |
|  |  |  | 2 | Low suitability | 42.78 |
|  |  |  | 3 | Medium suitability | 67.09 |
|  |  |  | 4 | High suitability | 7.89 |
|  |  |  | 5 | Very high suitability | 4.16 |
|  |  |  | Total | 2-5 | 121.92 |
|  |  | 2061-2080 | 1 | Unsuitable | 17.45 |
|  |  |  | 2 | Low suitability | 42.93 |
|  |  |  | 3 | Medium suitability | 65.01 |
|  |  |  | 4 | High suitability | 7.34 |
|  |  |  | 5 | Very high suitability | 4.08 |
|  |  |  | Total | 2-5 | 119.36 |
|  |  | 2081-2100 | 1 | Unsuitable | 18.94 |
|  |  |  | 2 | Low suitability | 44.18 |
|  |  |  | 3 | Medium suitability | 62.67 |
|  |  |  | 4 | High suitability | 6.98 |
|  |  |  | 5 | Very high suitability | 4.04 |
|  |  |  | Total | 2-5 | 117.87 |
|  | ssp585 | 2041-2060 | 1 | Unsuitable | 15.00 |
|  |  |  | 2 | Low suitability | 43.04 |
|  |  |  | 3 | Medium suitability | 66.51 |
|  |  |  | 4 | High suitability | 8.12 |
|  |  |  | 5 | Very high suitability | 4.13 |
|  |  |  | Total | 2-5 | 121.80 |
|  |  | 2061-2080 | 1 | Unsuitable | 17.47 |
|  |  |  | 2 | Low suitability | 44.22 |
|  |  |  | 3 | Medium suitability | 63.81 |
|  |  |  | 4 | High suitability | 7.25 |
|  |  |  | 5 | Very high suitability | 4.05 |
|  |  |  | Total | 2-5 | 119.33 |
|  |  | 2081-2100 | 1 | Unsuitable | 19.54 |
|  |  |  | 2 | Low suitability | 44.89 |
|  |  |  | 3 | Medium suitability | 61.50 |
|  |  |  | 4 | High suitability | 6.89 |
|  |  |  | 5 | Very high suitability | 3.99 |
|  |  |  | Total | 2-5 | 117.27 |
| *Hordeum bogdanii* Wilensky | current | current | 1 | Unsuitable | 84.03 |
|  |  |  | 2 | Low suitability | 27.12 |
|  |  |  | 3 | Medium suitability | 13.15 |
|  |  |  | 4 | High suitability | 6.24 |
|  |  |  | 5 | Very high suitability | 6.26 |
|  |  |  | Total | 2-5 | 52.78 |
|  | ssp126 | 2041-2060 | 1 | Unsuitable | 119.09 |
|  |  |  | 2 | Low suitability | 5.35 |
|  |  |  | 3 | Medium suitability | 2.25 |
|  |  |  | 4 | High suitability | 2.85 |
|  |  |  | 5 | Very high suitability | 7.28 |
|  |  |  | Total | 2-5 | 17.72 |
|  |  | 2061-2080 | 1 | Unsuitable | 119.45 |
|  |  |  | 2 | Low suitability | 5.28 |
|  |  |  | 3 | Medium suitability | 2.20 |
|  |  |  | 4 | High suitability | 2.87 |
|  |  |  | 5 | Very high suitability | 7.01 |
|  |  |  | Total | 2-5 | 17.35 |
|  |  | 2081-2100 | 1 | Unsuitable | 119.34 |
|  |  |  | 2 | Low suitability | 5.25 |
|  |  |  | 3 | Medium suitability | 2.28 |
|  |  |  | 4 | High suitability | 2.86 |
|  |  |  | 5 | Very high suitability | 7.08 |
|  |  |  | Total | 2-5 | 17.47 |
|  | ssp245 | 2041-2060 | 1 | Unsuitable | 119.12 |
|  |  |  | 2 | Low suitability | 5.31 |
|  |  |  | 3 | Medium suitability | 2.37 |
|  |  |  | 4 | High suitability | 2.95 |
|  |  |  | 5 | Very high suitability | 7.06 |
|  |  |  | Total | 2-5 | 17.69 |
|  |  | 2061-2080 | 1 | Unsuitable | 119.70 |
|  |  |  | 2 | Low suitability | 5.10 |
|  |  |  | 3 | Medium suitability | 2.38 |
|  |  |  | 4 | High suitability | 2.92 |
|  |  |  | 5 | Very high suitability | 6.71 |
|  |  |  | Total | 2-5 | 17.11 |
|  |  | 2081-2100 | 1 | Unsuitable | 119.88 |
|  |  |  | 2 | Low suitability | 5.11 |
|  |  |  | 3 | Medium suitability | 2.25 |
|  |  |  | 4 | High suitability | 2.98 |
|  |  |  | 5 | Very high suitability | 6.60 |
|  |  |  | Total | 2-5 | 16.93 |
|  | ssp370 | 2041-2060 | 1 | Unsuitable | 119.51 |
|  |  |  | 2 | Low suitability | 5.24 |
|  |  |  | 3 | Medium suitability | 2.29 |
|  |  |  | 4 | High suitability | 3.01 |
|  |  |  | 5 | Very high suitability | 6.76 |
|  |  |  | Total | 2-5 | 17.30 |
|  |  | 2061-2080 | 1 | Unsuitable | 119.84 |
|  |  |  | 2 | Low suitability | 5.15 |
|  |  |  | 3 | Medium suitability | 2.32 |
|  |  |  | 4 | High suitability | 2.99 |
|  |  |  | 5 | Very high suitability | 6.51 |
|  |  |  | Total | 2-5 | 16.97 |
|  |  | 2081-2100 | 1 | Unsuitable | 120.36 |
|  |  |  | 2 | Low suitability | 5.09 |
|  |  |  | 3 | Medium suitability | 2.18 |
|  |  |  | 4 | High suitability | 2.96 |
|  |  |  | 5 | Very high suitability | 6.21 |
|  |  |  | Total | 2-5 | 16.45 |
|  | ssp585 | 2041-2060 | 1 | Unsuitable | 119.52 |
|  |  |  | 2 | Low suitability | 5.33 |
|  |  |  | 3 | Medium suitability | 2.37 |
|  |  |  | 4 | High suitability | 2.95 |
|  |  |  | 5 | Very high suitability | 6.64 |
|  |  |  | Total | 2-5 | 17.29 |
|  |  | 2061-2080 | 1 | Unsuitable | 119.52 |
|  |  |  | 2 | Low suitability | 5.47 |
|  |  |  | 3 | Medium suitability | 2.51 |
|  |  |  | 4 | High suitability | 3.09 |
|  |  |  | 5 | Very high suitability | 6.22 |
|  |  |  | Total | 2-5 | 17.29 |
|  |  | 2081-2100 | 1 | Unsuitable | 119.67 |
|  |  |  | 2 | Low suitability | 5.54 |
|  |  |  | 3 | Medium suitability | 2.39 |
|  |  |  | 4 | High suitability | 3.08 |
|  |  |  | 5 | Very high suitability | 6.13 |
|  |  |  | Total | 2-5 | 17.13 |
| *Hordeum brevisubulatum* (Trin.) Link | current | current | 1 | Unsuitable | 89.49 |
|  |  |  | 2 | Low suitability | 20.79 |
|  |  |  | 3 | Medium suitability | 11.33 |
|  |  |  | 4 | High suitability | 8.41 |
|  |  |  | 5 | Very high suitability | 6.79 |
|  |  |  | Total | 2-5 | 47.32 |
|  | ssp126 | 2041-2060 | 1 | Unsuitable | 132.09 |
|  |  |  | 2 | Low suitability | 3.86 |
|  |  |  | 3 | Medium suitability | 0.68 |
|  |  |  | 4 | High suitability | 0.07 |
|  |  |  | 5 | Very high suitability | 0.11 |
|  |  |  | Total | 2-5 | 4.71 |
|  |  | 2061-2080 | 1 | Unsuitable | 132.03 |
|  |  |  | 2 | Low suitability | 3.93 |
|  |  |  | 3 | Medium suitability | 0.67 |
|  |  |  | 4 | High suitability | 0.07 |
|  |  |  | 5 | Very high suitability | 0.10 |
|  |  |  | Total | 2-5 | 4.77 |
|  |  | 2081-2100 | 1 | Unsuitable | 132.09 |
|  |  |  | 2 | Low suitability | 3.95 |
|  |  |  | 3 | Medium suitability | 0.60 |
|  |  |  | 4 | High suitability | 0.06 |
|  |  |  | 5 | Very high suitability | 0.10 |
|  |  |  | Total | 2-5 | 4.71 |
|  | ssp245 | 2041-2060 | 1 | Unsuitable | 132.10 |
|  |  |  | 2 | Low suitability | 3.85 |
|  |  |  | 3 | Medium suitability | 0.67 |
|  |  |  | 4 | High suitability | 0.08 |
|  |  |  | 5 | Very high suitability | 0.11 |
|  |  |  | Total | 2-5 | 4.70 |
|  |  | 2061-2080 | 1 | Unsuitable | 132.07 |
|  |  |  | 2 | Low suitability | 3.91 |
|  |  |  | 3 | Medium suitability | 0.65 |
|  |  |  | 4 | High suitability | 0.07 |
|  |  |  | 5 | Very high suitability | 0.11 |
|  |  |  | Total | 2-5 | 4.73 |
|  |  | 2081-2100 | 1 | Unsuitable | 131.93 |
|  |  |  | 2 | Low suitability | 3.99 |
|  |  |  | 3 | Medium suitability | 0.70 |
|  |  |  | 4 | High suitability | 0.07 |
|  |  |  | 5 | Very high suitability | 0.12 |
|  |  |  | Total | 2-5 | 4.87 |
|  | ssp370 | 2041-2060 | 1 | Unsuitable | 131.95 |
|  |  |  | 2 | Low suitability | 3.95 |
|  |  |  | 3 | Medium suitability | 0.72 |
|  |  |  | 4 | High suitability | 0.07 |
|  |  |  | 5 | Very high suitability | 0.11 |
|  |  |  | Total | 2-5 | 4.85 |
|  |  | 2061-2080 | 1 | Unsuitable | 132.03 |
|  |  |  | 2 | Low suitability | 3.93 |
|  |  |  | 3 | Medium suitability | 0.66 |
|  |  |  | 4 | High suitability | 0.07 |
|  |  |  | 5 | Very high suitability | 0.12 |
|  |  |  | Total | 2-5 | 4.78 |
|  |  | 2081-2100 | 1 | Unsuitable | 131.86 |
|  |  |  | 2 | Low suitability | 4.06 |
|  |  |  | 3 | Medium suitability | 0.69 |
|  |  |  | 4 | High suitability | 0.07 |
|  |  |  | 5 | Very high suitability | 0.13 |
|  |  |  | Total | 2-5 | 4.94 |
|  | ssp585 | 2041-2060 | 1 | Unsuitable | 131.99 |
|  |  |  | 2 | Low suitability | 3.93 |
|  |  |  | 3 | Medium suitability | 0.70 |
|  |  |  | 4 | High suitability | 0.07 |
|  |  |  | 5 | Very high suitability | 0.12 |
|  |  |  | Total | 2-5 | 4.82 |
|  |  | 2061-2080 | 1 | Unsuitable | 131.75 |
|  |  |  | 2 | Low suitability | 4.04 |
|  |  |  | 3 | Medium suitability | 0.81 |
|  |  |  | 4 | High suitability | 0.10 |
|  |  |  | 5 | Very high suitability | 0.11 |
|  |  |  | Total | 2-5 | 5.06 |
|  |  | 2081-2100 | 1 | Unsuitable | 131.78 |
|  |  |  | 2 | Low suitability | 4.10 |
|  |  |  | 3 | Medium suitability | 0.73 |
|  |  |  | 4 | High suitability | 0.07 |
|  |  |  | 5 | Very high suitability | 0.12 |
|  |  |  | Total | 2-5 | 5.03 |
| *Hordeum bulbosum* L. | current | current | 1 | Unsuitable | 103.78 |
|  |  |  | 2 | Low suitability | 16.94 |
|  |  |  | 3 | Medium suitability | 7.06 |
|  |  |  | 4 | High suitability | 3.99 |
|  |  |  | 5 | Very high suitability | 5.03 |
|  |  |  | Total | 2-5 | 33.02 |
|  | ssp126 | 2041-2060 | 1 | Unsuitable | 127.44 |
|  |  |  | 2 | Low suitability | 6.51 |
|  |  |  | 3 | Medium suitability | 1.69 |
|  |  |  | 4 | High suitability | 0.66 |
|  |  |  | 5 | Very high suitability | 0.51 |
|  |  |  | Total | 2-5 | 9.37 |
|  |  | 2061-2080 | 1 | Unsuitable | 127.56 |
|  |  |  | 2 | Low suitability | 6.55 |
|  |  |  | 3 | Medium suitability | 1.62 |
|  |  |  | 4 | High suitability | 0.51 |
|  |  |  | 5 | Very high suitability | 0.58 |
|  |  |  | Total | 2-5 | 9.25 |
|  |  | 2081-2100 | 1 | Unsuitable | 127.68 |
|  |  |  | 2 | Low suitability | 6.54 |
|  |  |  | 3 | Medium suitability | 1.52 |
|  |  |  | 4 | High suitability | 0.55 |
|  |  |  | 5 | Very high suitability | 0.53 |
|  |  |  | Total | 2-5 | 9.13 |
|  | ssp245 | 2041-2060 | 1 | Unsuitable | 127.18 |
|  |  |  | 2 | Low suitability | 6.72 |
|  |  |  | 3 | Medium suitability | 1.81 |
|  |  |  | 4 | High suitability | 0.57 |
|  |  |  | 5 | Very high suitability | 0.54 |
|  |  |  | Total | 2-5 | 9.63 |
|  |  | 2061-2080 | 1 | Unsuitable | 127.27 |
|  |  |  | 2 | Low suitability | 6.55 |
|  |  |  | 3 | Medium suitability | 1.79 |
|  |  |  | 4 | High suitability | 0.54 |
|  |  |  | 5 | Very high suitability | 0.65 |
|  |  |  | Total | 2-5 | 9.54 |
|  |  | 2081-2100 | 1 | Unsuitable | 127.25 |
|  |  |  | 2 | Low suitability | 6.57 |
|  |  |  | 3 | Medium suitability | 1.76 |
|  |  |  | 4 | High suitability | 0.70 |
|  |  |  | 5 | Very high suitability | 0.53 |
|  |  |  | Total | 2-5 | 9.56 |
|  | ssp370 | 2041-2060 | 1 | Unsuitable | 127.24 |
|  |  |  | 2 | Low suitability | 6.82 |
|  |  |  | 3 | Medium suitability | 1.61 |
|  |  |  | 4 | High suitability | 0.58 |
|  |  |  | 5 | Very high suitability | 0.55 |
|  |  |  | Total | 2-5 | 9.57 |
|  |  | 2061-2080 | 1 | Unsuitable | 127.05 |
|  |  |  | 2 | Low suitability | 6.74 |
|  |  |  | 3 | Medium suitability | 1.75 |
|  |  |  | 4 | High suitability | 0.69 |
|  |  |  | 5 | Very high suitability | 0.59 |
|  |  |  | Total | 2-5 | 9.76 |
|  |  | 2081-2100 | 1 | Unsuitable | 127.12 |
|  |  |  | 2 | Low suitability | 6.69 |
|  |  |  | 3 | Medium suitability | 1.80 |
|  |  |  | 4 | High suitability | 0.68 |
|  |  |  | 5 | Very high suitability | 0.52 |
|  |  |  | Total | 2-5 | 9.68 |
|  | ssp585 | 2041-2060 | 1 | Unsuitable | 127.28 |
|  |  |  | 2 | Low suitability | 6.72 |
|  |  |  | 3 | Medium suitability | 1.66 |
|  |  |  | 4 | High suitability | 0.59 |
|  |  |  | 5 | Very high suitability | 0.55 |
|  |  |  | Total | 2-5 | 9.52 |
|  |  | 2061-2080 | 1 | Unsuitable | 126.82 |
|  |  |  | 2 | Low suitability | 6.90 |
|  |  |  | 3 | Medium suitability | 1.78 |
|  |  |  | 4 | High suitability | 0.71 |
|  |  |  | 5 | Very high suitability | 0.59 |
|  |  |  | Total | 2-5 | 9.99 |
|  |  | 2081-2100 | 1 | Unsuitable | 126.96 |
|  |  |  | 2 | Low suitability | 6.68 |
|  |  |  | 3 | Medium suitability | 1.82 |
|  |  |  | 4 | High suitability | 0.69 |
|  |  |  | 5 | Very high suitability | 0.65 |
|  |  |  | Total | 2-5 | 9.85 |
| *Hordeum californicum* Covas & Stebbins | current | current | 1 | Unsuitable | 37.52 |
|  |  |  | 2 | Low suitability | 58.70 |
|  |  |  | 3 | Medium suitability | 16.23 |
|  |  |  | 4 | High suitability | 10.77 |
|  |  |  | 5 | Very high suitability | 13.58 |
|  |  |  | Total | 2-5 | 99.28 |
|  | ssp126 | 2041-2060 | 1 | Unsuitable | 119.30 |
|  |  |  | 2 | Low suitability | 1.50 |
|  |  |  | 3 | Medium suitability | 2.20 |
|  |  |  | 4 | High suitability | 2.29 |
|  |  |  | 5 | Very high suitability | 11.52 |
|  |  |  | Total | 2-5 | 17.50 |
|  |  | 2061-2080 | 1 | Unsuitable | 119.46 |
|  |  |  | 2 | Low suitability | 1.56 |
|  |  |  | 3 | Medium suitability | 2.19 |
|  |  |  | 4 | High suitability | 2.29 |
|  |  |  | 5 | Very high suitability | 11.31 |
|  |  |  | Total | 2-5 | 17.35 |
|  |  | 2081-2100 | 1 | Unsuitable | 119.56 |
|  |  |  | 2 | Low suitability | 1.52 |
|  |  |  | 3 | Medium suitability | 2.16 |
|  |  |  | 4 | High suitability | 2.25 |
|  |  |  | 5 | Very high suitability | 11.31 |
|  |  |  | Total | 2-5 | 17.24 |
|  | ssp245 | 2041-2060 | 1 | Unsuitable | 119.11 |
|  |  |  | 2 | Low suitability | 1.57 |
|  |  |  | 3 | Medium suitability | 2.17 |
|  |  |  | 4 | High suitability | 2.21 |
|  |  |  | 5 | Very high suitability | 11.75 |
|  |  |  | Total | 2-5 | 17.70 |
|  |  | 2061-2080 | 1 | Unsuitable | 119.22 |
|  |  |  | 2 | Low suitability | 1.57 |
|  |  |  | 3 | Medium suitability | 2.19 |
|  |  |  | 4 | High suitability | 2.27 |
|  |  |  | 5 | Very high suitability | 11.56 |
|  |  |  | Total | 2-5 | 17.58 |
|  |  | 2081-2100 | 1 | Unsuitable | 119.10 |
|  |  |  | 2 | Low suitability | 1.53 |
|  |  |  | 3 | Medium suitability | 2.23 |
|  |  |  | 4 | High suitability | 2.30 |
|  |  |  | 5 | Very high suitability | 11.65 |
|  |  |  | Total | 2-5 | 17.70 |
|  | ssp370 | 2041-2060 | 1 | Unsuitable | 119.34 |
|  |  |  | 2 | Low suitability | 1.59 |
|  |  |  | 3 | Medium suitability | 2.15 |
|  |  |  | 4 | High suitability | 2.21 |
|  |  |  | 5 | Very high suitability | 11.52 |
|  |  |  | Total | 2-5 | 17.47 |
|  |  | 2061-2080 | 1 | Unsuitable | 119.25 |
|  |  |  | 2 | Low suitability | 1.55 |
|  |  |  | 3 | Medium suitability | 2.20 |
|  |  |  | 4 | High suitability | 2.29 |
|  |  |  | 5 | Very high suitability | 11.51 |
|  |  |  | Total | 2-5 | 17.55 |
|  |  | 2081-2100 | 1 | Unsuitable | 119.07 |
|  |  |  | 2 | Low suitability | 1.64 |
|  |  |  | 3 | Medium suitability | 2.25 |
|  |  |  | 4 | High suitability | 2.42 |
|  |  |  | 5 | Very high suitability | 11.43 |
|  |  |  | Total | 2-5 | 17.74 |
|  | ssp585 | 2041-2060 | 1 | Unsuitable | 119.31 |
|  |  |  | 2 | Low suitability | 1.55 |
|  |  |  | 3 | Medium suitability | 2.15 |
|  |  |  | 4 | High suitability | 2.22 |
|  |  |  | 5 | Very high suitability | 11.57 |
|  |  |  | Total | 2-5 | 17.50 |
|  |  | 2061-2080 | 1 | Unsuitable | 118.96 |
|  |  |  | 2 | Low suitability | 1.63 |
|  |  |  | 3 | Medium suitability | 2.17 |
|  |  |  | 4 | High suitability | 2.24 |
|  |  |  | 5 | Very high suitability | 11.81 |
|  |  |  | Total | 2-5 | 17.85 |
|  |  | 2081-2100 | 1 | Unsuitable | 119.00 |
|  |  |  | 2 | Low suitability | 1.60 |
|  |  |  | 3 | Medium suitability | 2.14 |
|  |  |  | 4 | High suitability | 2.41 |
|  |  |  | 5 | Very high suitability | 11.65 |
|  |  |  | Total | 2-5 | 17.80 |
| *Hordeum chilense* Roem. & Schult. | current | current | 1 | Unsuitable | 2.38 |
|  |  |  | 2 | Low suitability | 37.39 |
|  |  |  | 3 | Medium suitability | 58.01 |
|  |  |  | 4 | High suitability | 24.41 |
|  |  |  | 5 | Very high suitability | 14.62 |
|  |  |  | Total | 2-5 | 134.42 |
|  | ssp126 | 2041-2060 | 1 | Unsuitable | 91.08 |
|  |  |  | 2 | Low suitability | 29.66 |
|  |  |  | 3 | Medium suitability | 4.50 |
|  |  |  | 4 | High suitability | 5.48 |
|  |  |  | 5 | Very high suitability | 6.09 |
|  |  |  | Total | 2-5 | 45.72 |
|  |  | 2061-2080 | 1 | Unsuitable | 90.90 |
|  |  |  | 2 | Low suitability | 30.05 |
|  |  |  | 3 | Medium suitability | 4.58 |
|  |  |  | 4 | High suitability | 5.34 |
|  |  |  | 5 | Very high suitability | 5.93 |
|  |  |  | Total | 2-5 | 45.90 |
|  |  | 2081-2100 | 1 | Unsuitable | 90.36 |
|  |  |  | 2 | Low suitability | 30.66 |
|  |  |  | 3 | Medium suitability | 4.37 |
|  |  |  | 4 | High suitability | 5.43 |
|  |  |  | 5 | Very high suitability | 5.98 |
|  |  |  | Total | 2-5 | 46.45 |
|  | ssp245 | 2041-2060 | 1 | Unsuitable | 90.60 |
|  |  |  | 2 | Low suitability | 30.02 |
|  |  |  | 3 | Medium suitability | 4.62 |
|  |  |  | 4 | High suitability | 5.43 |
|  |  |  | 5 | Very high suitability | 6.15 |
|  |  |  | Total | 2-5 | 46.21 |
|  |  | 2061-2080 | 1 | Unsuitable | 91.20 |
|  |  |  | 2 | Low suitability | 29.52 |
|  |  |  | 3 | Medium suitability | 4.44 |
|  |  |  | 4 | High suitability | 5.55 |
|  |  |  | 5 | Very high suitability | 6.09 |
|  |  |  | Total | 2-5 | 45.60 |
|  |  | 2081-2100 | 1 | Unsuitable | 91.61 |
|  |  |  | 2 | Low suitability | 29.03 |
|  |  |  | 3 | Medium suitability | 4.54 |
|  |  |  | 4 | High suitability | 5.52 |
|  |  |  | 5 | Very high suitability | 6.11 |
|  |  |  | Total | 2-5 | 45.20 |
|  | ssp370 | 2041-2060 | 1 | Unsuitable | 91.02 |
|  |  |  | 2 | Low suitability | 29.77 |
|  |  |  | 3 | Medium suitability | 4.55 |
|  |  |  | 4 | High suitability | 5.53 |
|  |  |  | 5 | Very high suitability | 5.95 |
|  |  |  | Total | 2-5 | 45.79 |
|  |  | 2061-2080 | 1 | Unsuitable | 91.40 |
|  |  |  | 2 | Low suitability | 29.39 |
|  |  |  | 3 | Medium suitability | 4.50 |
|  |  |  | 4 | High suitability | 5.52 |
|  |  |  | 5 | Very high suitability | 6.00 |
|  |  |  | Total | 2-5 | 45.41 |
|  |  | 2081-2100 | 1 | Unsuitable | 90.92 |
|  |  |  | 2 | Low suitability | 29.72 |
|  |  |  | 3 | Medium suitability | 4.56 |
|  |  |  | 4 | High suitability | 5.53 |
|  |  |  | 5 | Very high suitability | 6.07 |
|  |  |  | Total | 2-5 | 45.89 |
|  | ssp585 | 2041-2060 | 1 | Unsuitable | 90.42 |
|  |  |  | 2 | Low suitability | 30.39 |
|  |  |  | 3 | Medium suitability | 4.53 |
|  |  |  | 4 | High suitability | 5.47 |
|  |  |  | 5 | Very high suitability | 6.01 |
|  |  |  | Total | 2-5 | 46.39 |
|  |  |  | 1 | Unsuitable | 90.82 |
|  |  |  | 2 | Low suitability | 29.75 |
|  |  |  | 3 | Medium suitability | 4.78 |
|  |  |  | 4 | High suitability | 5.50 |
|  |  |  | 5 | Very high suitability | 5.95 |
|  |  |  | Total | 2-5 | 45.99 |
|  |  | 2081-2100 | 1 | Unsuitable | 90.79 |
|  |  |  | 2 | Low suitability | 29.79 |
|  |  |  | 3 | Medium suitability | 4.52 |
|  |  |  | 4 | High suitability | 5.58 |
|  |  |  | 5 | Very high suitability | 6.13 |
|  |  |  | Total | 2-5 | 46.02 |
| *Hordeum marinum* Huds. | current | current | 1 | Unsuitable | 107.74 |
|  |  |  | 2 | Low suitability | 13.56 |
|  |  |  | 3 | Medium suitability | 5.94 |
|  |  |  | 4 | High suitability | 5.31 |
|  |  |  | 5 | Very high suitability | 4.25 |
|  |  |  | Total | 2-5 | 29.06 |
|  | ssp126 | 2041-2060 | 1 | Unsuitable | 133.40 |
|  |  |  | 2 | Low suitability | 2.96 |
|  |  |  | 3 | Medium suitability | 0.41 |
|  |  |  | 4 | High suitability | 0.04 |
|  |  |  | 5 | Very high suitability | 0.00 |
|  |  |  | Total | 2-5 | 3.41 |
|  |  | 2061-2080 | 1 | Unsuitable | 133.29 |
|  |  |  | 2 | Low suitability | 3.05 |
|  |  |  | 3 | Medium suitability | 0.43 |
|  |  |  | 4 | High suitability | 0.04 |
|  |  |  | 5 | Very high suitability | 0.00 |
|  |  |  | Total | 2-5 | 3.51 |
|  |  | 2081-2100 | 1 | Unsuitable | 133.51 |
|  |  |  | 2 | Low suitability | 2.91 |
|  |  |  | 3 | Medium suitability | 0.37 |
|  |  |  | 4 | High suitability | 0.02 |
|  |  |  | 5 | Very high suitability | 0.00 |
|  |  |  | Total | 2-5 | 3.29 |
|  | ssp245 | 2041-2060 | 1 | Unsuitable | 133.30 |
|  |  |  | 2 | Low suitability | 3.06 |
|  |  |  | 3 | Medium suitability | 0.43 |
|  |  |  | 4 | High suitability | 0.02 |
|  |  |  | 5 | Very high suitability | 0.00 |
|  |  |  | Total | 2-5 | 3.50 |
|  |  | 2061-2080 | 1 | Unsuitable | 133.06 |
|  |  |  | 2 | Low suitability | 3.14 |
|  |  |  | 3 | Medium suitability | 0.56 |
|  |  |  | 4 | High suitability | 0.04 |
|  |  |  | 5 | Very high suitability | 0.00 |
|  |  |  | Total | 2-5 | 3.75 |
|  |  | 2081-2100 | 1 | Unsuitable | 133.09 |
|  |  |  | 2 | Low suitability | 3.06 |
|  |  |  | 3 | Medium suitability | 0.59 |
|  |  |  | 4 | High suitability | 0.06 |
|  |  |  | 5 | Very high suitability | 0.01 |
|  |  |  | Total | 2-5 | 3.71 |
|  | ssp370 | 2041-2060 | 1 | Unsuitable | 133.24 |
|  |  |  | 2 | Low suitability | 3.08 |
|  |  |  | 3 | Medium suitability | 0.45 |
|  |  |  | 4 | High suitability | 0.04 |
|  |  |  | 5 | Very high suitability | 0.00 |
|  |  |  | Total | 2-5 | 3.57 |
|  |  | 2061-2080 | 1 | Unsuitable | 133.03 |
|  |  |  | 2 | Low suitability | 3.18 |
|  |  |  | 3 | Medium suitability | 0.54 |
|  |  |  | 4 | High suitability | 0.05 |
|  |  |  | 5 | Very high suitability | 0.00 |
|  |  |  | Total | 2-5 | 3.78 |
|  |  | 2081-2100 | 1 | Unsuitable | 133.09 |
|  |  |  | 2 | Low suitability | 3.03 |
|  |  |  | 3 | Medium suitability | 0.63 |
|  |  |  | 4 | High suitability | 0.06 |
|  |  |  | 5 | Very high suitability | 0.01 |
|  |  |  | Total | 2-5 | 3.72 |
|  | ssp585 | 2041-2060 | 1 | Unsuitable | 133.27 |
|  |  |  | 2 | Low suitability | 3.04 |
|  |  |  | 3 | Medium suitability | 0.46 |
|  |  |  | 4 | High suitability | 0.04 |
|  |  |  | 5 | Very high suitability | 0.00 |
|  |  |  | Total | 2-5 | 3.54 |
|  |  | 2061-2080 | 1 | Unsuitable | 133.11 |
|  |  |  | 2 | Low suitability | 3.01 |
|  |  |  | 3 | Medium suitability | 0.63 |
|  |  |  | 4 | High suitability | 0.06 |
|  |  |  | 5 | Very high suitability | 0.00 |
|  |  |  | Total | 2-5 | 3.69 |
|  |  | 2081-2100 | 1 | Unsuitable | 132.74 |
|  |  |  | 2 | Low suitability | 3.29 |
|  |  |  | 3 | Medium suitability | 0.68 |
|  |  |  | 4 | High suitability | 0.09 |
|  |  |  | 5 | Very high suitability | 0.01 |
|  |  |  | Total | 2-5 | 4.06 |
| *Hordeum murinum* L. | current | current | 1 | Unsuitable | 82.26 |
|  |  |  | 2 | Low suitability | 21.19 |
|  |  |  | 3 | Medium suitability | 14.40 |
|  |  |  | 4 | High suitability | 10.07 |
|  |  |  | 5 | Very high suitability | 8.89 |
|  |  |  | Total | 2-5 | 54.54 |
|  | ssp126 | 2041-2060 | 1 | Unsuitable | 125.00 |
|  |  |  | 2 | Low suitability | 9.05 |
|  |  |  | 3 | Medium suitability | 1.62 |
|  |  |  | 4 | High suitability | 0.76 |
|  |  |  | 5 | Very high suitability | 0.37 |
|  |  |  | Total | 2-5 | 11.81 |
|  |  | 2061-2080 | 1 | Unsuitable | 124.91 |
|  |  |  | 2 | Low suitability | 9.13 |
|  |  |  | 3 | Medium suitability | 1.68 |
|  |  |  | 4 | High suitability | 0.77 |
|  |  |  | 5 | Very high suitability | 0.32 |
|  |  |  | Total | 2-5 | 11.90 |
|  |  | 2081-2100 | 1 | Unsuitable | 124.98 |
|  |  |  | 2 | Low suitability | 9.19 |
|  |  |  | 3 | Medium suitability | 1.62 |
|  |  |  | 4 | High suitability | 0.72 |
|  |  |  | 5 | Very high suitability | 0.30 |
|  |  |  | Total | 2-5 | 11.83 |
|  | ssp245 | 2041-2060 | 1 | Unsuitable | 124.83 |
|  |  |  | 2 | Low suitability | 9.16 |
|  |  |  | 3 | Medium suitability | 1.69 |
|  |  |  | 4 | High suitability | 0.77 |
|  |  |  | 5 | Very high suitability | 0.36 |
|  |  |  | Total | 2-5 | 11.98 |
|  |  | 2061-2080 | 1 | Unsuitable | 124.46 |
|  |  |  | 2 | Low suitability | 9.35 |
|  |  |  | 3 | Medium suitability | 1.68 |
|  |  |  | 4 | High suitability | 0.82 |
|  |  |  | 5 | Very high suitability | 0.50 |
|  |  |  | Total | 2-5 | 12.34 |
|  |  | 2081-2100 | 1 | Unsuitable | 124.33 |
|  |  |  | 2 | Low suitability | 9.46 |
|  |  |  | 3 | Medium suitability | 1.73 |
|  |  |  | 4 | High suitability | 0.80 |
|  |  |  | 5 | Very high suitability | 0.48 |
|  |  |  | Total | 2-5 | 12.48 |
|  | ssp370 | 2041-2060 | 1 | Unsuitable | 124.61 |
|  |  |  | 2 | Low suitability | 9.36 |
|  |  |  | 3 | Medium suitability | 1.68 |
|  |  |  | 4 | High suitability | 0.80 |
|  |  |  | 5 | Very high suitability | 0.35 |
|  |  |  | Total | 2-5 | 12.19 |
|  |  | 2061-2080 | 1 | Unsuitable | 123.81 |
|  |  |  | 2 | Low suitability | 9.85 |
|  |  |  | 3 | Medium suitability | 1.88 |
|  |  |  | 4 | High suitability | 0.82 |
|  |  |  | 5 | Very high suitability | 0.45 |
|  |  |  | Total | 2-5 | 13.00 |
|  |  | 2081-2100 | 1 | Unsuitable | 123.35 |
|  |  |  | 2 | Low suitability | 10.33 |
|  |  |  | 3 | Medium suitability | 1.86 |
|  |  |  | 4 | High suitability | 0.75 |
|  |  |  | 5 | Very high suitability | 0.52 |
|  |  |  | Total | 2-5 | 13.46 |
|  | ssp585 | 2041-2060 | 1 | Unsuitable | 124.57 |
|  |  |  | 2 | Low suitability | 9.42 |
|  |  |  | 3 | Medium suitability | 1.71 |
|  |  |  | 4 | High suitability | 0.75 |
|  |  |  | 5 | Very high suitability | 0.36 |
|  |  |  | Total | 2-5 | 12.24 |
|  |  | 2061-2080 | 1 | Unsuitable | 123.54 |
|  |  |  | 2 | Low suitability | 10.23 |
|  |  |  | 3 | Medium suitability | 1.71 |
|  |  |  | 4 | High suitability | 0.77 |
|  |  |  | 5 | Very high suitability | 0.56 |
|  |  |  | Total | 2-5 | 13.26 |
|  |  | 2081-2100 | 1 | Unsuitable | 122.83 |
|  |  |  | 2 | Low suitability | 10.52 |
|  |  |  | 3 | Medium suitability | 1.99 |
|  |  |  | 4 | High suitability | 0.84 |
|  |  |  | 5 | Very high suitability | 0.62 |
|  |  |  | Total | 2-5 | 13.98 |
| *Hordeum muticum* J.Presl | current | current | 1 | Unsuitable | 96.60 |
|  |  |  | 2 | Low suitability | 30.33 |
|  |  |  | 3 | Medium suitability | 6.21 |
|  |  |  | 4 | High suitability | 1.66 |
|  |  |  | 5 | Very high suitability | 2.01 |
|  |  |  | Total | 2-5 | 40.21 |
|  | ssp126 | 2041-2060 | 1 | Unsuitable | 111.81 |
|  |  |  | 2 | Low suitability | 7.15 |
|  |  |  | 3 | Medium suitability | 3.68 |
|  |  |  | 4 | High suitability | 11.66 |
|  |  |  | 5 | Very high suitability | 2.51 |
|  |  |  | Total | 2-5 | 25.00 |
|  |  | 2061-2080 | 1 | Unsuitable | 111.43 |
|  |  |  | 2 | Low suitability | 7.33 |
|  |  |  | 3 | Medium suitability | 3.71 |
|  |  |  | 4 | High suitability | 11.80 |
|  |  |  | 5 | Very high suitability | 2.52 |
|  |  |  | Total | 2-5 | 25.38 |
|  |  | 2081-2100 | 1 | Unsuitable | 111.33 |
|  |  |  | 2 | Low suitability | 7.19 |
|  |  |  | 3 | Medium suitability | 3.69 |
|  |  |  | 4 | High suitability | 12.06 |
|  |  |  | 5 | Very high suitability | 2.54 |
|  |  |  | Total | 2-5 | 25.48 |
|  | ssp245 | 2041-2060 | 1 | Unsuitable | 111.32 |
|  |  |  | 2 | Low suitability | 7.29 |
|  |  |  | 3 | Medium suitability | 3.72 |
|  |  |  | 4 | High suitability | 11.71 |
|  |  |  | 5 | Very high suitability | 2.76 |
|  |  |  | Total | 2-5 | 25.48 |
|  |  | 2061-2080 | 1 | Unsuitable | 111.62 |
|  |  |  | 2 | Low suitability | 7.00 |
|  |  |  | 3 | Medium suitability | 3.65 |
|  |  |  | 4 | High suitability | 11.94 |
|  |  |  | 5 | Very high suitability | 2.60 |
|  |  |  | Total | 2-5 | 25.19 |
|  |  | 2081-2100 | 1 | Unsuitable | 112.07 |
|  |  |  | 2 | Low suitability | 7.06 |
|  |  |  | 3 | Medium suitability | 3.44 |
|  |  |  | 4 | High suitability | 11.46 |
|  |  |  | 5 | Very high suitability | 2.78 |
|  |  |  | Total | 2-5 | 24.74 |
|  | ssp370 | 2041-2060 | 1 | Unsuitable | 111.58 |
|  |  |  | 2 | Low suitability | 7.29 |
|  |  |  | 3 | Medium suitability | 3.63 |
|  |  |  | 4 | High suitability | 11.83 |
|  |  |  | 5 | Very high suitability | 2.49 |
|  |  |  | Total | 2-5 | 25.23 |
|  |  | 2061-2080 | 1 | Unsuitable | 111.67 |
|  |  |  | 2 | Low suitability | 7.27 |
|  |  |  | 3 | Medium suitability | 3.60 |
|  |  |  | 4 | High suitability | 11.55 |
|  |  |  | 5 | Very high suitability | 2.71 |
|  |  |  | Total | 2-5 | 25.13 |
|  |  | 2081-2100 | 1 | Unsuitable | 111.33 |
|  |  |  | 2 | Low suitability | 7.23 |
|  |  |  | 3 | Medium suitability | 3.50 |
|  |  |  | 4 | High suitability | 11.78 |
|  |  |  | 5 | Very high suitability | 2.96 |
|  |  |  | Total | 2-5 | 25.47 |
|  | ssp585 | 2041-2060 | 1 | Unsuitable | 111.04 |
|  |  |  | 2 | Low suitability | 7.15 |
|  |  |  | 3 | Medium suitability | 3.78 |
|  |  |  | 4 | High suitability | 12.08 |
|  |  |  | 5 | Very high suitability | 2.76 |
|  |  |  | Total | 2-5 | 25.77 |
|  |  | 2061-2080 | 1 | Unsuitable | 111.25 |
|  |  |  | 2 | Low suitability | 6.93 |
|  |  |  | 3 | Medium suitability | 3.71 |
|  |  |  | 4 | High suitability | 12.07 |
|  |  |  | 5 | Very high suitability | 2.84 |
|  |  |  | Total | 2-5 | 25.55 |
|  |  | 2081-2100 | 1 | Unsuitable | 111.07 |
|  |  |  | 2 | Low suitability | 6.83 |
|  |  |  | 3 | Medium suitability | 3.65 |
|  |  |  | 4 | High suitability | 12.35 |
|  |  |  | 5 | Very high suitability | 2.90 |
|  |  |  | Total | 2-5 | 25.74 |
| *Hordeum pusillum* Nutt. | current | current | 1 | Unsuitable | 103.36 |
|  |  |  | 2 | Low suitability | 18.47 |
|  |  |  | 3 | Medium suitability | 7.22 |
|  |  |  | 4 | High suitability | 3.45 |
|  |  |  | 5 | Very high suitability | 4.31 |
|  |  |  | Total | 2-5 | 33.44 |
|  | ssp126 | 2041-2060 | 1 | Unsuitable | 133.29 |
|  |  |  | 2 | Low suitability | 2.38 |
|  |  |  | 3 | Medium suitability | 0.81 |
|  |  |  | 4 | High suitability | 0.32 |
|  |  |  | 5 | Very high suitability | 0.01 |
|  |  |  | Total | 2-5 | 3.51 |
|  |  | 2061-2080 | 1 | Unsuitable | 133.22 |
|  |  |  | 2 | Low suitability | 2.38 |
|  |  |  | 3 | Medium suitability | 0.85 |
|  |  |  | 4 | High suitability | 0.34 |
|  |  |  | 5 | Very high suitability | 0.01 |
|  |  |  | Total | 2-5 | 3.58 |
|  |  | 2081-2100 | 1 | Unsuitable | 133.33 |
|  |  |  | 2 | Low suitability | 2.39 |
|  |  |  | 3 | Medium suitability | 0.81 |
|  |  |  | 4 | High suitability | 0.27 |
|  |  |  | 5 | Very high suitability | 0.01 |
|  |  |  | Total | 2-5 | 3.47 |
|  | ssp245 | 2041-2060 | 1 | Unsuitable | 133.30 |
|  |  |  | 2 | Low suitability | 2.29 |
|  |  |  | 3 | Medium suitability | 0.82 |
|  |  |  | 4 | High suitability | 0.38 |
|  |  |  | 5 | Very high suitability | 0.02 |
|  |  |  | Total | 2-5 | 3.51 |
|  |  | 2061-2080 | 1 | Unsuitable | 133.15 |
|  |  |  | 2 | Low suitability | 2.29 |
|  |  |  | 3 | Medium suitability | 0.84 |
|  |  |  | 4 | High suitability | 0.49 |
|  |  |  | 5 | Very high suitability | 0.04 |
|  |  |  | Total | 2-5 | 3.65 |
|  |  | 2081-2100 | 1 | Unsuitable | 133.17 |
|  |  |  | 2 | Low suitability | 2.26 |
|  |  |  | 3 | Medium suitability | 0.88 |
|  |  |  | 4 | High suitability | 0.45 |
|  |  |  | 5 | Very high suitability | 0.04 |
|  |  |  | Total | 2-5 | 3.63 |
|  | ssp370 | 2041-2060 | 1 | Unsuitable | 133.20 |
|  |  |  | 2 | Low suitability | 2.32 |
|  |  |  | 3 | Medium suitability | 0.86 |
|  |  |  | 4 | High suitability | 0.40 |
|  |  |  | 5 | Very high suitability | 0.02 |
|  |  |  | Total | 2-5 | 3.60 |
|  |  | 2061-2080 | 1 | Unsuitable | 133.02 |
|  |  |  | 2 | Low suitability | 2.30 |
|  |  |  | 3 | Medium suitability | 0.91 |
|  |  |  | 4 | High suitability | 0.51 |
|  |  |  | 5 | Very high suitability | 0.07 |
|  |  |  | Total | 2-5 | 3.78 |
|  |  | 2081-2100 | 1 | Unsuitable | 133.13 |
|  |  |  | 2 | Low suitability | 2.09 |
|  |  |  | 3 | Medium suitability | 0.92 |
|  |  |  | 4 | High suitability | 0.52 |
|  |  |  | 5 | Very high suitability | 0.14 |
|  |  |  | Total | 2-5 | 3.67 |
|  | ssp585 | 2041-2060 | 1 | Unsuitable | 133.23 |
|  |  |  | 2 | Low suitability | 2.26 |
|  |  |  | 3 | Medium suitability | 0.87 |
|  |  |  | 4 | High suitability | 0.43 |
|  |  |  | 5 | Very high suitability | 0.02 |
|  |  | 2041-2060 | Total | 2-5 | 3.58 |
|  |  |  | 1 | Unsuitable | 133.03 |
|  |  |  | 2 | Low suitability | 2.21 |
|  |  |  | 3 | Medium suitability | 0.96 |
|  |  |  | 4 | High suitability | 0.52 |
|  |  |  | 5 | Very high suitability | 0.09 |
|  |  |  | Total | 2-5 | 3.78 |
|  |  | 2081-2100 | 1 | Unsuitable | 132.80 |
|  |  |  | 2 | Low suitability | 2.18 |
|  |  |  | 3 | Medium suitability | 0.97 |
|  |  |  | 4 | High suitability | 0.65 |
|  |  |  | 5 | Very high suitability | 0.21 |
|  |  |  | Total | 2-5 | 4.01 |
| *Hordeum roshevitzii* Bowden | current | current | 1 | Unsuitable | 97.51 |
|  |  |  | 2 | Low suitability | 18.02 |
|  |  |  | 3 | Medium suitability | 9.87 |
|  |  |  | 4 | High suitability | 4.89 |
|  |  |  | 5 | Very high suitability | 6.52 |
|  |  |  | Total | 2-5 | 39.30 |
|  | ssp126 | 2041-2060 | 1 | Unsuitable | 129.40 |
|  |  |  | 2 | Low suitability | 3.98 |
|  |  |  | 3 | Medium suitability | 0.94 |
|  |  |  | 4 | High suitability | 0.69 |
|  |  |  | 5 | Very high suitability | 1.79 |
|  |  |  | Total | 2-5 | 7.41 |
|  |  | 2061-2080 | 1 | Unsuitable | 129.51 |
|  |  |  | 2 | Low suitability | 3.90 |
|  |  |  | 3 | Medium suitability | 1.06 |
|  |  |  | 4 | High suitability | 0.58 |
|  |  |  | 5 | Very high suitability | 1.75 |
|  |  |  | Total | 2-5 | 7.29 |
|  |  | 2081-2100 | 1 | Unsuitable | 129.54 |
|  |  |  | 2 | Low suitability | 3.95 |
|  |  |  | 3 | Medium suitability | 1.00 |
|  |  |  | 4 | High suitability | 0.56 |
|  |  |  | 5 | Very high suitability | 1.77 |
|  |  |  | Total | 2-5 | 7.27 |
|  | ssp245 | 2041-2060 | 1 | Unsuitable | 129.69 |
|  |  |  | 2 | Low suitability | 3.87 |
|  |  |  | 3 | Medium suitability | 0.95 |
|  |  |  | 4 | High suitability | 0.58 |
|  |  |  | 5 | Very high suitability | 1.71 |
|  |  |  | Total | 2-5 | 7.11 |
|  |  | 2061-2080 | 1 | Unsuitable | 129.90 |
|  |  |  | 2 | Low suitability | 3.71 |
|  |  |  | 3 | Medium suitability | 0.87 |
|  |  |  | 4 | High suitability | 0.57 |
|  |  |  | 5 | Very high suitability | 1.76 |
|  |  |  | Total | 2-5 | 6.90 |
|  |  | 2081-2100 | 1 | Unsuitable | 130.13 |
|  |  |  | 2 | Low suitability | 3.50 |
|  |  |  | 3 | Medium suitability | 0.88 |
|  |  |  | 4 | High suitability | 0.54 |
|  |  |  | 5 | Very high suitability | 1.76 |
|  |  |  | Total | 2-5 | 6.68 |
|  | ssp370 | 2041-2060 | 1 | Unsuitable | 129.68 |
|  |  |  | 2 | Low suitability | 3.86 |
|  |  |  | 3 | Medium suitability | 0.94 |
|  |  |  | 4 | High suitability | 0.52 |
|  |  |  | 5 | Very high suitability | 1.80 |
|  |  |  | Total | 2-5 | 7.13 |
|  |  | 2061-2080 | 1 | Unsuitable | 130.18 |
|  |  |  | 2 | Low suitability | 3.46 |
|  |  |  | 3 | Medium suitability | 0.88 |
|  |  |  | 4 | High suitability | 0.54 |
|  |  |  | 5 | Very high suitability | 1.74 |
|  |  |  | Total | 2-5 | 6.62 |
|  |  | 2081-2100 | 1 | Unsuitable | 130.38 |
|  |  |  | 2 | Low suitability | 3.51 |
|  |  |  | 3 | Medium suitability | 0.74 |
|  |  |  | 4 | High suitability | 0.53 |
|  |  |  | 5 | Very high suitability | 1.64 |
|  |  |  | Total | 2-5 | 6.42 |
|  | ssp585 | 2041-2060 | 1 | Unsuitable | 129.86 |
|  |  |  | 2 | Low suitability | 3.84 |
|  |  |  | 3 | Medium suitability | 0.89 |
|  |  |  | 4 | High suitability | 0.52 |
|  |  |  | 5 | Very high suitability | 1.69 |
|  |  |  | Total | 2-5 | 6.95 |
|  |  | 2061-2080 | 1 | Unsuitable | 130.18 |
|  |  |  | 2 | Low suitability | 3.57 |
|  |  |  | 3 | Medium suitability | 0.91 |
|  |  |  | 4 | High suitability | 0.54 |
|  |  |  | 5 | Very high suitability | 1.61 |
|  |  |  | Total | 2-5 | 6.63 |
|  |  | 2081-2100 | 1 | Unsuitable | 130.24 |
|  |  |  | 2 | Low suitability | 3.56 |
|  |  |  | 3 | Medium suitability | 0.80 |
|  |  |  | 4 | High suitability | 0.57 |
|  |  |  | 5 | Very high suitability | 1.64 |
|  |  |  | Total | 2-5 | 6.56 |
| *Hordeum spontaneum* K. Koch | current | current | 1 | Unsuitable | 97.90 |
|  |  |  | 2 | Low suitability | 25.03 |
|  |  |  | 3 | Medium suitability | 7.00 |
|  |  |  | 4 | High suitability | 2.40 |
|  |  |  | 5 | Very high suitability | 4.47 |
|  |  |  | Total | 2-5 | 38.90 |
|  | ssp126 | 2041-2060 | 1 | Unsuitable | 135.96 |
|  |  |  | 2 | Low suitability | 0.81 |
|  |  |  | 3 | Medium suitability | 0.04 |
|  |  |  | 4 | High suitability | 0.00 |
|  |  |  | 5 | Very high suitability | 0.00 |
|  |  |  | Total | 2-5 | 0.85 |
|  |  | 2061-2080 | 1 | Unsuitable | 135.95 |
|  |  |  | 2 | Low suitability | 0.82 |
|  |  |  | 3 | Medium suitability | 0.04 |
|  |  |  | 4 | High suitability | 0.00 |
|  |  |  | 5 | Very high suitability | 0.00 |
|  |  |  | Total | 2-5 | 0.86 |
|  |  | 2081-2100 | 1 | Unsuitable | 136.03 |
|  |  |  | 2 | Low suitability | 0.70 |
|  |  |  | 3 | Medium suitability | 0.08 |
|  |  |  | 4 | High suitability | 0.00 |
|  |  |  | 5 | Very high suitability | 0.00 |
|  |  |  | Total | 2-5 | 0.78 |
|  | ssp245 | 2041-2060 | 1 | Unsuitable | 136.03 |
|  |  |  | 2 | Low suitability | 0.67 |
|  |  |  | 3 | Medium suitability | 0.11 |
|  |  |  | 4 | High suitability | 0.00 |
|  |  |  | 5 | Very high suitability | 0.00 |
|  |  |  | Total | 2-5 | 0.78 |
|  |  | 2061-2080 | 1 | Unsuitable | 135.84 |
|  |  |  | 2 | Low suitability | 0.90 |
|  |  |  | 3 | Medium suitability | 0.08 |
|  |  |  | 4 | High suitability | 0.00 |
|  |  |  | 5 | Very high suitability | 0.00 |
|  |  |  | Total | 2-5 | 0.97 |
|  |  | 2081-2100 | 1 | Unsuitable | 135.93 |
|  |  |  | 2 | Low suitability | 0.81 |
|  |  |  | 3 | Medium suitability | 0.06 |
|  |  |  | 4 | High suitability | 0.00 |
|  |  |  | 5 | Very high suitability | 0.00 |
|  |  |  | Total | 2-5 | 0.87 |
|  | ssp370 | 2041-2060 | 1 | Unsuitable | 136.02 |
|  |  |  | 2 | Low suitability | 0.73 |
|  |  |  | 3 | Medium suitability | 0.06 |
|  |  |  | 4 | High suitability | 0.00 |
|  |  |  | 5 | Very high suitability | 0.00 |
|  |  |  | Total | 2-5 | 0.79 |
|  |  | 2061-2080 | 1 | Unsuitable | 135.91 |
|  |  |  | 2 | Low suitability | 0.81 |
|  |  |  | 3 | Medium suitability | 0.08 |
|  |  |  | 4 | High suitability | 0.00 |
|  |  |  | 5 | Very high suitability | 0.00 |
|  |  |  | Total | 2-5 | 0.89 |
|  |  | 2081-2100 | 1 | Unsuitable | 136.00 |
|  |  |  | 2 | Low suitability | 0.77 |
|  |  |  | 3 | Medium suitability | 0.03 |
|  |  |  | 4 | High suitability | 0.00 |
|  |  |  | 5 | Very high suitability | 0.00 |
|  |  |  | Total | 2-5 | 0.80 |
|  | ssp585 | 2041-2060 | 1 | Unsuitable | 136.03 |
|  |  |  | 2 | Low suitability | 0.71 |
|  |  |  | 3 | Medium suitability | 0.07 |
|  |  |  | 4 | High suitability | 0.00 |
|  |  |  | 5 | Very high suitability | 0.00 |
|  |  |  | Total | 2-5 | 0.78 |
|  |  | 2061-2080 | 1 | Unsuitable | 135.95 |
|  |  |  | 2 | Low suitability | 0.75 |
|  |  |  | 3 | Medium suitability | 0.11 |
|  |  |  | 4 | High suitability | 0.00 |
|  |  |  | 5 | Very high suitability | 0.00 |
|  |  |  | Total | 2-5 | 0.85 |
|  |  | 2081-2100 | 1 | Unsuitable | 135.89 |
|  |  |  | 2 | Low suitability | 0.87 |
|  |  |  | 3 | Medium suitability | 0.05 |
|  |  |  | 4 | High suitability | 0.00 |
|  |  |  | 5 | Very high suitability | 0.00 |
|  |  |  | Total | 2-5 | 0.92 |
| *Hordeum stenostachys* Godr. | current | current | 1 | Unsuitable | 103.99 |
|  |  |  | 2 | Low suitability | 18.24 |
|  |  |  | 3 | Medium suitability | 6.51 |
|  |  |  | 4 | High suitability | 3.66 |
|  |  |  | 5 | Very high suitability | 4.41 |
|  |  |  | Total | 2-5 | 32.82 |
|  | ssp126 | 2041-2060 | 1 | Unsuitable | 134.54 |
|  |  |  | 2 | Low suitability | 1.32 |
|  |  |  | 3 | Medium suitability | 0.35 |
|  |  |  | 4 | High suitability | 0.21 |
|  |  |  | 5 | Very high suitability | 0.39 |
|  |  |  | Total | 2-5 | 2.27 |
|  |  | 2061-2080 | 1 | Unsuitable | 134.47 |
|  |  |  | 2 | Low suitability | 1.37 |
|  |  |  | 3 | Medium suitability | 0.40 |
|  |  |  | 4 | High suitability | 0.24 |
|  |  |  | 5 | Very high suitability | 0.32 |
|  |  |  | Total | 2-5 | 2.33 |
|  |  | 2081-2100 | 1 | Unsuitable | 134.50 |
|  |  |  | 2 | Low suitability | 1.32 |
|  |  |  | 3 | Medium suitability | 0.40 |
|  |  |  | 4 | High suitability | 0.26 |
|  |  |  | 5 | Very high suitability | 0.32 |
|  |  |  | Total | 2-5 | 2.30 |
|  | ssp245 | 2041-2060 | 1 | Unsuitable | 134.42 |
|  |  |  | 2 | Low suitability | 1.32 |
|  |  |  | 3 | Medium suitability | 0.46 |
|  |  |  | 4 | High suitability | 0.23 |
|  |  |  | 5 | Very high suitability | 0.37 |
|  |  |  | Total | 2-5 | 2.38 |
|  |  | 2061-2080 | 1 | Unsuitable | 134.42 |
|  |  |  | 2 | Low suitability | 1.32 |
|  |  |  | 3 | Medium suitability | 0.47 |
|  |  |  | 4 | High suitability | 0.24 |
|  |  |  | 5 | Very high suitability | 0.37 |
|  |  |  | Total | 2-5 | 2.39 |
|  |  | 2081-2100 | 1 | Unsuitable | 134.44 |
|  |  |  | 2 | Low suitability | 1.27 |
|  |  |  | 3 | Medium suitability | 0.49 |
|  |  |  | 4 | High suitability | 0.26 |
|  |  |  | 5 | Very high suitability | 0.35 |
|  |  |  | Total | 2-5 | 2.37 |
|  | ssp370 | 2041-2060 | 1 | Unsuitable | 134.53 |
|  |  |  | 2 | Low suitability | 1.27 |
|  |  |  | 3 | Medium suitability | 0.40 |
|  |  |  | 4 | High suitability | 0.24 |
|  |  |  | 5 | Very high suitability | 0.37 |
|  |  |  | Total | 2-5 | 2.28 |
|  |  | 2061-2080 | 1 | Unsuitable | 134.39 |
|  |  |  | 2 | Low suitability | 1.27 |
|  |  |  | 3 | Medium suitability | 0.49 |
|  |  |  | 4 | High suitability | 0.28 |
|  |  |  | 5 | Very high suitability | 0.38 |
|  |  |  | Total | 2-5 | 2.41 |
|  |  | 2081-2100 | 1 | Unsuitable | 134.37 |
|  |  |  | 2 | Low suitability | 1.26 |
|  |  |  | 3 | Medium suitability | 0.53 |
|  |  |  | 4 | High suitability | 0.24 |
|  |  |  | 5 | Very high suitability | 0.40 |
|  |  |  | Total | 2-5 | 2.43 |
|  | ssp585 | 2041-2060 | 1 | Unsuitable | 134.43 |
|  |  |  | 2 | Low suitability | 1.31 |
|  |  |  | 3 | Medium suitability | 0.44 |
|  |  |  | 4 | High suitability | 0.23 |
|  |  |  | 5 | Very high suitability | 0.40 |
|  |  |  | Total | 2-5 | 2.38 |
|  |  | 2061-2080 | 1 | Unsuitable | 134.26 |
|  |  |  | 2 | Low suitability | 1.27 |
|  |  |  | 3 | Medium suitability | 0.54 |
|  |  |  | 4 | High suitability | 0.26 |
|  |  |  | 5 | Very high suitability | 0.48 |
|  |  |  | Total | 2-5 | 2.55 |
|  |  | 2081-2100 | 1 | Unsuitable | 134.26 |
|  |  |  | 2 | Low suitability | 1.34 |
|  |  |  | 3 | Medium suitability | 0.49 |
|  |  |  | 4 | High suitability | 0.25 |
|  |  |  | 5 | Very high suitability | 0.46 |
|  |  |  | Total | 2-5 | 2.54 |

**Supplementary Table 6.** Calculation results of niche breadth for 13 *Hordeum* species.

| Species | Scenario | Period | Niche Width | Baseline Width | Change Rate | Response Type |
| --- | --- | --- | --- | --- | --- | --- |
| *Hordeum agriocrithon* A. E. Åberg | current | current | 0.696 | 0.696 | 0 | Stable |
| *Hordeum bogdanii* Wilensky |  |  | 0.679 | 0.679 | 0 | Stable |
| *Hordeum brevisubulatum* (Trin.) Link |  |  | 0.648 | 0.648 | 0 | Stable |
| *Hordeum bulbosum* L. |  |  | 0.688 | 0.688 | 0 | Stable |
| *Hordeum californicum* Covas & Stebbins |  |  | 0.754 | 0.754 | 0 | Stable |
| *Hordeum chilense* Roem. & Schult. |  |  | 0.770 | 0.770 | 0 | Stable |
| *Hordeum marinum* Huds. |  |  | 0.681 | 0.681 | 0 | Stable |
| *Hordeum murinum* L. |  |  | 0.707 | 0.707 | 0 | Stable |
| *Hordeum muticum* J.Presl |  |  | 0.818 | 0.818 | 0 | Stable |
| *Hordeum pusillum* Nutt. |  |  | 0.692 | 0.692 | 0 | Stable |
| *Hordeum roshevitzii* Bowden |  |  | 0.657 | 0.657 | 0 | Stable |
| *Hordeum spontaneum* K. Koch |  |  | 0.737 | 0.737 | 0 | Stable |
| *Hordeum stenostachys* Godr. |  |  | 0.739 | 0.739 | 0 | Stable |
| *Hordeum agriocrithon* A. E. Åberg | ssp126 | 2041-2060 | 0.739 | 0.696 | 6.26 | Expansion |
|  |  | 2061-2080 | 0.739 | 0.696 | 6.25 | Expansion |
|  |  | 2081-2100 | 0.739 | 0.696 | 6.25 | Expansion |
|  | ssp245 | 2041-2060 | 0.739 | 0.696 | 6.19 | Expansion |
|  |  | 2061-2080 | 0.738 | 0.696 | 6.15 | Expansion |
|  |  | 2081-2100 | 0.738 | 0.696 | 6.1 | Expansion |
|  | ssp370 | 2041-2060 | 0.739 | 0.696 | 6.18 | Expansion |
|  |  | 2061-2080 | 0.737 | 0.696 | 6.02 | Expansion |
|  |  | 2081-2100 | 0.737 | 0.696 | 5.91 | Expansion |
|  | ssp585 | 2041-2060 | 0.738 | 0.696 | 6.16 | Expansion |
|  |  | 2061-2080 | 0.737 | 0.696 | 5.98 | Expansion |
|  |  | 2081-2100 | 0.736 | 0.696 | 5.82 | Expansion |
| *Hordeum bogdanii* Wilensky | ssp126 | 2041-2060 | 0.565 | 0.679 | -16.79 | Contraction |
|  |  | 2061-2080 | 0.566 | 0.679 | -16.75 | Contraction |
|  |  | 2081-2100 | 0.562 | 0.679 | -17.29 | Contraction |
|  | ssp245 | 2041-2060 | 0.564 | 0.679 | -16.95 | Contraction |
|  |  | 2061-2080 | 0.563 | 0.679 | -17.15 | Contraction |
|  |  | 2081-2100 | 0.562 | 0.679 | -17.25 | Contraction |
|  | ssp370 | 2041-2060 | 0.563 | 0.679 | -17.08 | Contraction |
|  |  | 2061-2080 | 0.563 | 0.679 | -17.08 | Contraction |
|  |  | 2081-2100 | 0.560 | 0.679 | -17.5 | Contraction |
|  | ssp585 | 2041-2060 | 0.562 | 0.679 | -17.34 | Contraction |
|  |  | 2061-2080 | 0.560 | 0.679 | -17.61 | Contraction |
|  |  | 2081-2100 | 0.563 | 0.679 | -17.08 | Contraction |
| *Hordeum brevisubulatum* (Trin.) Link | ssp126 | 2041-2060 | 0.535 | 0.648 | -17.51 | Contraction |
|  |  | 2061-2080 | 0.535 | 0.648 | -17.52 | Contraction |
|  |  | 2081-2100 | 0.531 | 0.648 | -18.1 | Contraction |
|  | ssp245 | 2041-2060 | 0.533 | 0.648 | -17.78 | Contraction |
|  |  | 2061-2080 | 0.531 | 0.648 | -18.12 | Contraction |
|  |  | 2081-2100 | 0.532 | 0.648 | -17.92 | Contraction |
|  | ssp370 | 2041-2060 | 0.534 | 0.648 | -17.66 | Contraction |
|  |  | 2061-2080 | 0.533 | 0.648 | -17.73 | Contraction |
|  |  | 2081-2100 | 0.530 | 0.648 | -18.18 | Contraction |
|  | ssp585 | 2041-2060 | 0.534 | 0.648 | -17.62 | Contraction |
|  |  | 2061-2080 | 0.532 | 0.648 | -18.02 | Contraction |
|  |  | 2081-2100 | 0.534 | 0.648 | -17.62 | Contraction |
| *Hordeum bulbosum* L. | ssp126 | 2041-2060 | 0.527 | 0.688 | -23.38 | Contraction |
|  |  | 2061-2080 | 0.528 | 0.688 | -23.35 | Contraction |
|  |  | 2081-2100 | 0.525 | 0.688 | -23.69 | Contraction |
|  | ssp245 | 2041-2060 | 0.529 | 0.688 | -23.12 | Contraction |
|  |  | 2061-2080 | 0.524 | 0.688 | -23.83 | Contraction |
|  |  | 2081-2100 | 0.526 | 0.688 | -23.64 | Contraction |
|  | ssp370 | 2041-2060 | 0.527 | 0.688 | -23.38 | Contraction |
|  |  | 2061-2080 | 0.525 | 0.688 | -23.79 | Contraction |
|  |  | 2081-2100 | 0.522 | 0.688 | -24.12 | Contraction |
|  | ssp585 | 2041-2060 | 0.524 | 0.688 | -23.86 | Contraction |
|  |  | 2061-2080 | 0.526 | 0.688 | -23.5 | Contraction |
|  |  | 2081-2100 | 0.525 | 0.688 | -23.68 | Contraction |
| *Hordeum californicum* Covas & Stebbins | ssp126 | 2041-2060 | 0.569 | 0.754 | -24.59 | Contraction |
|  |  | 2061-2080 | 0.569 | 0.754 | -24.56 | Contraction |
|  |  | 2081-2100 | 0.569 | 0.754 | -24.6 | Contraction |
|  | ssp245 | 2041-2060 | 0.571 | 0.754 | -24.24 | Contraction |
|  |  | 2061-2080 | 0.571 | 0.754 | -24.24 | Contraction |
|  |  | 2081-2100 | 0.572 | 0.754 | -24.22 | Contraction |
|  | ssp370 | 2041-2060 | 0.570 | 0.754 | -24.4 | Contraction |
|  |  | 2061-2080 | 0.570 | 0.754 | -24.39 | Contraction |
|  |  | 2081-2100 | 0.573 | 0.754 | -24.03 | Contraction |
|  | ssp585 | 2041-2060 | 0.571 | 0.754 | -24.34 | Contraction |
|  |  | 2061-2080 | 0.573 | 0.754 | -24.05 | Contraction |
|  |  | 2081-2100 | 0.573 | 0.754 | -24.04 | Contraction |
| *Hordeum chilense* Roem. & Schult. | ssp126 | 2041-2060 | 0.711 | 0.770 | -7.68 | Contraction |
|  |  | 2061-2080 | 0.712 | 0.770 | -7.57 | Contraction |
|  |  | 2081-2100 | 0.712 | 0.770 | -7.57 | Contraction |
|  | ssp245 | 2041-2060 | 0.712 | 0.770 | -7.6 | Contraction |
|  |  | 2061-2080 | 0.713 | 0.770 | -7.52 | Contraction |
|  |  | 2081-2100 | 0.712 | 0.770 | -7.6 | Contraction |
|  | ssp370 | 2041-2060 | 0.712 | 0.770 | -7.54 | Contraction |
|  |  | 2061-2080 | 0.712 | 0.770 | -7.6 | Contraction |
|  |  | 2081-2100 | 0.713 | 0.770 | -7.48 | Contraction |
|  | ssp585 | 2041-2060 | 0.712 | 0.770 | -7.54 | Contraction |
|  |  | 2061-2080 | 0.713 | 0.770 | -7.45 | Contraction |
|  |  | 2081-2100 | 0.713 | 0.770 | -7.52 | Contraction |
| *Hordeum marinum* Huds. | ssp126 | 2041-2060 | 0.532 | 0.681 | -21.89 | Contraction |
|  |  | 2061-2080 | 0.532 | 0.681 | -21.91 | Contraction |
|  |  | 2081-2100 | 0.535 | 0.681 | -21.44 | Contraction |
|  | ssp245 | 2041-2060 | 0.535 | 0.681 | -21.53 | Contraction |
|  |  | 2061-2080 | 0.531 | 0.681 | -22.04 | Contraction |
|  |  | 2081-2100 | 0.532 | 0.681 | -21.9 | Contraction |
|  | ssp370 | 2041-2060 | 0.535 | 0.681 | -21.45 | Contraction |
|  |  | 2061-2080 | 0.535 | 0.681 | -21.45 | Contraction |
|  |  | 2081-2100 | 0.533 | 0.681 | -21.82 | Contraction |
|  | ssp585 | 2041-2060 | 0.533 | 0.681 | -21.71 | Contraction |
|  |  | 2061-2080 | 0.535 | 0.681 | -21.47 | Contraction |
|  |  | 2081-2100 | 0.535 | 0.681 | -21.5 | Contraction |
| *Hordeum murinum* L. | ssp126 | 2041-2060 | 0.557 | 0.707 | -21.21 | Contraction |
|  |  | 2061-2080 | 0.558 | 0.707 | -21.07 | Contraction |
|  |  | 2081-2100 | 0.559 | 0.707 | -20.96 | Contraction |
|  | ssp245 | 2041-2060 | 0.560 | 0.707 | -20.85 | Contraction |
|  |  | 2061-2080 | 0.558 | 0.707 | -21.16 | Contraction |
|  |  | 2081-2100 | 0.559 | 0.707 | -20.96 | Contraction |
|  | ssp370 | 2041-2060 | 0.559 | 0.707 | -20.9 | Contraction |
|  |  | 2061-2080 | 0.560 | 0.707 | -20.75 | Contraction |
|  |  | 2081-2100 | 0.561 | 0.707 | -20.65 | Contraction |
|  | ssp585 | 2041-2060 | 0.558 | 0.707 | -21.1 | Contraction |
|  |  | 2061-2080 | 0.561 | 0.707 | -20.65 | Contraction |
|  |  | 2081-2100 | 0.563 | 0.707 | -20.47 | Contraction |
| *Hordeum muticum* J.Presl | ssp126 | 2041-2060 | 0.648 | 0.818 | -20.87 | Contraction |
|  |  | 2061-2080 | 0.648 | 0.818 | -20.81 | Contraction |
|  |  | 2081-2100 | 0.649 | 0.818 | -20.7 | Contraction |
|  | ssp245 | 2041-2060 | 0.646 | 0.818 | -21.03 | Contraction |
|  |  | 2061-2080 | 0.648 | 0.818 | -20.84 | Contraction |
|  |  | 2081-2100 | 0.646 | 0.818 | -21.09 | Contraction |
|  | ssp370 | 2041-2060 | 0.649 | 0.818 | -20.72 | Contraction |
|  |  | 2061-2080 | 0.647 | 0.818 | -20.9 | Contraction |
|  |  | 2081-2100 | 0.648 | 0.818 | -20.86 | Contraction |
|  | ssp585 | 2041-2060 | 0.649 | 0.818 | -20.65 | Contraction |
|  |  | 2061-2080 | 0.651 | 0.818 | -20.39 | Contraction |
|  |  | 2081-2100 | 0.650 | 0.818 | -20.62 | Contraction |
| *Hordeum pusillum* Nutt. | ssp126 | 2041-2060 | 0.552 | 0.692 | -20.21 | Contraction |
|  |  | 2061-2080 | 0.550 | 0.692 | -20.42 | Contraction |
|  |  | 2081-2100 | 0.552 | 0.692 | -20.21 | Contraction |
|  | ssp245 | 2041-2060 | 0.549 | 0.692 | -20.66 | Contraction |
|  |  | 2061-2080 | 0.544 | 0.692 | -21.41 | Contraction |
|  |  | 2081-2100 | 0.547 | 0.692 | -20.95 | Contraction |
|  | ssp370 | 2041-2060 | 0.550 | 0.692 | -20.48 | Contraction |
|  |  | 2061-2080 | 0.546 | 0.692 | -21.03 | Contraction |
|  |  | 2081-2100 | 0.540 | 0.692 | -21.91 | Contraction |
|  | ssp585 | 2041-2060 | 0.544 | 0.692 | -21.29 | Contraction |
|  |  | 2061-2080 | 0.540 | 0.692 | -21.97 | Contraction |
|  |  | 2081-2100 | 0.539 | 0.692 | -22.06 | Contraction |
| *Hordeum roshevitzii* Bowden | ssp126 | 2041-2060 | 0.589 | 0.657 | -10.39 | Contraction |
|  |  | 2061-2080 | 0.590 | 0.657 | -10.26 | Contraction |
|  |  | 2081-2100 | 0.588 | 0.657 | -10.6 | Contraction |
|  | ssp245 | 2041-2060 | 0.588 | 0.657 | -10.48 | Contraction |
|  |  | 2061-2080 | 0.587 | 0.657 | -10.69 | Contraction |
|  |  | 2081-2100 | 0.583 | 0.657 | -11.29 | Contraction |
|  | ssp370 | 2041-2060 | 0.589 | 0.657 | -10.41 | Contraction |
|  |  | 2061-2080 | 0.587 | 0.657 | -10.66 | Contraction |
|  |  | 2081-2100 | 0.587 | 0.657 | -10.68 | Contraction |
|  | ssp585 | 2041-2060 | 0.587 | 0.657 | -10.64 | Contraction |
|  |  | 2061-2080 | 0.583 | 0.657 | -11.36 | Contraction |
|  |  | 2081-2100 | 0.586 | 0.657 | -10.83 | Contraction |
| *Hordeum spontaneum* K. Koch | ssp126 | 2041-2060 | 0.554 | 0.737 | -24.8 | Contraction |
|  |  | 2061-2080 | 0.556 | 0.737 | -24.58 | Contraction |
|  |  | 2081-2100 | 0.555 | 0.737 | -24.73 | Contraction |
|  | ssp245 | 2041-2060 | 0.558 | 0.737 | -24.29 | Contraction |
|  |  | 2061-2080 | 0.552 | 0.737 | -25.14 | Contraction |
|  |  | 2081-2100 | 0.554 | 0.737 | -24.75 | Contraction |
|  | ssp370 | 2041-2060 | 0.557 | 0.737 | -24.46 | Contraction |
|  |  | 2061-2080 | 0.555 | 0.737 | -24.65 | Contraction |
|  |  | 2081-2100 | 0.554 | 0.737 | -24.75 | Contraction |
|  | ssp585 | 2041-2060 | 0.553 | 0.737 | -25 | Contraction |
|  |  | 2061-2080 | 0.555 | 0.737 | -24.62 | Contraction |
|  |  | 2081-2100 | 0.555 | 0.737 | -24.64 | Contraction |
| *Hordeum stenostachys* Godr. | ssp126 | 2041-2060 | 0.493 | 0.739 | -33.28 | Contraction |
|  |  | 2061-2080 | 0.507 | 0.739 | -31.43 | Contraction |
|  |  | 2081-2100 | 0.507 | 0.739 | -31.45 | Contraction |
|  | ssp245 | 2041-2060 | 0.501 | 0.739 | -32.22 | Contraction |
|  |  | 2061-2080 | 0.503 | 0.739 | -31.93 | Contraction |
|  |  | 2081-2100 | 0.498 | 0.739 | -32.6 | Contraction |
|  | ssp370 | 2041-2060 | 0.504 | 0.739 | -31.8 | Contraction |
|  |  | 2061-2080 | 0.500 | 0.739 | -32.32 | Contraction |
|  |  | 2081-2100 | 0.502 | 0.739 | -32.11 | Contraction |
|  | ssp585 | 2041-2060 | 0.499 | 0.739 | -32.49 | Contraction |
|  |  | 2061-2080 | 0.494 | 0.739 | -33.22 | Contraction |
|  |  | 2081-2100 | 0.505 | 0.739 | -31.74 | Contraction |

**Supplementary Table 7.** Rate of change in potential suitable habitat area of *Hordeum* species under future climate scenarios.

| Specise | Period | Scenario | Current area (×10^6^km^2^) | Future area (×10^6^km^2^) | Area Change (×10^6^km^2^) | Rate of area change(%) | Direction of change |
| --- | --- | --- | --- | --- | --- | --- | --- |
| *Hordeum agriocrithon* A. E. Åberg | 2041-2060 | SSP1-2.6 | 89.83 | 123.80 | 33.97 | 37.82 | Expansion |
|  |  | SSP2-4.5 | 89.83 | 122.57 | 32.74 | 36.45 | Expansion |
|  |  | SSP3-7.0 | 89.83 | 121.92 | 32.09 | 35.73 | Expansion |
|  |  | SSP5-8.5 | 89.83 | 121.80 | 31.98 | 35.6 | Expansion |
|  | 2061-2080 | SSP1-2.6 | 89.83 | 123.51 | 33.69 | 37.5 | Expansion |
|  |  | SSP2-4.5 | 89.83 | 121.39 | 31.56 | 35.14 | Expansion |
|  |  | SSP3-7.0 | 89.83 | 119.36 | 29.53 | 32.88 | Expansion |
|  |  | SSP5-8.5 | 89.83 | 119.33 | 29.51 | 32.85 | Expansion |
|  | 2081-2100 | SSP1-2.6 | 89.83 | 123.86 | 34.04 | 37.89 | Expansion |
|  |  | SSP2-4.5 | 89.83 | 120.74 | 30.92 | 34.42 | Expansion |
|  |  | SSP3-7.0 | 89.83 | 117.87 | 28.04 | 31.22 | Expansion |
|  |  | SSP5-8.5 | 89.83 | 117.27 | 27.44 | 30.54 | Expansion |
| *Hordeum bogdanii* Wilensky | 2041-2060 | SSP1-2.6 | 52.78 | 17.72 | -35.06 | -66.43 | Contraction |
|  |  | SSP2-4.5 | 52.78 | 17.69 | -35.09 | -66.49 | Contraction |
|  |  | SSP3-7.0 | 52.78 | 17.30 | -35.48 | -67.22 | Contraction |
|  |  | SSP5-8.5 | 52.78 | 17.29 | -35.49 | -67.24 | Contraction |
|  | 2061-2080 | SSP1-2.6 | 52.78 | 17.35 | -35.42 | -67.12 | Contraction |
|  |  | SSP2-4.5 | 52.78 | 17.11 | -35.67 | -67.59 | Contraction |
|  |  | SSP3-7.0 | 52.78 | 16.97 | -35.81 | -67.85 | Contraction |
|  |  | SSP5-8.5 | 52.78 | 17.29 | -35.49 | -67.24 | Contraction |
|  | 2081-2100 | SSP1-2.6 | 52.78 | 17.47 | -35.31 | -66.9 | Contraction |
|  |  | SSP2-4.5 | 52.78 | 16.93 | -35.85 | -67.92 | Contraction |
|  |  | SSP3-7.0 | 52.78 | 16.45 | -36.33 | -68.83 | Contraction |
|  |  | SSP5-8.5 | 52.78 | 17.13 | -35.64 | -67.54 | Contraction |
| *Hordeum brevisubulatum* (Trin.) Link | 2041-2060 | SSP1-2.6 | 47.32 | 4.71 | -42.61 | -90.04 | Contraction |
|  |  | SSP2-4.5 | 47.32 | 4.70 | -42.62 | -90.06 | Contraction |
|  |  | SSP3-7.0 | 47.32 | 4.85 | -42.47 | -89.75 | Contraction |
|  |  | SSP5-8.5 | 47.32 | 4.82 | -42.50 | -89.82 | Contraction |
|  | 2061-2080 | SSP1-2.6 | 47.32 | 4.77 | -42.55 | -89.91 | Contraction |
|  |  | SSP2-4.5 | 47.32 | 4.73 | -42.59 | -90 | Contraction |
|  |  | SSP3-7.0 | 47.32 | 4.78 | -42.54 | -89.91 | Contraction |
|  |  | SSP5-8.5 | 47.32 | 5.06 | -42.26 | -89.31 | Contraction |
|  | 2081-2100 | SSP1-2.6 | 47.32 | 4.71 | -42.61 | -90.04 | Contraction |
|  |  | SSP2-4.5 | 47.32 | 4.87 | -42.45 | -89.7 | Contraction |
|  |  | SSP3-7.0 | 47.32 | 4.94 | -42.38 | -89.56 | Contraction |
|  |  | SSP5-8.5 | 47.32 | 5.03 | -42.29 | -89.38 | Contraction |
| *Hordeum bulbosum* L. | 2041-2060 | SSP1-2.6 | 33.02 | 9.37 | -23.66 | -71.63 | Contraction |
|  |  | SSP2-4.5 | 33.02 | 9.63 | -23.39 | -70.84 | Contraction |
|  |  | SSP3-7.0 | 33.02 | 9.57 | -23.46 | -71.03 | Contraction |
|  |  | SSP5-8.5 | 33.02 | 9.52 | -23.50 | -71.16 | Contraction |
|  | 2061-2080 | SSP1-2.6 | 33.02 | 9.25 | -23.78 | -72 | Contraction |
|  |  | SSP2-4.5 | 33.02 | 9.54 | -23.49 | -71.13 | Contraction |
|  |  | SSP3-7.0 | 33.02 | 9.76 | -23.27 | -70.45 | Contraction |
|  |  | SSP5-8.5 | 33.02 | 9.99 | -23.04 | -69.75 | Contraction |
|  | 2081-2100 | SSP1-2.6 | 33.02 | 9.13 | -23.89 | -72.35 | Contraction |
|  |  | SSP2-4.5 | 33.02 | 9.56 | -23.46 | -71.05 | Contraction |
|  |  | SSP3-7.0 | 33.02 | 9.68 | -23.34 | -70.68 | Contraction |
|  |  | SSP5-8.5 | 33.02 | 9.85 | -23.18 | -70.19 | Contraction |
| *Hordeum californicum* Covas & Stebbins | 2041-2060 | SSP1-2.6 | 99.28 | 17.50 | -81.78 | -82.37 | Contraction |
|  |  | SSP2-4.5 | 99.28 | 17.70 | -81.58 | -82.17 | Contraction |
|  |  | SSP3-7.0 | 99.28 | 17.47 | -81.81 | -82.4 | Contraction |
|  |  | SSP5-8.5 | 99.28 | 17.50 | -81.78 | -82.38 | Contraction |
|  | 2061-2080 | SSP1-2.6 | 99.28 | 17.35 | -81.93 | -82.52 | Contraction |
|  |  | SSP2-4.5 | 99.28 | 17.58 | -81.70 | -82.29 | Contraction |
|  |  | SSP3-7.0 | 99.28 | 17.55 | -81.73 | -82.32 | Contraction |
|  |  | SSP5-8.5 | 99.28 | 17.85 | -81.43 | -82.02 | Contraction |
|  | 2081-2100 | SSP1-2.6 | 99.28 | 17.24 | -82.04 | -82.63 | Contraction |
|  |  | SSP2-4.5 | 99.28 | 17.70 | -81.58 | -82.17 | Contraction |
|  |  | SSP3-7.0 | 99.28 | 17.74 | -81.55 | -82.14 | Contraction |
|  |  | SSP5-8.5 | 99.28 | 17.80 | -81.48 | -82.07 | Contraction |
| *Hordeum chilense* Roem. & Schult. | 2041-2060 | SSP1-2.6 | 134.42 | 45.72 | -88.70 | -65.98 | Contraction |
|  |  | SSP2-4.5 | 134.42 | 46.21 | -88.22 | -65.63 | Contraction |
|  |  | SSP3-7.0 | 134.42 | 45.79 | -88.63 | -65.94 | Contraction |
|  |  | SSP5-8.5 | 134.42 | 46.39 | -88.04 | -65.49 | Contraction |
|  | 2061-2080 | SSP1-2.6 | 134.42 | 45.90 | -88.52 | -65.85 | Contraction |
|  |  | SSP2-4.5 | 134.42 | 45.60 | -88.82 | -66.08 | Contraction |
|  |  | SSP3-7.0 | 134.42 | 45.41 | -89.02 | -66.22 | Contraction |
|  |  | SSP5-8.5 | 134.42 | 45.99 | -88.44 | -65.79 | Contraction |
|  | 2081-2100 | SSP1-2.6 | 134.42 | 46.45 | -87.98 | -65.45 | Contraction |
|  |  | SSP2-4.5 | 134.42 | 45.20 | -89.22 | -66.37 | Contraction |
|  |  | SSP3-7.0 | 134.42 | 45.89 | -88.54 | -65.87 | Contraction |
|  |  | SSP5-8.5 | 134.42 | 46.02 | -88.41 | -65.77 | Contraction |
| *Hordeum marinum* Huds. | 2041-2060 | SSP1-2.6 | 29.06 | 3.41 | -25.66 | -88.27 | Contraction |
|  |  | SSP2-4.5 | 29.06 | 3.50 | -25.56 | -87.94 | Contraction |
|  |  | SSP3-7.0 | 29.06 | 3.57 | -25.49 | -87.71 | Contraction |
|  |  | SSP5-8.5 | 29.06 | 3.54 | -25.53 | -87.83 | Contraction |
|  | 2061-2080 | SSP1-2.6 | 29.06 | 3.51 | -25.55 | -87.91 | Contraction |
|  |  | SSP2-4.5 | 29.06 | 3.75 | -25.32 | -87.1 | Contraction |
|  |  | SSP3-7.0 | 29.06 | 3.78 | -25.29 | -87 | Contraction |
|  |  | SSP5-8.5 | 29.06 | 3.69 | -25.37 | -87.3 | Contraction |
|  | 2081-2100 | SSP1-2.6 | 29.06 | 3.29 | -25.77 | -88.66 | Contraction |
|  |  | SSP2-4.5 | 29.06 | 3.71 | -25.35 | -87.22 | Contraction |
|  |  | SSP3-7.0 | 29.06 | 3.72 | -25.35 | -87.21 | Contraction |
|  |  | SSP5-8.5 | 29.06 | 4.06 | -25.00 | -86.02 | Contraction |
| *Hordeum murinum* L. | 2041-2060 | SSP1-2.6 | 54.54 | 11.81 | -42.73 | -78.35 | Contraction |
|  |  | SSP2-4.5 | 54.54 | 11.98 | -42.56 | -78.03 | Contraction |
|  |  | SSP3-7.0 | 54.54 | 12.19 | -42.35 | -77.65 | Contraction |
|  |  | SSP5-8.5 | 54.54 | 12.24 | -42.30 | -77.56 | Contraction |
|  | 2061-2080 | SSP1-2.6 | 54.54 | 11.90 | -42.65 | -78.19 | Contraction |
|  |  | SSP2-4.5 | 54.54 | 12.34 | -42.20 | -77.37 | Contraction |
|  |  | SSP3-7.0 | 54.54 | 13.00 | -41.55 | -76.17 | Contraction |
|  |  | SSP5-8.5 | 54.54 | 13.26 | -41.28 | -75.68 | Contraction |
|  | 2081-2100 | SSP1-2.6 | 54.54 | 11.83 | -42.71 | -78.31 | Contraction |
|  |  | SSP2-4.5 | 54.54 | 12.48 | -42.07 | -77.12 | Contraction |
|  |  | SSP3-7.0 | 54.54 | 13.46 | -41.08 | -75.32 | Contraction |
|  |  | SSP5-8.5 | 54.54 | 13.98 | -40.57 | -74.37 | Contraction |
| *Hordeum muticum* J.Presl | 2041-2060 | SSP1-2.6 | 40.21 | 25.00 | -15.21 | -37.83 | Contraction |
|  |  | SSP2-4.5 | 40.21 | 25.48 | -14.73 | -36.62 | Contraction |
|  |  | SSP3-7.0 | 40.21 | 25.23 | -14.98 | -37.25 | Contraction |
|  |  | SSP5-8.5 | 40.21 | 25.77 | -14.44 | -35.91 | Contraction |
|  | 2061-2080 | SSP1-2.6 | 40.21 | 25.38 | -14.83 | -36.89 | Contraction |
|  |  | SSP2-4.5 | 40.21 | 25.19 | -15.02 | -37.35 | Contraction |
|  |  | SSP3-7.0 | 40.21 | 25.13 | -15.08 | -37.49 | Contraction |
|  |  | SSP5-8.5 | 40.21 | 25.55 | -14.65 | -36.44 | Contraction |
|  | 2081-2100 | SSP1-2.6 | 40.21 | 25.48 | -14.73 | -36.63 | Contraction |
|  |  | SSP2-4.5 | 40.21 | 24.74 | -15.47 | -38.47 | Contraction |
|  |  | SSP3-7.0 | 40.21 | 25.47 | -14.73 | -36.64 | Contraction |
|  |  | SSP5-8.5 | 40.21 | 25.74 | -14.47 | -35.99 | Contraction |
| *Hordeum pusillum* Nutt. | 2041-2060 | SSP1-2.6 | 33.44 | 3.51 | -29.93 | -89.49 | Contraction |
|  |  | SSP2-4.5 | 33.44 | 3.51 | -29.93 | -89.5 | Contraction |
|  |  | SSP3-7.0 | 33.44 | 3.60 | -29.84 | -89.22 | Contraction |
|  |  | SSP5-8.5 | 33.44 | 3.58 | -29.86 | -89.29 | Contraction |
|  | 2061-2080 | SSP1-2.6 | 33.44 | 3.58 | -29.86 | -89.28 | Contraction |
|  |  | SSP2-4.5 | 33.44 | 3.65 | -29.79 | -89.08 | Contraction |
|  |  | SSP3-7.0 | 33.44 | 3.78 | -29.66 | -88.69 | Contraction |
|  |  | SSP5-8.5 | 33.44 | 3.78 | -29.66 | -88.7 | Contraction |
|  | 2081-2100 | SSP1-2.6 | 33.44 | 3.47 | -29.97 | -89.61 | Contraction |
|  |  | SSP2-4.5 | 33.44 | 3.63 | -29.81 | -89.13 | Contraction |
|  |  | SSP3-7.0 | 33.44 | 3.67 | -29.77 | -89.02 | Contraction |
|  |  | SSP5-8.5 | 33.44 | 4.01 | -29.43 | -88 | Contraction |
| *Hordeum roshevitzii* Bowden | 2041-2060 | SSP1-2.6 | 39.30 | 7.41 | -31.90 | -81.15 | Contraction |
|  |  | SSP2-4.5 | 39.30 | 7.11 | -32.19 | -81.9 | Contraction |
|  |  | SSP3-7.0 | 39.30 | 7.13 | -32.18 | -81.87 | Contraction |
|  |  | SSP5-8.5 | 39.30 | 6.95 | -32.35 | -82.32 | Contraction |
|  | 2061-2080 | SSP1-2.6 | 39.30 | 7.29 | -32.01 | -81.44 | Contraction |
|  |  | SSP2-4.5 | 39.30 | 6.90 | -32.40 | -82.44 | Contraction |
|  |  | SSP3-7.0 | 39.30 | 6.62 | -32.68 | -83.15 | Contraction |
|  |  | SSP5-8.5 | 39.30 | 6.63 | -32.68 | -83.14 | Contraction |
|  | 2081-2100 | SSP1-2.6 | 39.30 | 7.27 | -32.03 | -81.5 | Contraction |
|  |  | SSP2-4.5 | 39.30 | 6.68 | -32.62 | -83.01 | Contraction |
|  |  | SSP3-7.0 | 39.30 | 6.42 | -32.88 | -83.66 | Contraction |
|  |  | SSP5-8.5 | 39.30 | 6.56 | -32.74 | -83.3 | Contraction |
| *Hordeum spontaneum* K. Koch | 2041-2060 | SSP1-2.6 | 38.90 | 0.85 | -38.06 | -97.83 | Contraction |
|  |  | SSP2-4.5 | 38.90 | 0.78 | -38.13 | -98 | Contraction |
|  |  | SSP3-7.0 | 38.90 | 0.79 | -38.12 | -97.97 | Contraction |
|  |  | SSP5-8.5 | 38.90 | 0.78 | -38.13 | -98 | Contraction |
|  | 2061-2080 | SSP1-2.6 | 38.90 | 0.86 | -38.05 | -97.8 | Contraction |
|  |  | SSP2-4.5 | 38.90 | 0.97 | -37.93 | -97.5 | Contraction |
|  |  | SSP3-7.0 | 38.90 | 0.89 | -38.01 | -97.7 | Contraction |
|  |  | SSP5-8.5 | 38.90 | 0.85 | -38.05 | -97.8 | Contraction |
|  | 2081-2100 | SSP1-2.6 | 38.90 | 0.78 | -38.13 | -98 | Contraction |
|  |  | SSP2-4.5 | 38.90 | 0.87 | -38.03 | -97.75 | Contraction |
|  |  | SSP3-7.0 | 38.90 | 0.80 | -38.10 | -97.93 | Contraction |
|  |  | SSP5-8.5 | 38.90 | 0.92 | -37.98 | -97.63 | Contraction |
| *Hordeum stenostachys* Godr. | 2041-2060 | SSP1-2.6 | 32.82 | 2.27 | -30.55 | -93.09 | Contraction |
|  |  | SSP2-4.5 | 32.82 | 2.38 | -30.44 | -92.74 | Contraction |
|  |  | SSP3-7.0 | 32.82 | 2.28 | -30.55 | -93.07 | Contraction |
|  |  | SSP5-8.5 | 32.82 | 2.38 | -30.44 | -92.75 | Contraction |
|  | 2061-2080 | SSP1-2.6 | 32.82 | 2.33 | -30.49 | -92.89 | Contraction |
|  |  | SSP2-4.5 | 32.82 | 2.39 | -30.43 | -92.73 | Contraction |
|  |  | SSP3-7.0 | 32.82 | 2.41 | -30.41 | -92.65 | Contraction |
|  |  | SSP5-8.5 | 32.82 | 2.55 | -30.27 | -92.24 | Contraction |
|  | 2081-2100 | SSP1-2.6 | 32.82 | 2.30 | -30.52 | -92.98 | Contraction |
|  |  | SSP2-4.5 | 32.82 | 2.37 | -30.45 | -92.79 | Contraction |
|  |  | SSP3-7.0 | 32.82 | 2.43 | -30.39 | -92.58 | Contraction |
|  |  | SSP5-8.5 | 32.82 | 2.54 | -30.28 | -92.25 | Contraction |

**Supplementary Table 8.** Calculation results of niche overlap among *Hordeum* species.

| Species | Period | Scenario | Niche Overlap |
| --- | --- | --- | --- |
| *Hordeum agriocrithon* A. E. Åberg | 2041_2060 | ssp126 | 0.768 |
|  |  | ssp245 | 0.768 |
|  |  | ssp370 | 0.768 |
|  |  | ssp585 | 0.768 |
|  | 2061_2080 | ssp126 | 0.768 |
|  |  | ssp245 | 0.768 |
|  |  | ssp370 | 0.768 |
|  |  | ssp585 | 0.768 |
|  | 2081_2100 | ssp126 | 0.768 |
|  |  | ssp245 | 0.768 |
|  |  | ssp370 | 0.768 |
|  |  | ssp585 | 0.768 |
| *Hordeum bogdanii* Wilensky | 2041_2060 | ssp126 | 0.244 |
|  |  | ssp245 | 0.24 |
|  |  | ssp370 | 0.233 |
|  |  | ssp585 | 0.223 |
|  | 2061_2080 | ssp126 | 0.252 |
|  |  | ssp245 | 0.24 |
|  |  | ssp370 | 0.238 |
|  |  | ssp585 | 0.205 |
|  | 2081_2100 | ssp126 | 0.247 |
|  |  | ssp245 | 0.243 |
|  |  | ssp370 | 0.255 |
|  |  | ssp585 | 0.242 |
| *Hordeum brevisubulatum* (Trin.) Link | 2041_2060 | ssp126 | 0.28 |
|  |  | ssp245 | 0.274 |
|  |  | ssp370 | 0.268 |
|  |  | ssp585 | 0.269 |
|  | 2061_2080 | ssp126 | 0.28 |
|  |  | ssp245 | 0.28 |
|  |  | ssp370 | 0.267 |
|  |  | ssp585 | 0.244 |
|  | 2081_2100 | ssp126 | 0.286 |
|  |  | ssp245 | 0.273 |
|  |  | ssp370 | 0.293 |
|  |  | ssp585 | 0.272 |
| *Hordeum bulbosum* L. | 2041_2060 | ssp126 | 0.076 |
|  |  | ssp245 | 0.072 |
|  |  | ssp370 | 0.071 |
|  |  | ssp585 | 0.069 |
|  | 2061_2080 | ssp126 | 0.076 |
|  |  | ssp245 | 0.074 |
|  |  | ssp370 | 0.072 |
|  |  | ssp585 | 0.066 |
|  | 2081_2100 | ssp126 | 0.074 |
|  |  | ssp245 | 0.075 |
|  |  | ssp370 | 0.079 |
|  |  | ssp585 | 0.077 |
| *Hordeum californicum* Covas & Stebbins | 2041_2060 | ssp126 | 0.898 |
|  |  | ssp245 | 0.898 |
|  |  | ssp370 | 0.898 |
|  |  | ssp585 | 0.898 |
|  | 2061_2080 | ssp126 | 0.898 |
|  |  | ssp245 | 0.898 |
|  |  | ssp370 | 0.898 |
|  |  | ssp585 | 0.898 |
|  | 2081_2100 | ssp126 | 0.898 |
|  |  | ssp245 | 0.898 |
|  |  | ssp370 | 0.898 |
|  |  | ssp585 | 0.898 |
| *Hordeum chilense* Roem. & Schult. | 2041_2060 | ssp126 | 0.898 |
|  |  | ssp245 | 0.898 |
|  |  | ssp370 | 0.898 |
|  |  | ssp585 | 0.898 |
|  | 2061_2080 | ssp126 | 0.898 |
|  |  | ssp245 | 0.898 |
|  |  | ssp370 | 0.898 |
|  |  | ssp585 | 0.898 |
|  | 2081_2100 | ssp126 | 0.898 |
|  |  | ssp245 | 0.898 |
|  |  | ssp370 | 0.898 |
|  |  | ssp585 | 0.898 |
| *Hordeum marinum* Huds. | 2041_2060 | ssp126 | 0.066 |
|  |  | ssp245 | 0.064 |
|  |  | ssp370 | 0.069 |
|  |  | ssp585 | 0.07 |
|  | 2061_2080 | ssp126 | 0.073 |
|  |  | ssp245 | 0.065 |
|  |  | ssp370 | 0.069 |
|  |  | ssp585 | 0.071 |
|  | 2081_2100 | ssp126 | 0.064 |
|  |  | ssp245 | 0.074 |
|  |  | ssp370 | 0.073 |
|  |  | ssp585 | 0.088 |
| *Hordeum murinum* L. | 2041_2060 | ssp126 | 0.076 |
|  |  | ssp245 | 0.078 |
|  |  | ssp370 | 0.086 |
|  |  | ssp585 | 0.08 |
|  | 2061_2080 | ssp126 | 0.078 |
|  |  | ssp245 | 0.071 |
|  |  | ssp370 | 0.086 |
|  |  | ssp585 | 0.077 |
|  | 2081_2100 | ssp126 | 0.077 |
|  | 2081_2100 | ssp245 | 0.079 |
|  | 2081_2100 | ssp370 | 0.086 |
|  | 2081_2100 | ssp585 | 0.105 |
| *Hordeum muticum* J.Presl | 2041_2060 | ssp126 | 0.661 |
|  |  | ssp245 | 0.662 |
|  |  | ssp370 | 0.66 |
|  |  | ssp585 | 0.632 |
|  | 2061_2080 | ssp126 | 0.689 |
|  |  | ssp245 | 0.636 |
|  |  | ssp370 | 0.654 |
|  |  | ssp585 | 0.607 |
|  | 2081_2100 | ssp126 | 0.676 |
|  |  | ssp245 | 0.65 |
|  |  | ssp370 | 0.647 |
|  |  | ssp585 | 0.645 |
| *Hordeum pusillum* Nutt. | 2041_2060 | ssp126 | 0.057 |
|  |  | ssp245 | 0.06 |
|  |  | ssp370 | 0.059 |
|  |  | ssp585 | 0.058 |
|  | 2061_2080 | ssp126 | 0.061 |
|  |  | ssp245 | 0.063 |
|  |  | ssp370 | 0.063 |
|  |  | ssp585 | 0.067 |
|  | 2081_2100 | ssp126 | 0.053 |
|  |  | ssp245 | 0.066 |
|  |  | ssp370 | 0.07 |
|  |  | ssp585 | 0.08 |
| *Hordeum roshevitzii* Bowden | 2041_2060 | ssp126 | 0.165 |
|  |  | ssp245 | 0.146 |
|  |  | ssp370 | 0.151 |
|  |  | ssp585 | 0.138 |
|  | 2061_2080 | ssp126 | 0.17 |
|  |  | ssp245 | 0.158 |
|  |  | ssp370 | 0.158 |
|  |  | ssp585 | 0.114 |
|  | 2081_2100 | ssp126 | 0.16 |
|  |  | ssp245 | 0.177 |
|  |  | ssp370 | 0.18 |
|  |  | ssp585 | 0.161 |
| *Hordeum spontaneum* K. Koch | 2041_2060 | ssp126 | 0.064 |
|  |  | ssp245 | 0.062 |
|  |  | ssp370 | 0.056 |
|  |  | ssp585 | 0.057 |
|  | 2061_2080 | ssp126 | 0.066 |
|  |  | ssp245 | 0.059 |
|  |  | ssp370 | 0.06 |
|  |  | ssp585 | 0.055 |
|  | 2081_2100 | ssp126 | 0.061 |
|  |  | ssp245 | 0.064 |
|  |  | ssp370 | 0.065 |
|  |  | ssp585 | 0.069 |
| *Hordeum stenostachys* Godr. | 2041_2060 | ssp126 | 0.385 |
|  |  | ssp245 | 0.416 |
|  |  | ssp370 | 0.4 |
|  |  | ssp585 | 0.415 |
|  | 2061_2080 | ssp126 | 0.392 |
|  |  | ssp245 | 0.399 |
|  |  | ssp370 | 0.403 |
|  |  | ssp585 | 0.41 |
|  | 2081_2100 | ssp126 | 0.4 |
|  |  | ssp245 | 0.4 |
|  |  | ssp370 | 0.423 |
|  |  | ssp585 | 0.411 |

**Supplementary Table 9.** Summary of key niche overlap indices (e.g., I and D values) for *Hordeum* species.

| Species | Life History | Current Niche Width | Mean Overlap | CV Overlap | Max Overlap | Min Overlap |
| --- | --- | --- | --- | --- | --- | --- |
| *Hordeum agriocrithon* A. E. Åberg | Annual | 0.696 | 0.756 | 0.031 | 0.791 | 0.707 |
| *Hordeum bogdanii* Wilensky | Perennial | 0.679 | 0.241 | 0.082 | 0.27 | 0.206 |
| *Hordeum brevisubulatum* (Trin.) Link | Perennial | 0.648 | 0.296 | 0.066 | 0.316 | 0.243 |
| *Hordeum bulbosum* L. | Perennial | 0.688 | 0.063 | 0.101 | 0.072 | 0.052 |
| *Hordeum californicum* Covas & Stebbins | Annual | 0.754 | 0.901 | 0.022 | 0.925 | 0.863 |
| *Hordeum chilense* Roem. & Schult. | Annual | 0.77 | 0.905 | 0.012 | 0.922 | 0.889 |
| *Hordeum marinum* Huds. | Annual | 0.681 | 0.06 | 0.164 | 0.078 | 0.042 |
| *Hordeum murinum* L. | Annual | 0.707 | 0.074 | 0.143 | 0.089 | 0.047 |
| *Hordeum muticum* J.Presl | Perennial | 0.818 | 0.654 | 0.061 | 0.718 | 0.568 |
| *Hordeum pusillum* Nutt. | Annual | 0.692 | 0.063 | 0.206 | 0.088 | 0.053 |
| *Hordeum roshevitzii* Bowden | Perennial | 0.657 | 0.146 | 0.137 | 0.179 | 0.113 |
| *Hordeum spontaneum* K. Koch | Annual | 0.737 | 0.058 | 0.131 | 0.075 | 0.047 |
| *Hordeum stenostachys* Godr. | Perennial | 0.739 | 0.451 | 0.035 | 0.484 | 0.428 |

**Supplementary Table 10.** Geographic distribution centroid migration parameters of Hordeum species under different climate scenarios.

| Species | Scenario | Period | Lon-current | Lat-current | Lon-centroid | Lat-centroid | LonLat-Distance (km) | LonLat-Angle | LonLat-Direction |
| --- | --- | --- | --- | --- | --- | --- | --- | --- | --- |
| *Hordeum agriocrithon* A. E. Åberg | ssp126 | 2041-2060 | 31.923 | 39.168 | 19.283 | 40.058 | 12.671 | 175.974 | West |
|  |  | 2061-2080 |  |  | 19.407 | 40.078 | 12.549 | 175.843 | West |
|  |  | 2081-2100 |  |  | 19.436 | 39.911 | 12.509 | 176.594 | West |
|  | ssp245 | 2041-2060 |  |  | 19.416 | 40.207 | 12.549 | 175.249 | West |
|  |  | 2061-2080 |  |  | 19.228 | 40.449 | 12.759 | 174.239 | West |
|  |  | 2081-2100 |  |  | 19.162 | 40.617 | 12.843 | 173.522 | West |
|  | ssp370 | 2041-2060 |  |  | 19.251 | 40.505 | 12.742 | 173.978 | West |
|  |  | 2061-2080 |  |  | 19.304 | 40.888 | 12.735 | 172.237 | West |
|  |  | 2081-2100 |  |  | 19.469 | 41.272 | 12.630 | 170.409 | West |
|  | ssp585 | 2041-2060 |  |  | 19.475 | 40.378 | 12.506 | 174.449 | West |
|  |  | 2061-2080 |  |  | 19.236 | 40.991 | 12.817 | 171.821 | West |
|  |  | 2081-2100 |  |  | 19.450 | 41.453 | 12.680 | 169.620 | West |
| *Hordeum bogdanii* Wilensky | ssp126 | 2041-2060 | 29.148 | 48.473 | 18.979 | 30.484 | 20.664 | -119.479 | South |
|  |  | 2061-2080 |  |  | 17.449 | 31.407 | 20.691 | -124.432 | South |
|  |  | 2081-2100 |  |  | 19.698 | 30.006 | 20.744 | -117.100 | South |
|  | ssp245 | 2041-2060 |  |  | 18.902 | 30.059 | 21.073 | -119.092 | South |
|  |  | 2061-2080 |  |  | 18.126 | 30.393 | 21.174 | -121.369 | South |
|  |  | 2081-2100 |  |  | 20.129 | 29.386 | 21.110 | -115.292 | South |
|  | ssp370 | 2041-2060 |  |  | 19.675 | 30.099 | 20.672 | -117.273 | South |
|  |  | 2061-2080 |  |  | 21.730 | 29.001 | 20.837 | -110.856 | South |
|  |  | 2081-2100 |  |  | 24.015 | 26.878 | 22.196 | -103.371 | South |
|  | ssp585 | 2041-2060 |  |  | 19.779 | 29.153 | 21.472 | -115.872 | South |
|  |  | 2061-2080 |  |  | 19.734 | 29.006 | 21.623 | -115.809 | South |
|  |  | 2081-2100 |  |  | 21.433 | 28.263 | 21.632 | -110.894 | South |
| *Hordeum brevisubulatum* (Trin.) Link | ssp126 | 2041-2060 | 48.432 | 52.345 | 22.317 | 21.713 | 40.254 | -130.449 | South |
|  |  | 2061-2080 |  |  | 22.845 | 22.004 | 39.690 | -130.140 | South |
|  |  | 2081-2100 |  |  | 22.085 | 21.798 | 40.340 | -130.778 | South |
|  | ssp245 | 2041-2060 |  |  | 23.291 | 21.955 | 39.441 | -129.600 | South |
|  |  | 2061-2080 |  |  | 22.028 | 21.610 | 40.520 | -130.665 | South |
|  |  | 2081-2100 |  |  | 22.101 | 21.847 | 40.292 | -130.805 | South |
|  | ssp370 | 2041-2060 |  |  | 22.772 | 21.813 | 39.883 | -130.045 | South |
|  |  | 2061-2080 |  |  | 21.763 | 21.662 | 40.653 | -130.997 | South |
|  |  | 2081-2100 |  |  | 20.831 | 21.487 | 41.401 | -131.811 | South |
|  | ssp585 | 2041-2060 |  |  | 22.478 | 21.781 | 40.097 | -130.337 | South |
|  |  | 2061-2080 |  |  | 23.579 | 22.336 | 38.965 | -129.630 | South |
|  |  | 2081-2100 |  |  | 21.686 | 21.669 | 40.699 | -131.084 | South |
| *Hordeum bulbosum* L. | ssp126 | 2041-2060 | 5.686 | 36.178 | 29.899 | 28.864 | 25.293 | -16.809 | East |
|  |  | 2061-2080 |  |  | 30.265 | 29.138 | 25.567 | -15.983 | East |
|  |  | 2081-2100 |  |  | 30.025 | 28.928 | 25.395 | -16.588 | East |
|  | ssp245 | 2041-2060 |  |  | 30.037 | 28.576 | 25.510 | -17.338 | East |
|  |  | 2061-2080 |  |  | 29.890 | 28.181 | 25.491 | -18.284 | East |
|  |  | 2081-2100 |  |  | 30.130 | 27.981 | 25.782 | -18.539 | East |
|  | ssp370 | 2041-2060 |  |  | 30.089 | 28.648 | 25.538 | -17.150 | East |
|  |  | 2061-2080 |  |  | 31.134 | 27.817 | 26.786 | -18.189 | East |
|  |  | 2081-2100 |  |  | 31.870 | 27.246 | 27.665 | -18.837 | East |
|  | ssp585 | 2041-2060 |  |  | 30.371 | 28.218 | 25.936 | -17.873 | East |
|  |  | 2061-2080 |  |  | 30.896 | 27.812 | 26.562 | -18.360 | East |
|  |  | 2081-2100 |  |  | 30.997 | 27.388 | 26.794 | -19.151 | East |
| *Hordeum californicum* Covas & Stebbins | ssp126 | 2041-2060 | 8.460 | 34.926 | 27.552 | 28.068 | 20.286 | -19.760 | East |
|  |  | 2061-2080 |  |  | 27.472 | 28.100 | 20.200 | -19.752 | East |
|  |  | 2081-2100 |  |  | 27.564 | 28.190 | 20.256 | -19.423 | East |
|  | ssp245 | 2041-2060 |  |  | 27.616 | 27.934 | 20.393 | -20.054 | East |
|  |  | 2061-2080 |  |  | 27.497 | 27.992 | 20.260 | -20.015 | East |
|  |  | 2081-2100 |  |  | 27.253 | 28.020 | 20.022 | -20.177 | East |
|  | ssp370 | 2041-2060 |  |  | 27.423 | 28.122 | 20.147 | -19.738 | East |
|  |  | 2061-2080 |  |  | 27.117 | 27.867 | 19.948 | -20.726 | East |
|  |  | 2081-2100 |  |  | 27.552 | 28.084 | 20.281 | -19.718 | East |
|  | ssp585 | 2041-2060 |  |  | 27.426 | 28.054 | 20.173 | -19.917 | East |
|  |  | 2061-2080 |  |  | 27.504 | 27.979 | 20.272 | -20.042 | East |
|  |  | 2081-2100 |  |  | 27.192 | 27.915 | 20.001 | -20.520 | East |
| *Hordeum chilense* Roem. & Schult. | ssp126 | 2041-2060 | 10.007 | 29.014 | 24.132 | 28.218 | 14.148 | -3.225 | East |
|  |  | 2061-2080 |  |  | 24.102 | 28.180 | 14.120 | -3.383 | East |
|  |  | 2081-2100 |  |  | 24.401 | 28.265 | 14.413 | -2.975 | East |
|  | ssp245 | 2041-2060 |  |  | 24.427 | 28.127 | 14.447 | -3.519 | East |
|  |  | 2061-2080 |  |  | 24.048 | 28.160 | 14.067 | -3.480 | East |
|  |  | 2081-2100 |  |  | 24.013 | 28.229 | 14.028 | -3.206 | East |
|  | ssp370 | 2041-2060 |  |  | 24.097 | 28.240 | 14.112 | -3.144 | East |
|  |  | 2061-2080 |  |  | 23.938 | 28.037 | 13.966 | -4.008 | East |
|  |  | 2081-2100 |  |  | 23.907 | 28.195 | 13.925 | -3.370 | East |
|  | ssp585 | 2041-2060 |  |  | 24.088 | 28.073 | 14.112 | -3.823 | East |
|  |  | 2061-2080 |  |  | 23.839 | 28.037 | 13.867 | -4.037 | East |
|  |  | 2081-2100 |  |  | 23.706 | 27.907 | 13.744 | -4.617 | East |
| *Hordeum marinum* Huds. | ssp126 | 2041-2060 | 5.288 | 27.771 | 32.427 | 26.537 | 27.167 | -2.603 | East |
|  |  | 2061-2080 |  |  | 33.179 | 26.778 | 27.908 | -2.038 | East |
|  |  | 2081-2100 |  |  | 32.321 | 26.764 | 27.052 | -2.133 | East |
|  | ssp245 | 2041-2060 |  |  | 31.795 | 26.551 | 26.535 | -2.635 | East |
|  |  | 2061-2080 |  |  | 32.380 | 26.105 | 27.144 | -3.519 | East |
|  |  | 2081-2100 |  |  | 32.234 | 26.366 | 26.983 | -2.983 | East |
|  | ssp370 | 2041-2060 |  |  | 32.245 | 26.610 | 26.982 | -2.466 | East |
|  |  | 2061-2080 |  |  | 32.577 | 26.123 | 27.339 | -3.454 | East |
|  |  | 2081-2100 |  |  | 32.605 | 25.668 | 27.398 | -4.400 | East |
|  | ssp585 | 2041-2060 |  |  | 32.196 | 26.527 | 26.937 | -2.646 | East |
|  |  | 2061-2080 |  |  | 31.112 | 25.962 | 25.887 | -4.007 | East |
|  |  | 2081-2100 |  |  | 31.808 | 25.709 | 26.601 | -4.445 | East |
| *Hordeum murinum* L. | ssp126 | 2041-2060 | 11.107 | 25.869 | 28.789 | 21.689 | 18.169 | -13.300 | East |
|  |  | 2061-2080 |  |  | 28.518 | 22.412 | 17.751 | -11.228 | East |
|  |  | 2081-2100 |  |  | 28.646 | 21.785 | 18.009 | -13.109 | East |
|  | ssp245 | 2041-2060 |  |  | 27.646 | 21.847 | 17.021 | -13.667 | East |
|  |  | 2061-2080 |  |  | 28.207 | 21.707 | 17.600 | -13.680 | East |
|  |  | 2081-2100 |  |  | 27.651 | 22.077 | 16.973 | -12.908 | East |
|  | ssp370 | 2041-2060 |  |  | 27.898 | 22.003 | 17.231 | -12.966 | East |
|  |  | 2061-2080 |  |  | 28.194 | 21.901 | 17.541 | -13.073 | East |
|  |  | 2081-2100 |  |  | 28.971 | 21.295 | 18.440 | -14.361 | East |
|  | ssp585 | 2041-2060 |  |  | 27.666 | 22.139 | 16.974 | -12.693 | East |
|  |  | 2061-2080 |  |  | 27.854 | 21.829 | 17.227 | -13.561 | East |
|  |  | 2081-2100 |  |  | 28.528 | 21.715 | 17.909 | -13.410 | East |
| *Hordeum muticum* J.Presl | ssp126 | 2041-2060 | -2.327 | 14.321 | 49.296 | 14.002 | 51.624 | -0.354 | East |
|  |  | 2061-2080 |  |  | 49.512 | 14.092 | 51.839 | -0.253 | East |
|  |  | 2081-2100 |  |  | 50.365 | 14.200 | 52.692 | -0.132 | East |
|  | ssp245 | 2041-2060 |  |  | 49.948 | 13.958 | 52.276 | -0.397 | East |
|  |  | 2061-2080 |  |  | 48.767 | 13.711 | 51.098 | -0.683 | East |
|  |  | 2081-2100 |  |  | 49.627 | 13.792 | 51.957 | -0.583 | East |
|  | ssp370 | 2041-2060 |  |  | 49.587 | 14.087 | 51.915 | -0.258 | East |
|  |  | 2061-2080 |  |  | 48.699 | 13.796 | 51.029 | -0.589 | East |
|  |  | 2081-2100 |  |  | 48.743 | 14.445 | 51.071 | 0.139 | East |
|  | ssp585 | 2041-2060 |  |  | 49.044 | 13.761 | 51.375 | -0.624 | East |
|  |  | 2061-2080 |  |  | 48.393 | 13.609 | 50.726 | -0.804 | East |
|  |  | 2081-2100 |  |  | 47.864 | 13.387 | 50.199 | -1.066 | East |
| *Hordeum pusillum* Nutt. | ssp126 | 2041-2060 | -24.754 | 31.522 | 24.463 | 18.789 | 50.837 | -14.505 | East |
|  |  | 2061-2080 |  |  | 24.457 | 19.340 | 50.696 | -13.904 | East |
|  |  | 2081-2100 |  |  | 24.949 | 18.930 | 51.273 | -14.216 | East |
|  | ssp245 | 2041-2060 |  |  | 23.140 | 18.774 | 49.561 | -14.904 | East |
|  |  | 2061-2080 |  |  | 23.748 | 18.723 | 50.162 | -14.782 | East |
|  |  | 2081-2100 |  |  | 23.578 | 18.418 | 50.077 | -15.169 | East |
|  | ssp370 | 2041-2060 |  |  | 23.744 | 18.843 | 50.128 | -14.651 | East |
|  |  | 2061-2080 |  |  | 24.339 | 17.949 | 50.934 | -15.455 | East |
|  |  | 2081-2100 |  |  | 21.606 | 16.836 | 48.630 | -17.577 | East |
|  | ssp585 | 2041-2060 |  |  | 23.066 | 17.991 | 49.697 | -15.799 | East |
|  |  | 2061-2080 |  |  | 21.998 | 17.665 | 48.762 | -16.509 | East |
|  |  | 2081-2100 |  |  | 21.984 | 16.700 | 49.032 | -17.595 | East |
| *Hordeum roshevitzii* Bowden | ssp126 | 2041-2060 | 57.774 | 51.611 | 37.117 | 37.013 | 25.295 | -144.753 | West |
|  |  | 2061-2080 |  |  | 34.944 | 38.116 | 26.521 | -149.412 | West |
|  |  | 2081-2100 |  |  | 37.013 | 36.579 | 25.632 | -144.095 | West |
|  | ssp245 | 2041-2060 |  |  | 34.882 | 37.224 | 27.038 | -147.852 | West |
|  |  | 2061-2080 |  |  | 36.113 | 37.334 | 25.943 | -146.610 | West |
|  |  | 2081-2100 |  |  | 39.276 | 36.100 | 24.141 | -140.020 | West |
|  | ssp370 | 2041-2060 |  |  | 38.384 | 36.910 | 24.333 | -142.832 | West |
|  |  | 2061-2080 |  |  | 41.824 | 36.110 | 22.242 | -135.819 | West |
|  |  | 2081-2100 |  |  | 45.536 | 33.613 | 21.764 | -124.215 | South |
|  | ssp585 | 2041-2060 |  |  | 38.341 | 35.801 | 25.052 | -140.871 | West |
|  |  | 2061-2080 |  |  | 39.283 | 36.197 | 24.073 | -140.186 | West |
|  |  | 2081-2100 |  |  | 42.957 | 35.405 | 21.959 | -132.436 | South |
| *Hordeum spontaneum* K. Koch | ssp126 | 2041-2060 | 21.583 | 35.463 | 18.387 | 30.617 | 5.805 | -123.407 | South |
|  |  | 2061-2080 |  |  | 20.269 | 31.044 | 4.611 | -106.564 | South |
|  |  | 2081-2100 |  |  | 18.505 | 30.641 | 5.721 | -122.560 | South |
|  | ssp245 | 2041-2060 |  |  | 17.880 | 30.257 | 6.389 | -125.429 | South |
|  |  | 2061-2080 |  |  | 18.672 | 29.436 | 6.694 | -115.784 | South |
|  |  | 2081-2100 |  |  | 19.327 | 29.019 | 6.828 | -109.294 | South |
|  | ssp370 | 2041-2060 |  |  | 17.851 | 30.641 | 6.098 | -127.744 | South |
|  |  | 2061-2080 |  |  | 21.163 | 28.477 | 6.998 | -93.446 | South |
|  |  | 2081-2100 |  |  | 22.719 | 27.188 | 8.352 | -82.188 | South |
|  | ssp585 | 2041-2060 |  |  | 18.880 | 29.739 | 6.331 | -115.282 | South |
|  |  | 2061-2080 |  |  | 19.943 | 28.422 | 7.229 | -103.111 | South |
|  |  | 2081-2100 |  |  | 21.718 | 27.905 | 7.559 | -88.981 | South |
| *Hordeum stenostachys* Godr. | ssp126 | 2041-2060 | -2.825 | 4.157 | 19.530 | 16.954 | 25.759 | 29.789 | East |
|  |  | 2061-2080 |  |  | 19.109 | 17.492 | 25.670 | 31.299 | East |
|  |  | 2081-2100 |  |  | 19.900 | 16.818 | 26.014 | 29.125 | East |
|  | ssp245 | 2041-2060 |  |  | 18.707 | 16.857 | 24.999 | 30.534 | East |
|  |  | 2061-2080 |  |  | 19.304 | 16.765 | 25.469 | 29.674 | East |
|  |  | 2081-2100 |  |  | 17.205 | 17.665 | 24.160 | 33.995 | East |
|  | ssp370 | 2041-2060 |  |  | 17.993 | 17.476 | 24.714 | 32.610 | East |
|  |  | 2061-2080 |  |  | 16.236 | 18.050 | 23.587 | 36.088 | East |
|  |  | 2081-2100 |  |  | 17.083 | 17.646 | 24.048 | 34.120 | East |
|  | ssp585 | 2041-2060 |  |  | 18.574 | 16.913 | 24.913 | 30.801 | East |
|  |  | 2061-2080 |  |  | 17.455 | 17.082 | 24.049 | 32.511 | East |
|  |  | 2081-2100 |  |  | 17.185 | 16.791 | 23.666 | 32.268 | East |
